# Supplementary material for: Latent classes of unhealthy behaviours and their associations with subsequent sickness absence: a prospective register-linkage study among Finnish young and early midlife employees
Source: BMJ Open. 2023 May 11;13(5):e070883. doi: 10.1136/bmjopen-2022-070883 (PMC10186467; doi:10.1136/bmjopen-2022-070883)
Supplement: Supplementary data [file bmjopen-2022-070883supp001.pdf]

SUPPLEMENTAL FILE 1

Table S1. Model fit statistics of latent classes of unhealthy behaviours among women and men.

Number of latent classes	Class membership based on posterior probabilities, n (%)	Marginal probabilities of class membership	Average posterior probabilities	Average posterior probabilities of class membership in each class	AIC value	BIC value	Entropy
**Women**							
1	3228 (100)	1.0	1.00	1.00	16715.2	16745.6	1.00
2	2281 (70.7)	0.65	0.87	0.87	16402.5	16469.4	0.86
	947 (29.3)	0.35		0.88			
3	2701 (83.7)	0.75	0.85	0.87	16366.4	16469.8	0.86
	147 (4.6)	0.08		0.63			
	380 (11.8)	0.17		0.82			
4	1881 (58.3)	0.57	0.81	0.87	16374.7	16508.5	0.83
	781 (24.2)	0.21		0.71			
	128 (4.0)	0.07		0.66			
	438 (13.6)	0.15		0.75			
5	1628 (50.4)	0.44	0.83	0.85	16371.3	16535.4	0.90
	846 (26.2)	0.24		0.82			
	199 (6.2)	0.10		0.60			
	497 (15.4)	0.21		0.89			
	58 (1.8)	0.02		0.67			
**Men**							
1	774 (100)	1.00	1.00	1.00	4723.9	4747.2	1.00
2	412 (53.2)	0.45	0.86	0.78	4650.5	4701.7	0.88
	362 (46.8)	0.55		0.94			
3	445 (57.5)	0.58	0.96	0.99	4643.0	4722.1	0.98
	39 (5.0)	0.05		0.66			
	290 (37.5)	0.37		0.96			
4	91 (11.8)	0.22	0.76	0.84	4648.8	4751.1	0.83
	421 (54.4)	0.38		0.69			
	159 (20.5)	0.30		0.94			
	103 (13.3)	0.10		0.71			
5	20 (2.6)	0.13	0.71	0.73	4644.5	4746.8	0.90
	171 (22.1)	1.18		0.74			
	325 (42.0)	0.31		0.68			

BMJ Publishing Group Limited (BMJ) disclaims all liability and responsibility arising from any reliance placed on this supplemental material which has been supplied by the author(s)

	167 (21.6)	0.16	0.67
	91 (11.8)	0.22	0.85

AIC = Akaike Information Criterion, BIC = Bayesian Information Criterion

Table S2. Latent class marginal means with 95% confidence intervals (CI) for unhealthy behaviours by latent classes among women and men.

Unhealthy behaviours within latent classes	Women	Men
	**Marginal mean (95% CI)**	**Marginal mean (95% CI)**
**Class 1**		
Low LTPA	0.13 (0.11-0.15)	0.10 (0.06-0.16)
Non-daily F&V consumption	0.13 (0.11-0.16)	0.33 (0.26-0.40)
Insufficient sleep	0.31 (0.28-0.33)	0.32 (0.26-0.38)
Excessive alcohol use	0.08 (0.03-0.16)	0.21 (0.10-0.39)
Tobacco use	0.12 (0.06-0.22)	0.00 (0.00-1.00)
**Class 2**		
Low LTPA	0.11 (0.07-0.18)	0.02 (0.00-0.92)
Non-daily F&V consumption	0.26 (0.19-0.34)	0.12 (0.01-0.70)
Insufficient sleep	0.31 (0.24-0.38)	0.29 (0.21-0.38)
Excessive alcohol use	0.77 (0.17-0.98)	0.99 (0.00-1.00)
Tobacco use	0.69 (0.42-0.87)	0.51 (0.40-0.61)
**Class 3**		
Low physical activity	0.51 (0.27-0.74)	
Non-daily F&V consumption	0.63 (0.38-0.83)	
Insufficient sleep	0.61 (0.44-0.76)	
Excessive alcohol use	0.18 (0.07-0.41)	
Tobacco use	0.45 (0.28-0.63)	

F&V = fruit and vegetable, LTPA = leisure-time physical activity

BMJ Publishing Group Limited (BMJ) disclaims all liability and responsibility arising from any reliance placed on this supplemental material which has been supplied by the author(s)

Table S3. Sociodemographic characteristics (n, %) of latent classes of unhealthy behaviours among women and men.

Sociodemographic factors	Women				Men		
	Class 1 (n=2701)	Class 2 (n=380)	Class 3 (n=147)	P-value [a]	Class 1 (n=412)	Class 2 (n=362)	P-value [a]
**Age**				<0.001			0.098
19–29 years	827 (30.6)	176 (46.3)	46 (31.3)		93 (22.6)	104 (28.7)	
30–34 years	943 (34.9)	119 (31.3)	46 (31.3)		134 (32.5)	118 (32.6)	
35–39 years	931 (34.5)	85 (22.4)	55 (37.4)		185 (44.9)	140 (38.7)	
**Marital status**				<0.001			<0.001
Married or cohabiting	1857 (68.8)	174 (45.8)	91 (61.9)		328 (79.6)	242 (66.9)	
Other	844 (31.3)	206 (54.2)	56 (38.1)		84 (20.4)	120 (33.2)	
**Children living in the household**				<0.001			<0.001
No	1471 (54.5)	305 (80.3)	75 (51.0)		223 (54.1)	244 (67.4)	
Yes	1230 (45.5)	75 (19.7)	72 (49.0)		189 (45.9)	118 (32.6)	
**Occupational class**				<0.001			<0.001
Managers and professionals	802 (29.7)	71 (18.7)	22 (15.0)		154 (37.4)	87 (24.0)	
Semi-professionals	1167 (43.2)	173 (45.5)	62 (42.2)		129 (31.3)	113 (31.2)	
Routine non-manual workers	665 (24.6)	123 (32.4)	55 (37.4)		88 (21.4)	103 (28.5)	
Manual workers	67 (2.5)	13 (3.4)	8 (5.4)		41 (10.0)	59 (16.3)	

[a] P-values from Chi-Square Tests.

Table S4. Characteristics of all participants who responded to the Helsinki Health Study survey (2017), of those who gave their written consent to register linkages, of those who were excluded from this study [a], and of those who were finally included in this study.

	All participants who responded to the survey (n=5898)	Participants who gave their consent to register linkages (n=4864)	Excluded telephone interviewees [a] (n=651)	Other excluded participants [a] (n=211)	Participants in this study (n=4002)
**Total number of sickness absence days during the follow-up [b], median (interquartile range)**	Not available.	11 (25)	8 (24)	9 (29)	12 (26)
Health behaviours					

**Fresh vegetable consumption**					
Daily	4119 (70.1)	3443 (71.1)	493 (75.9)	141 (67.1)	2809 (70.5)
Non-daily	1755 (29.9)	1401 (28.9)	157 (24.2)	69 (32.9)	1175 (29.5)
**Sleep (hours)**					
7–9 hours	4573 (78.0)	3799 (78.6)	493 (75.7)	159 (76.4)	3147 (79.2)
<7 or >9 hours	1287 (22.0)	1035 (21.4)	158 (24.3)	49 (23.6)	828 (20.8)
**Alcohol use** [c]					
Once a week or less	5058 (88.9)	4153 (88.4)	610 (93.7)	179 (88.2)	3364 (87.5)
More than once a week	630 (11.1)	545 (11.6)	41 (6.3)	24 (11.8)	480 (12.5)
**Smoking** [d]					
No	4480 (76.5)	3707 (76.7)	518 (79.7)	153 (73.6)	3036 (76.4)
Currently or occasionally	1378 (23.5)	1126 (23.3)	132 (20.3)	55 (26.4)	939 (23.6)
Sociodemographic factors					
**Gender**					
Woman	4630 (78.5)	3848 (79.1)	461 (70.8)	159 (75.4)	3228 (80.7)
Man	1267 (21.5)	1016 (20.9)	190 (29.2)	52 (24.6)	774 (19.3)
**Age**					
19–29 years	1864 (31.7)	1532 (31.5)	204 (31.3)	82 (38.9)	1246 (31.1)
30–34 years	2000 (34.0)	1658 (34.1)	225 (34.6)	73 (34.6)	1360 (34.0)
35–39 years	2023 (34.4)	1674 (34.4)	222 (34.1)	56 (26.5)	1396 (34.9)
**Marital status**					
Married or cohabiting	3910 (66.3)	3248 (66.8)	427 (65.6)	129 (61.1)	2692 (67.3)
Other	1988 (33.7)	1616 (33.2)	224 (34.4)	82 (38.9)	1310 (32.7)
**Occupational class**					
Managers and professionals	1552 (27.1)	1346 (27.7)	165 (25.3)	45 (21.4)	1136 (28.4)
Semi-professionals	2233 (38.9)	1937 (39.8)	202 (31.0)	91 (43.1)	1644 (41.1)
Routine non-manual workers	1612 (28.1)	1309 (26.9)	219 (33.6)	56 (26.5)	1034 (25.8)
Manual workers	338 (5.9)	272 (5.6)	65 (10.0)	19 (9.0)	188 (4.7)

[a] Of all participants who gave their written consent to register linkages, this study excluded 1) telephone interviewees (n=651), 2) participants who had missing data on working time (n=33) after the data collection or on all health behaviours of interest (n=1), or 3) who had extreme values in health behaviours—that is, spending >24 hours together for leisure-time physical activity, sitting, and sleeping (n=166), or reporting >300 metabolic equivalent task -hours for leisure-time physical activity (n=11).

[b] The follow-up began from one day after receiving the completed survey questionnaire from a participant and continued until 31st March 2020 or until the end of one's employment contract, whichever came first.

[c] Participants were asked to estimate the frequency they currently use beer, wine, and spirits. The question included 10 alternatives from "never" to "daily or almost daily".

[d] Smoking included only cigarettes (not e-cigarettes or snus).

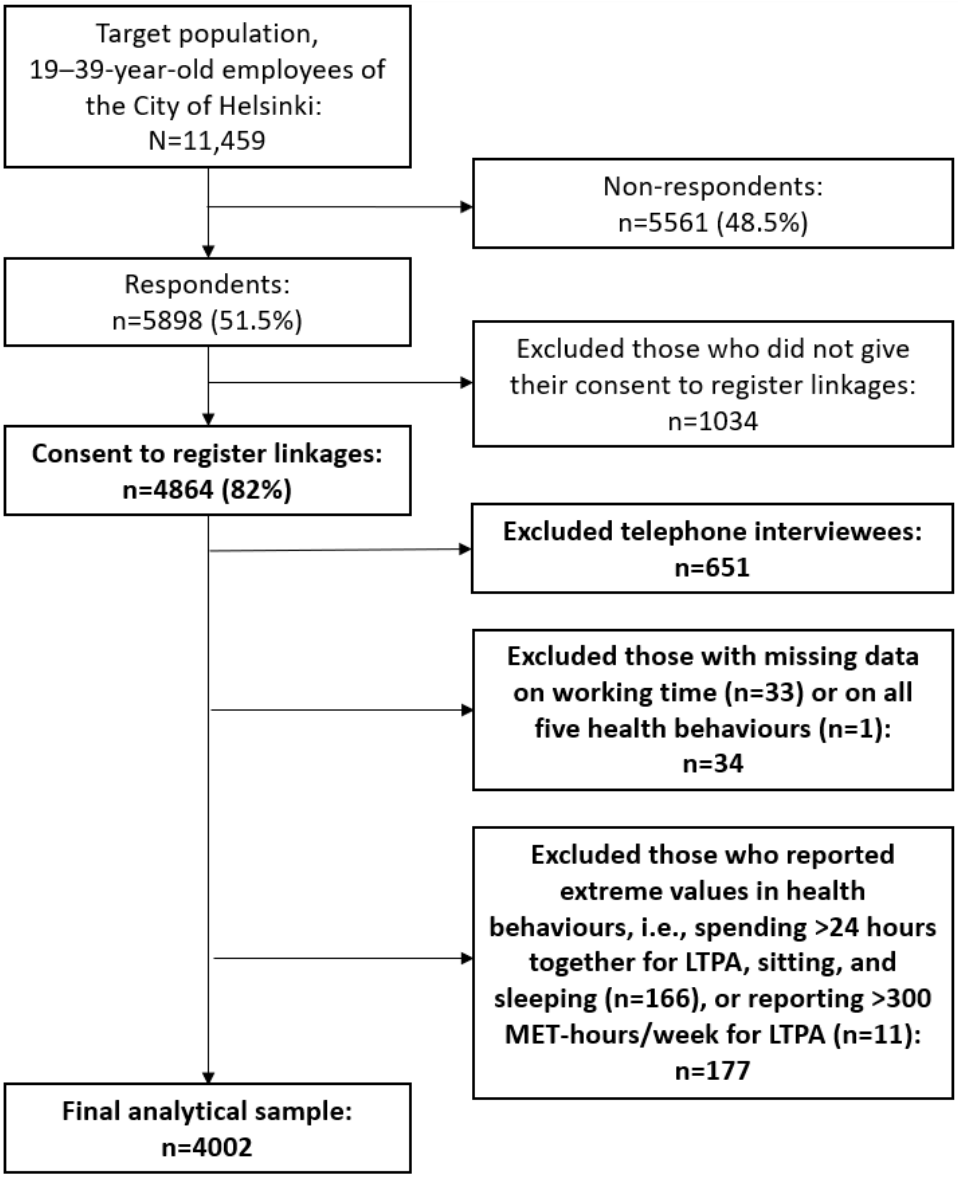

Figure S1. Flow chart: selection of the final analytical study sample.

LTPA = leisure-time physical activity, MET = metabolic equivalent task
