# Supplementary material for: Latent classes of unhealthy behaviours and their associations with subsequent sickness absence: a prospective register-linkage study among Finnish young and early midlife employees
Source: BMJ Open. 2023 May 11;13(5):e070883. doi: 10.1136/bmjopen-2022-070883 (PMC10186467; doi:10.1136/bmjopen-2022-070883)
Supplement: Supplementary data [file bmjopen-2022-070883supp002.pdf]

## SUPPLEMENTAL FILE 2

## Helsinki Health Study survey: questions on health behaviours

Leisure-time physical activity

**41. Next, we will be asking about physical activity during your leisure and commuting time over the past 12 months. We have divided physical activities in four levels of exertion. First, estimate the exertion level of the physical activities you are engaged in. Then, estimate how often you engage in a physical activity equivalent to each level of exertion during one week rounded to closest 15 minutes (e.g. 02 hours and 45 minutes).**

**a. During your leisure time**

| | Hours | Minutes |
|---|---|---|
| Strenuousness of exercise: | | |
| Equivalent to walking | ______ | ______ |
| Equivalent to brisk walking | ______ | ______ |
| Equivalent to light running (jogging) | ______ | ______ |
| Equivalent to brisk running | ______ | ______ |

**b. During your commute**

| | Hours | Minutes |
|---|---|---|
| Strenuousness of exercise: | | |
| Equivalent to walking | ______ | ______ |
| Equivalent to brisk walking | ______ | ______ |
| Equivalent to light running (jogging) | ______ | ______ |
| Equivalent to brisk running | ______ | ______ |

Diet

**39. How often do you consume the following food items? Think about the past four weeks. Please choose one alternative in each line.**

| | Not in the past 4 weeks | 1–3 times a month | Once a week | 2–4 times a week | 5–6 times a week | Once a day | 2 times or more a day |
|---|---|---|---|---|---|---|---|
| Dark bread (rye bread, rye crispbread, whole grain bread) | ◯ | ◯ | ◯ | ◯ | ◯ | ◯ | ◯ |
| White bread (leavened bread, French bread etc.) | ◯ | ◯ | ◯ | ◯ | ◯ | ◯ | ◯ |
| Sweets, chocolate | ◯ | ◯ | ◯ | ◯ | ◯ | ◯ | ◯ |
| Sweet pastries (cookies, doughnuts, other pastries) | ◯ | ◯ | ◯ | ◯ | ◯ | ◯ | ◯ |
| Fresh vegetables or green salad | ◯ | ◯ | ◯ | ◯ | ◯ | ◯ | ◯ |
| Boiled vegetables | ◯ | ◯ | ◯ | ◯ | ◯ | ◯ | ◯ |
| Vegetable dishes | ◯ | ◯ | ◯ | ◯ | ◯ | ◯ | ◯ |
| Fruit | ◯ | ◯ | ◯ | ◯ | ◯ | ◯ | ◯ |
| Berries | ◯ | ◯ | ◯ | ◯ | ◯ | ◯ | ◯ |
| 100 % juice | ◯ | ◯ | ◯ | ◯ | ◯ | ◯ | ◯ |
| Fish | ◯ | ◯ | ◯ | ◯ | ◯ | ◯ | ◯ |
| Poultry | ◯ | ◯ | ◯ | ◯ | ◯ | ◯ | ◯ |
| Meat or processed meats (e.g. sausages) | ◯ | ◯ | ◯ | ◯ | ◯ | ◯ | ◯ |
| Skimmed milk or fat-free dairy products | ◯ | ◯ | ◯ | ◯ | ◯ | ◯ | ◯ |

Sleep sufficiency

**52. Do you feel that you get enough sleep?**

- ◯ yes, almost always
- ◯ yes, often
- ◯ rarely or hardly ever

Alcohol use

**31. How much, on average, do you consume the following alcoholic beverage?**

**a. Beer or cider**

- ◯ none
- ◯ less than one bottle a week
- ◯ 1–4 bottles a week
- ◯ 5–12 bottles a week
- ◯ 13–24 bottles a week
- ◯ 25–47 bottles a week
- ◯ 48 bottles or more a week

**b. Wine or equivalent alcoholic beverage**

- ◯ none
- ◯ less than a glass a week
- ◯ 1–4 glasses a week
- ◯ 1–2.5 bottles a week
- ◯ 3–4.5 bottles a week
- ◯ 5–9 bottles a week
- ◯ 10 bottles or more a week

**c. Spirits**

- ◯ not at all
- ◯ less than half a bottle a month
- ◯ 0.5–1.5 bottles a month
- ◯ 2–3.5 bottles a month
- ◯ 4–9 bottles a month
- ◯ 10–19 bottles a month
- ◯ 20 bottles or more a month

**32. The next question concerns situations in which you drink six or more servings of alcoholic beverages at one sitting. Six or more servings is equivalent to at least:**

**- 4 pints (0.5 l each) medium-strength beer/mild cider or**
**- 3 pints (0.5 l each) strong beer/strong cider or**
**- one bottle (0.75 l) of mild wine (12%) or**
**- 6 restaurant servings (4 cl each) of spirits**

**How often do you drink six or more servings of alcoholic beverages at one sitting?**

- ◯ never
- ◯ less than once a month
- ◯ once a month
- ◯ once a week
- ◯ a few times a week
- ◯ every day or almost every day

Smoking

**28 a. Do you smoke cigarettes?**

- ◯ yes, every day, how many cigarettes a day? ______
- ◯ occasionally
- ◯ not anymore - I quit smoking in (year) ______
- ◯ I have never smoked

**28 b. Do you use snuff?**

- ◯ yes, every day
- ◯ occasionally
- ◯ not anymore
- ◯ I have never used snuff

**28 c. Do you use electronic cigarettes (vape)?**

- ◯ yes, every day
- ◯ occasionally
- ◯ not anymore
- ◯ I have never used an electronic cigarette
