# Supplementary material for: Latent classes of unhealthy behaviours and their associations with subsequent sickness absence: a prospective register-linkage study among Finnish young and early midlife employees
Source: BMJ Open. 2023 May 11;13(5):e070883. doi: 10.1136/bmjopen-2022-070883 (PMC10186467; doi:10.1136/bmjopen-2022-070883)
Supplement: Author's manuscript [file bmjopen-2022-070883.draft_revisions.pdf]

BMJ Open

BMJ Open is committed to open peer review. As part of this commitment we make the peer review history of every article we publish publicly available.

When an article is published we post the peer reviewers' comments and the authors' responses online. We also post the versions of the paper that were used during peer review. These are the versions that the peer review comments apply to.

The versions of the paper that follow are the versions that were submitted during the peer review process. They are not the versions of record or the final published versions. They should not be cited or distributed as the published version of this manuscript.

BMJ Open is an open access journal and the full, final, typeset and author-corrected version of record of the manuscript is available on our site with no access controls, subscription charges or pay-per-view fees (http://bmjopen.bmj.com).

If you have any questions on BMJ Open's open peer review process please email info.bmjopen@bmj.com

BMJ Open

Unhealthy behaviours and their associations with subsequent sickness absence among young and early midlife municipal employees: a latent class analysis with prospective register-linkage

Journal:	*BMJ Open*
Manuscript ID	bmjopen-2022-070883
Article Type:	Original research
Date Submitted by the Author:	08-Dec-2022
Complete List of Authors:	Salmela, Jatta; University of Helsinki Faculty of Medicine, Department of Public Health<br>Lahti, Jouni; University of Helsinki Faculty of Medicine, Department of Public Health<br>Kanerva, Noora; University of Helsinki Faculty of Agriculture and Forestry, Department of Food and Nutrition<br>Rahkonen, Ossi; University of Helsinki Faculty of Medicine, Department of Public Health<br>Kouvonen, Anne; Helsingin Yliopisto, Faculty of Social Sciences; Queen's University Belfast,<br>Lallukka, Tea; University of Helsinki Faculty of Medicine, Department of Public Health
Keywords:	PREVENTIVE MEDICINE, EPIDEMIOLOGIC STUDIES, OCCUPATIONAL & INDUSTRIAL MEDICINE, PUBLIC HEALTH

SCHOLARONE™
Manuscripts

I, the Submitting Author has the right to grant and does grant on behalf of all authors of the Work (as defined in the below author licence), an exclusive licence and/or a non-exclusive licence for contributions from authors who are: i) UK Crown employees; ii) where BMJ has agreed a CC-BY licence shall apply, and/or iii) in accordance with the terms applicable for US Federal Government officers or employees acting as part of their official duties; on a worldwide, perpetual, irrevocable, royalty-free basis to BMJ Publishing Group Ltd ("BMJ") its licensees and where the relevant Journal is co-owned by BMJ to the co-owners of the Journal, to publish the Work in this journal and any other BMJ products and to exploit all rights, as set out in our licence.

The Submitting Author accepts and understands that any supply made under these terms is made by BMJ to the Submitting Author unless you are acting as an employee on behalf of your employer or a postgraduate student of an affiliated institution which is paying any applicable article publishing charge ("APC") for Open Access articles. Where the Submitting Author wishes to make the Work available on an Open Access basis (and intends to pay the relevant APC), the terms of reuse of such Open Access shall be governed by a Creative Commons licence – details of these licences and which Creative Commons licence will apply to this Work are set out in our licence referred to above.

Other than as permitted in any relevant BMJ Author's Self Archiving Policies, I confirm this Work has not been accepted for publication elsewhere, is not being considered for publication elsewhere and does not duplicate material already published. I confirm all authors consent to publication of this Work and authorise the granting of this licence.

For peer review only

TITLE PAGE

Title of the article:

Unhealthy behaviours and their associations with subsequent sickness absence among young and early midlife municipal employees: a latent class analysis with prospective register-linkage

Authors:

Jatta Salmela [1]*, Jouni Lahti [1], Noora Kanerva [2], Ossi Rahkonen [1], Anne Kouvonen [3,4], Tea Lallukka [1]

Affiliations:

[1] Department of Public Health, University of Helsinki, Helsinki, Finland

[2] Department of Food and Nutrition, University of Helsinki, Helsinki, Finland

[3] Faculty of Social Sciences, University of Helsinki, Helsinki, Finland

[4] Centre for Public Health, Queen's University Belfast, Belfast, Northern Ireland

***Corresponding author:**

Jatta Salmela, PhD

Postal address:

Department of Public Health

PO BOX 20 (Tukholmankatu 8 B)

00014 University of Helsinki

Finland

email: jatta.salmela@helsinki.fi

Word count, excluding title page, abstract, references, figures, and tables:

3492

Keywords:

Employment, Health Behavior, Latent Class Analysis, Sick Leave

For peer review only

ABSTRACT

Objectives: Unhealthy behaviours are associated with increased sickness absence (SA), but few studies have considered person-oriented approach in these associations. Using latent class analysis, we examined clustering of unhealthy behaviours among Finnish municipal employees and their associations with subsequent SA.

Design: A prospective register-linkage study.

Setting: Unhealthy behaviours (low leisure-time physical activity, non-daily fruit and vegetable consumption, insufficient sleep, excessive alcohol use, and tobacco use) were derived from the Helsinki Health Study questionnaire survey, collected in 2017 among 19–39-year-old employees of the City of Helsinki, Finland.

Participants: A total of 4002 employees (81% women) of the City of Helsinki, Finland.

Primary outcome measures: The questionnaire data were prospectively linked to employer's SA register through March 2020. Associations between latent classes of unhealthy behaviours and subsequent SA (1–7 days / 8+ days / all lengths) were examined using negative binomial regression.

Results: Among women, a 3-class latent class model was selected: 1) few unhealthy behaviours (84%), 2) excessive alcohol and tobacco use (12%), and 3) several unhealthy behaviours (5%). Women belonging to Classes 2 and 3 had increased SA rates compared to those in Class 1, regardless of the length of SA spells. Among men, a 2-latent class model was selected: 1) few unhealthy behaviours (53%) and 2) several unhealthy behaviours (47%). Men belonging to Class 2 had increased rates of 1–7 days' SA compared to men in Class 1.

Conclusions: This study suggests that preventive actions aiming to reduce employees' SA should consider simultaneously several unhealthy behaviours. Targeted interventions may benefit of identifying the clustering of these behaviours among occupational groups.

STRENGTHS AND LIMITATIONS OF THIS STUDY

- Unhealthy behaviours have been associated with increased rates of sickness absence, but few studies have utilised person-oriented approach to examine these associations.
- This study used latent class analysis to identify clustering of unhealthy behaviours among young and midlife Finnish municipal employees.
- Prospective register-linkage enabled us to examine how clustering of unhealthy behaviours was associated with subsequent sickness absence (1–7 days / 8+ days / all lengths), with a mean follow-up time of 2.13 years.
- Although the large proportion of women well represents the gender distribution in the target population and in the municipal sector in Finland in general, the small number of men limits the interpretation of the findings among men and the gender comparisons.

For peer review only

INTRODUCTION

Health behaviours have a major contribution to employees' sickness absence (SA). It is estimated that 15–31% of SA could be attributed to unhealthy behaviours(1,2). In addition to their independent contributions, health behaviours can mediate the effects of working conditions and socioeconomic circumstances on SA(3,4). In Finland, as in most high-income countries, the leading causes for medically certified SA are mental and musculoskeletal disorders(5,6). Unhealthy behaviours, such as low physical activity, poor sleep, binge drinking, and smoking, have been associated with medically certified but also self-certified SA(1,7–9). However, the results are not fully consistent(2,10). Diet appears to have a minor contribution to SA(2,3,7,11), but since obesity is consistently associated with SA(12), dietary aspects—which play a key role in weight management—should not be neglected.

Accumulation of several unhealthy behaviours have been shown to increase SA more than individual unhealthy behaviours(2,7,13). Our previous study on midlife and ageing Finnish employees found that the joint contribution of physical inactivity and smoking was especially detrimental for employer's cost of 1–14 days' SA(13). Health behaviours tend to be clustered within population groups(14,15), and these clusters may have synergistic effects on health(14). Considering clustering of unhealthy behaviours can help policymakers and researchers to design targeted interventions to improve employees' health behaviours and reduce SA. However, to best of our knowledge, no studies have examined how clustering of unhealthy behaviours is associated with SA. Clustering techniques, such as latent class analyses, can provide more holistic approach on how health behaviours contribute to SA compared to summary indices(2,7) that consider each risk factor equally and disregard their interconnections(16).

This study aimed to identify latent classes of five unhealthy behaviours among 19–39-year-old employees of the City of Helsinki, Finland. Furthermore, using linkage to employer's SA register, we aimed to examine the associations between the latent classes with subsequent SA.

METHODS

Data and study population

This study is a part of the Helsinki Health Study of young and early midlife employees of the City of Helsinki(17). The target population included 11,459 employees who were born in 1978 or later, who had a job contract of at least 50% of regular work hours per week, and whose employment contract had lasted at least 4 months before the data collection began in autumn 2017. Data were collected via online and mailed questionnaires, which included a large variety of questions related to participants' social and economic characteristics and health behaviours. Additionally, shorter telephone interviews were conducted to target those who did not respond online or via mail. The overall response rate was 51.5% (n=5898)(17). The survey data were linked to employer's personnel register data for those who gave their written informed consent (82% of respondents, n=4864). We excluded telephone interviewees (n=651) since the interviews did not include all

For peer review only

the variables of interest in this study, as well as participants who had missing data on working time or on all health behaviours of interest (n=34), or who had extreme values in health behaviours (n=177) (**Figure S1**). The final analytical sample included 4002 participants (81% women).

Health behaviour measures

We included five unhealthy behaviours from the survey: 1) low leisure-time physical activity (LTPA), 2) non-daily fruit and vegetable (F&V) consumption, 3) insufficient sleep, 4) excessive alcohol use, and 5) tobacco use. Since it is not computationally possible to include too many multi-categorical variables or variables with very small group sizes in the LCA models, we dichotomised all health behaviour measures taking into consideration current guidelines and group sizes in the variables. Participants were inquired about their weekly volume and intensity of exercise in their leisure time or while commuting during the past 12 months. Four levels of intensity were provided, and they were multiplied by the time used per week in LTPA, yielding weekly metabolic equivalent task (MET) -hours(7). Then, we dichotomised participants to those with high/moderate LTPA and those with low LTPA by using a cut-point of 20 MET-hours. Twenty MET-hours equals, for instance, 2.5 hours brisk walking and 1.5 hours walking, which was considered closely to correspond current guidelines(18,19).

F&V consumption during the past 4 weeks was inquired using a 14-item food frequency questionnaire. We dichotomised participants into daily (once a day or more F or V) and non-daily F&V consumers. Subjective experience of sleep was used as a sleep measure. We dichotomised participants into those who estimated that they sleep always/often sufficiently and those who estimated that they sleep seldom/never sufficiently. Alcohol use combined the measures of total weekly alcohol use and binge drinking behaviour. Weekly alcohol use was calculated based on participants' estimation on how often they consume different alcohol types (beer/cider, wine, and spirits). Seven frequency alternatives were provided for each question, with one unit of alcohol equalling 12g ethanol. Based on the Finnish recommendations(20), 7 weekly units for women and 14 weekly units for men were considered as cut-points. Additionally, participants were asked how often they drink six units or more at once (six response alternatives). We dichotomised those drinking less than 7/14 (women/men) units per week and binge drinking less than once a month into moderate alcohol users, and others to excessive alcohol users. Participants were provided four alternatives to estimate their use of tobacco products (cigarettes, e-cigarettes, and snus): 'yes, daily', 'sporadically', 'not nowadays', and 'never'. We dichotomised participants into never-/ex-users, and those using daily/occasionally tobacco products.

Sickness absence measures

The data on SA were derived from the personnel register of the City of Helsinki. The follow-up of SA began from one day after receiving the completed survey questionnaire and continued until 31st March 2020 or until the end of one's employment contract, whichever came first. The time limit was selected so that we could exclude the potential influence of the COVID-19 pandemic to the results. The mean follow-up time was 2.13

For peer review only

years. We combined overlapping and consecutive SA spells and divided them into SA spells of 1–7 days and 8+ days. During the follow-up, the City of Helsinki had a policy that 1–7 days' SA could be given to an employee by their supervisor, nurse, occupational physiotherapist, or physician, whereas 8+ days' SA required a medical certification approved by a physician. The policy was the same for all employees. Additionally, we analysed all lengths' SA.

Covariates

We stratified all analyses by gender (woman/man), given that notable gender differences have been observed in SA and health behaviours(21,22), and clustering of health behaviours may vary by gender(15). Age included categories of 19–29, 30–34, and 35–39 years. Marital status was derived from the questionnaire and was dichotomised into married/cohabiting and other. In the questionnaire, participants were inquired whether they had any 0–18-year-old children living in their household ('yes/no'). Occupational class was derived from the employer's personnel register for those who gave their informed consent for register linkage (82%), and for others, the information was derived from the questionnaire. Occupational class included four groups: managers and professionals, semi-professionals, routine non-manual workers, and manual workers. It is noteworthy that in recent years the City of Helsinki has outsourced most of their manual work (e.g., cleaning and transport work), and therefore the proportion of manual workers employed by the city is now very low. Prior SA, especially past year's SA, is known to predict future SA(10,23). Thus, we included prior SA of any lengths during one year before participant's response to the questionnaire.

Statistical methods

We first tabulated descriptive statistics by key exposure variables. Then, SA days per 10 person-years were calculated by individual health behaviours. We identified latent classes of unhealthy behaviours using latent class analysis (LCA). LCA is a person-oriented statistical procedure to detect latent (unobserved) subgroups, which share certain outward characteristics, within a heterogeneous population(24,25). This subtype of structural equation modelling uses categorical indicator variables to form latent classes based on the indicator variables. Participants are assigned to the latent classes based on their probability of class membership. We used the following statistical criteria for selecting the most optimal number of latent classes: Bayesian information criterion (BIC), Akaike information criterion (AIC), average posterior probabilities of class membership (>0.8), class sizes (>50 cases or >5% of the sample), and entropy (>0.8)(25). One- to five-class models were run, and the model fit evaluation process is shown in **Table S1**. Additionally, we considered the interpretability of the models to select the final models(25).

We used negative binomial regression to examine associations between latent classes of unhealthy behaviours and subsequent SA due to overdispersion in the data. Rate ratios (RRs) and predictive margins with 95% confidence intervals (CIs) were calculated. Model 1 was adjusted for age, and model 2 further for marital status, children living in the household, occupational class, and prior SA. Natural logarithm of the follow-up

For peer review only

time was included as an offset variable in all models to consider differences in the follow-up times between participants. All analyses were performed using STATA version 17.0 (StataCorp LLC, College Station, TX, USA).

Patient and public involvement

Patients or the public were not involved in this study.

RESULTS

Characteristics of study population

Most participants had at least one unhealthy behaviour (67% of women and 83% of men), whereas under 1% of women and men had all five unhealthy behaviours. Low LTPA and insufficient sleep were equally common among women and men (**Table 1**). However, non-daily F&V consumption, excessive alcohol use, and tobacco use were more common among men than among women. Most women and men were married/cohabiting and around 40% had children living in their household. Only 3% of women were manual workers while the corresponding proportion for men was 13%.

Table 1. Characteristics of the participants by sociodemographic factors and health behaviours among women and men.

**Total**	**Women (n, %)**	**Men (n, %)**
	3228 (80.7)	774 (19.3)
Health behaviours		
**Leisure-time physical activity [a]**		
High or moderate activity	2689 (84.4)	651 (85.3)
Low activity	499 (15.7)	112 (14.7)
**Fruit and vegetable consumption**		
Daily	2595 (80.5)	463 (60.0)
Non-daily	629 (19.5)	309 (40.0)
**Sleep sufficiency**		
Mostly sufficient sleep	2146 (66.9)	521 (67.8)
Insufficient sleep	1064 (33.2)	248 (32.3)
**Alcohol use [b]**		
Moderate	2492 (79.9)	423 (55.8)
Excessive	626 (20.1)	335 (44.2)
**Tobacco use [c]**		
No	2430 (75.8)	471 (61.1)
Currently or occasionally	777 (24.2)	300 (38.9)
Sociodemographic factors		
**Age**		
19–29 years	1049 (32.5)	197 (25.5)
30–34 years	1108 (34.3)	252 (32.6)
35–39 years	1071 (33.2)	325 (42.0)
**Marital status**		
Married or cohabiting	2122 (65.7)	570 (73.6)
Other	1106 (34.3)	204 (26.4)
**Children living in the household**		
No	1851 (57.3)	467 (60.3)

For peer review only

Yes	1377 (42.7)	307 (39.7)
**Occupational class**		
Managers and professionals	895 (27.7)	241 (31.1)
Semi-professionals	1402 (43.4)	242 (31.3)
Routine non-manual workers	843 (26.1)	191 (24.7)
Manual workers	88 (2.7)	100 (12.9)

[a] Leisure-time physical activity (LTPA) included physical activity during leisure time and active commuting. High or moderate LTPA was considered as ≥20 metabolic equivalent task (MET) -hours per week and low LTPA as <20 MET-hours per week.

[b] Moderate alcohol use: ≤7 units of alcohol per month and binge drinking less than once a month among women, and ≤14 units of alcohol per month and binge drinking less than once a month among men. Excessive alcohol use: >7 units of alcohol per month and binge drinking less than once a month among women, and >14 units of alcohol per month and binge drinking less than once a month among men.

[c] Tobacco use included use of cigarettes, e-cigarettes, and snus.

During the follow-up, we recorded altogether 117 SA days/10 person-years for women and 93 SA days/10 person-years for men. Of women, 15% had no 1–7 days' SA and 69% had no 8+ days' SA during the follow-up. For men, the corresponding figures were 18% and 75%. Participants with healthier behaviours had less SA than those with unhealthier behaviours in general (**Table 2**). However, F&V consumption and alcohol use were exceptions among men in terms of 8+ days' SA: those with healthier behaviour had more or equally 8+ days' SA compared to those with unhealthier behaviour. When scrutinising all lengths' SA, the greatest differences between healthy and unhealthy behaviour groups were seen in tobacco use among women and in sleep among men.

For peer review only

Table 2. Mean sickness absence days per 10 person-years with 95% confidence intervals (in parenthesis), by health behaviours among women and men.

Health behaviours	Women (n=3228)			Men (n=774)		
	1–7 days' SA	8+ days' SA	All lengths' SA	1–7 days' SA	8+ days' SA	All lengths' SA
**Leisure-time physical activity [a]**						
High or moderate activity	63 (60-66)	68 (54-81)	130 (116-144)	50 (45-54)	40 (30-51)	90 (78-102)
Low activity	73 (66-80)	75 (56-94)	148 (127-169)	65 (52-79)	52 (16-87)	117 (76-158)
**Fruit and vegetable consumption**						
Daily	62 (59-65)	68 (54-81)	130 (115-144)	47 (42-52)	45 (31-59)	92 (77-108)
Non-daily	74 (67-81)	74 (55-93)	148 (126-170)	59 (52-67)	36 (22-51)	96 (78-113)
**Sleep sufficiency**						
Mostly sufficient sleep	62 (59-65)	60 (45-75)	122 (106-137)	46 (41-50)	32 (23-41)	78 (66-89)
Insufficient sleep	69 (64-74)	88 (70-107)	157 (136-178)	65 (56-73)	62 (37-87)	127 (99-154)
**Alcohol use [b]**						
Moderate	61 (58-64)	66 (53-80)	127 (113-142)	49 (43-54)	42 (28-56)	91 (75-107)
Excessive	77 (69-84)	81 (58-104)	157 (131-183)	56 (50-63)	42 (27-58)	99 (80-117)
**Tobacco use [c]**						
No	59 (56-62)	62 (48-76)	121 (106-136)	47 (42-52)	37 (25-49)	84 (70-98)
Currently or occasionally	81 (74-87)	92 (73-111)	173 (151-194)	59 (51-66)	49 (32-67)	108 (88-129)

[a] Leisure-time physical activity (LTPA) included physical activity during leisure time and active commuting. High or moderate LTPA was considered as ≥20 metabolic equivalent task (MET) -hours per week and low LTPA as <20 MET-hours per week.

[b] Moderate alcohol use: ≤7 units of alcohol per month and binge drinking less than once a month among women, and ≤14 units of alcohol per month and binge drinking less than once a month among men. Excessive alcohol use: >7 units of alcohol per month and binge drinking less than once a month among women, and >14 units of alcohol per month and binge drinking less than once a month among men.

[c] Tobacco use included use of cigarettes, e-cigarettes, and snus.

For peer review only

Latent classes of unhealthy behaviours

The most optimal number of latent classes of unhealthy behaviours was three for women and two for men (**Figure 1, Table S1**). Although model fit statistics preferred the 2-class solution for women, 3 classes were selected as they were interpretatively reasonable and provided new information about the data. Most statistical criteria preferred the 3-class solution for men, but we selected 2 classes due to one too small class size (n=39) in the 3-class solution. Marginal means for each unhealthy behaviour within latent classes are shown in **Table S2**.

[Figure 1 here]

Of women, 84% had the highest posterior probability for belonging to Class 1, and for Classes 2 and 3, the corresponding proportions were 12% and 5% (**Figure 1a**). Class 1 was characterised by overall low probabilities of having unhealthy behaviours. Class 2 was characterised especially by excessive alcohol use and tobacco use, whereas probabilities for other unhealthy behaviours were somewhat low. In Class 3, there were increased probabilities for all other unhealthy behaviours except excessive alcohol use. Of men, 53% had the highest posterior probability for belonging to Class 1, and 47% to Class 2 (**Figure 1b**). Class 1 was characterised by somewhat low probabilities of having any unhealthy behaviours. The probabilities of having unhealthy behaviours were overall increased in Class 2, and it was especially characterised by low LTPA, non-daily F&V consumption, and excessive alcohol use.

For peer review only

Associations between latent classes of unhealthy behaviours and sickness absence

Women belonging to Classes 2 and 3 had increased SA rates compared to Class 1 (**Table 3**). However, the associations with 8+ days' SA were not statistically significant. Women belonging to Classes 2 and 3 had increased rates of 1–7 days' SA even after adjustment for age, marital status, children living in the household, occupational class, and prior SA (**Table 3, M2**). Men belonging to Class 2 had increased SA rates compared to Class 1 (**Table 4**). However, statistically significant association was found only for 1–7 days' SA in the age-adjusted model (**M1, Table 4**). This association attenuated after further adjustments (**M2, Table 4**), especially after adjustment for occupational class.

Table 3. Associations between latent classes of unhealthy behaviours and sickness absence (SA) among women. Rate ratios (RR) and predictive margins with 95% confidence intervals (CI) from negative binomial regression models [a] are shown.

	1–7 days' SA		8+ days' SA		All lengths' SA	
**Latent class**	**M1: Adjusted for age**	**M2: M1 + marital status, children living in the household, occupational class, prior SA [b]**	**M1: Adjusted for age**	**M2: M1 + marital status, children living in the household, occupational class, prior SA [b]**	**M1: Adjusted for age**	**M2: M1 + marital status, children living in the household, occupational class, prior SA [b]**

	**RR (95 % CI)**					
**Class 1**	ref.	ref.	ref.	ref.	ref.	ref.
**Class 2**	1.39 (1.24-1.57)	1.21 (1.08-1.36)	1.37 (0.92-2.02)	1.35 (0.92-1.97)	1.39 (1.20-1.61)	1.29 (1.13-1.48)
**Class 3**	1.37 (1.14-1.64)	1.19 (1.00-1.42)	1.31 (0.72-2.38)	1.22 (0.68-2.18)	1.34 (1.07-1.67)	1.18 (0.96-1.46)
	**Predictive margins (95 % CI)**					
**Class 1**	12.6 (12.1-13.2)	12.2 (11.7-12.7)	13.7 (11.8-15.6)	10.8 (9.3-12.2)	26.2 (24.8-27.5)	23.0 (21.9-24.1)
**Class 2**	17.6 (15.6-19.6)	14.8 (13.2-16.4)	18.7 (11.8-25.5)	14.5 (9.3-19.7)	36.5 (31.5-41.5)	29.7 (25.9-33.5)
**Class 3**	17.3 (14.2-20.3)	14.5 (12.1-17.0)	17.9 (7.4-28.3)	13.1 (5.7-20.5)	35.0 (27.4-42.6)	27.2 (21.7-32.7)

[a] Natural logarithm of the follow-up time is included in the models as an offset variable.

[b] Prior sickness absence of all lengths one year before the follow-up, divided by the working time in years during the one year's period.

Table 4. Associations between latent classes of unhealthy behaviours and sickness absence (SA) among men. Rate ratios (RR) and predictive margins with 95% confidence intervals (CI) from negative binomial regression models [a] are shown.

	**1–7 SA days**		**8+ SA days**		**SA days of all lengths**	
**Latent class**	**M1: Adjusted for age**	**M2: M1 + marital status, children living in the household, occupational class, prior SA [b]**	**M1: Adjusted for age**	**M2: M1 + marital status, children living in the household, occupational class, prior SA [b]**	**M1: Adjusted for age**	**M2: M1 + marital status, children living in the household, occupational class, prior SA [b]**
	**RR (95 % CI)**					
**Class 1**	ref.	ref.	ref.	ref.	ref.	ref.
**Class 2**	1.23 (1.04-1.45)	1.11 (0.95-1.31)	1.16 (0.65-2.06)	1.01 (0.58-1.77)	1.20 (0.98-1.46)	1.06 (0.88-1.28)
	**Predictive margins (95 % CI)**					
**Class 1**	10.0 (8.8-11.1)	9.5 (8.5-10.6)	8.2 (5.0-11.4)	6.3 (4.0-8.7)	18.2 (15.7-20.7)	16.2 (14.2-18.2)
**Class 2**	12.2 (10.8-13.7)	10.6 (9.4-11.8)	9.6 (5.6-13.5)	6.4 (3.8-8.9)	21.8 (18.7-25.0)	17.2 (14.9-19.5)

[a] Natural logarithm of the follow-up time is included in the models as an offset variable.

[b] Prior sickness absence of all lengths one year before the follow-up, divided by the working time in years during the one year's period.

DISCUSSION

Summary of the main findings

By using the LCA method, we selected three latent classes of unhealthy behaviours among women, characterised as follows: 1) few unhealthy behaviours, 2) excessive alcohol use and tobacco use, and 3) several unhealthy behaviours. Among men, we selected two latent classes with the following characteristics: 1) few unhealthy behaviours and 2) several unhealthy behaviours. Women in Classes 2 and 3, and men in Class 2 had increased rates of 1–7 days' SA compared to Class 1. The associations between latent classes of unhealthy behaviours and 8+ days' SA were not statistically significant either among women or men.

For peer review only

Comparisons to the previous literature

The majority of women and men were most likely to belong to Class 1, characterised by overall healthier behaviours. Similarly, a systematic review of the clustering of smoking, nutrition, alcohol, and physical activity in adults found that a majority of included studies reported a 'healthy' cluster, characterised by the absence of any unhealthy behaviours(14). This was not affected by in how health behaviours were defined or by the used clustering analysis method(14). Some more recent studies have also identified a class of overall healthier behaviours(26–28). Additionally, previous studies have found especially alcohol consumption and smoking often clustering(14,15), which we also observed in women in Class 2. However, in men, this was not observed with two latent classes. Further analyses revealed that with a 3-class solution in men, clustering of excessive alcohol use and tobacco use existed similarly as in women. Clustering of low LTPA and non-daily F&V consumption, which we observed in Class 2 among men, has been found in many of the previous studies (14,15). However, Noble's et al. (2015) systematic review did not find clustering of physical inactivity, poor diet, and excess alcohol use—the combination that we found to reflect Class 2 in men—in any of the included studies(14). Finally, clustering of several unhealthy behaviours have been observed in many previous studies(14,26), which we also could observe in Class 3 among women and Class 2 among men.

To our knowledge, no previous studies have examined associations between latent classes of unhealthy behaviours and SA, although the relationship between health behaviours and SA are broadly studied in general. Concerning single unhealthy behaviours, previous studies have associated low LTPA(1,9,29,30), poor sleep(31,32), excessive alcohol use(1,33), and smoking(1,2,29,30,32,34) with SA, while the contribution of poor diet to SA has been modest(2,3,7,11,29). Although diet has not been associated with SA as strongly as other health behaviours, we found that inadequate F&V consumption was one major characteristic of Class 3 among women and Class 2 among men—the classes that were associated with increased subsequent SA. Our previous study on midlife and older employees also showed that the joint contribution of F&V consumption and LTPA to SA might be stronger than the individual contribution of LTPA(11). However, since F&V consumption reflects only partially participants' overall diet, further studies that consider dietary patterns more comprehensively are needed.

Our previous study showed that midlife and older employees with three or more unhealthy behaviours had higher cost of 1–14 days' SA than employees without any unhealthy behaviours(7). In particular, low LTPA, poor sleep, and current smoking increased the SA cost(7). Another study by our research group found that the joint contributions of low LTPA, poor sleep, and smoking to 1–14 days' SA cost were stronger than the contributions of these health behaviours individually(13). A Norwegian study on general working population found that an exposure to multiple health-related risk factors (low physical activity, unhealthy diet, obesity, and current smoking) was associated with increased subsequent 1–14 days' and 15+ days' SA(2). Additionally, a Danish study on private sector employees found that exposure to multiple health-related risk factors (dyssomnia, overweight, unhealthy food habits, smoking, excessive alcohol use, and low physical activity)

For peer review only

were related to increased 1–14 days' SA(32). These previous findings are concordant with our finding which indicated that the latent classes of several unhealthy behaviours (Class 3 for women and Class 2 for men) were related to increased SA rates.

We found that latent classes of unhealthy behaviours were associated with 1–7 days' SA among women and men, but not statistically significantly with 8+ days' SA. In contrast, previous studies have found stronger associations for longer SA spells(2,3). There is some evidence that younger employees have more often short-term SA and older employees long-term SA(34,35), which may partly explain our findings. Another explanation is that the follow-up period of 2.13 years may not be long enough to ensure the associations with 8+ days' SA since their rate during the follow-up was relatively low.

Previous research has shown that clustering of unhealthy behaviours is strongly related to socioeconomic position(14,15,26,27,36). Similarly, we found that managers and professionals were more likely to belong to the 'healthiest' latent classes (**Table S3**). However, occupational class together with other sociodemographic factors explained only some of the associations between the latent classes of unhealthy behaviours and SA. Since socioeconomic differences in SA are visible already among young employees(37) and employees in the lower socioeconomic positions are more likely to have adverse working conditions (e.g., higher exposure to physical workload) that are strongly related to increased SA(29,34,38,39), these factors should not be neglected when designing targeted health behaviour interventions at workplaces. Burdorf and Robroek (2018) have suggested that preventive interventions should simultaneously consider improvements in working conditions and health behaviours, and they should be targeted to high-risk and low-educated population groups(40). Additionally, given that younger age predisposes to clustering of unhealthy behaviours(**Table S3,**(14,26,27)—and that health behaviours may be more difficult to modify the older individuals are—preventive actions are especially needed among young employees in the lower socioeconomic positions.

Limitations and strengths

This study has a few limitations that should be considered. First, health behaviours were self-reported, thus biased estimates are possible. Second, the used cut-points in the health behaviour measures may have affected the identified latent classes. We tested various options and made the final decisions of the dichotomisations based on their consistency with the current guidelines and their proportions in the data. Third, the used cut-point in SA (1–7/8 days) complicates the comparisons to other studies since many previous studies have used cut-points of 3/4 days or 14/15 days to distinguish short-term SA from long-term SA. However, 15+ days' SA were rare in this study population, and the changes made in the SA practices by the City of Helsinki during the follow-up period supported using the chosen cut-point. SA spells of 8+ days were still relatively rare in the study population, and a longer follow-up period could have strengthened the interpretation of the findings. Fourth, the small number of men limits the interpretation of the findings among men and the gender

For peer review only

comparisons. The large proportion of women well represents, however, the gender distribution in the target population and in the municipal sector in Finland in general.

Fifth, missing data and non-participation may affect the findings. LCA uses maximum likelihood estimation and assumes missingness at random(25), thus missing data on health behaviours were allowed. However, we have carefully examined the representativeness of the data and found them to satisfactorily represent the target population (N=11,459)(17). The response rate to the survey was moderate (51.5%), and the non-respondents were somewhat more often men, manual workers, had lower income, and had more 15+ days' SA(17). Additionally, the participants included in this current study were more often of higher occupational class (**Table S4**); thus, our results may be slightly conservative(26,36). However, the sensitivity analyses showed that the final analytical sample (n=4002) highly resembled the full sample (n=5898) in terms of health behaviours and socioeconomic characteristics (**Table S4**). In addition to the use of comprehensive survey data, a further strength of this study is that we could link the questionnaire survey to employer's SA registers, which is rarely possible. Furthermore, using the person-oriented LCA method to deepen our understanding on the associations between unhealthy behaviours and SA is a novel approach in this study area.

CONCLUSIONS

This study identified three latent classes of unhealthy behaviours for women and two for men. The 'healthiest' classes among women and men showed the lowest SA rates. The associations of the latent classes of unhealthy behaviours were stronger with 1–7 days' than with 8+ days' SA. Thus, by considering the clustering of unhealthy behaviours among young and early midlife employees and intervening in them may reduce employees' short-term SA at least. Occupational class together with other sociodemographic factors explained some of the found associations, thus special focus on employees with lower occupational positions is needed.

DATA AVAILABILITY STATEMENT

Data are available upon reasonable request. The Helsinki Health Study survey data cannot be made publicly available due to strict data protection laws and regulations. The data can only be used for scientific research and to the research group's cooperation partners with a reasonable request and study plan. More information on the availability of the survey data can be inquired from the Helsinki Health Study research group (kttl-hhs@helsinki.fi). Register data cannot be shared.

ETHICS STATEMENTS

Patient consent for publication

Not applicable. All participants have been informed about their rights and other ethical considerations (e.g., no participant can be identified from published results, voluntary participation, possibility to withdraw from the study, and how and where the data are used) prior to their inclusion in the study.

For peer review only

Ethics approval

The Helsinki Health Study protocol has been approved by the ethics committees of Department of Public Health, University of Helsinki, and the health authorities of the City of Helsinki. The permission to have access to the employer's personnel register data was obtained from the City of Helsinki. Appropriate ethical aspects have been followed in all phases of the study, according to the Declaration of Helsinki.

ACKNOWLEDGEMENTS

The authors thank all participating employees and the personnel administration of the City of Helsinki.

FOOTNOTES

Twitter: @JattaSalmela, @HHS_helsinkiuni

Contributors: JS was the primary author of the paper, performed the statistical analyses, and is responsible for the overall content as guarantor. TL contributed to the study design. JL, NK, OR, AK, and TL contributed to the interpretation of the findings. JS, JL, NK, OR, AK, and TL critically reviewed the manuscript and approved the final version of the manuscript.

Funding: JS was supported by the Doctoral Programme in Population Health, University of Helsinki. JS and TL were supported by the Social Insurance Institution of Finland (grant 29/26/2020). OR was supported by the Juho Vainio Foundation. AK was supported by the Economic and Social Research Council (ESRC) (grant ES/S00744X/1).

Competing interests: None declared.

Patient and public involvement: Patients and/or the public were not involved in the design, or conduct, or reporting or dissemination plans of this research.

Provenance and peer review: Not commissioned; externally peer reviewed.

For peer review only

Supplemental material: This content has been supplied by the authors. It has not been vetted by BMJ Publishing Group Limited (BMJ) and may not have been peer-reviewed. Any opinions or recommendations discussed are solely those of the authors and are not endorsed by BMJ. BMJ disclaims all liability and responsibility arising from any reliance placed on the content. Where the content includes any translated material, BMJ does not warrant the accuracy and reliability of the translations (including but not limited to local regulations, clinical guidelines, terminology, drug names and drug dosages), and is not responsible for any error and/or omissions arising from translation and adaptation or otherwise.

REFERENCES

1. Virtanen M, Ervasti J, Head J, et al. Lifestyle factors and risk of sickness absence from work: a multicohort study. Lancet Public Health 2018;3(11):e545–54.

2. De Bortoli MM, Oellingrath IM, Fell AKM, et al. Influence of lifestyle risk factors on work ability and sick leave in a general working population in Norway: a 5-year longitudinal study. BMJ Open 2021;11(2):e045678.

3. Laaksonen M, Piha K, Martikainen P, et al. Health-related behaviours and sickness absence from work. Occup Environ Med 2009;66(12):840–7.

4. Robroek SJW, van Lenthe FJ, Burdorf A. The role of lifestyle, health, and work in educational inequalities in sick leave and productivity loss at work. Int Arch Occup Environ Health 2013;86(6):619–27.

5. The Social Insurance Institution of Finland. Statistical Yearbook of the Social Insurance Institution 2020. Helsinki: The Social Insurance Institution of Finland 2020:181–95.

6. OECD. Sickness, Disability and Work: Breaking the Barriers: A Synthesis of Findings across OECD Countries [online]. https://www.oecd.org/publications/sickness-disability-and-work-breaking-the-barriers-9789264088856-en.htm (accessed 21 Nov 2022).

7. Kanerva N, Pietiläinen O, Lallukka T, et al. Unhealthy lifestyle and sleep problems as risk factors for increased direct employers' cost of short-term sickness absence. Scand J Work Environ Health 2018;44(2):192–201.

8. Lallukka T, Haaramo P, Rahkonen O, et al. Joint associations of sleep duration and insomnia symptoms with subsequent sickness absence: the Helsinki Health Study. Scand J Public Health 2013;41(5):516–23.

9. Amlani NM, Munir F. Does Physical Activity Have an Impact on Sickness Absence? A Review. Sports Med 2014;44(7):887–907.

10. Gohar B, Larivière M, Lightfoot N, et al. Demographic, Lifestyle, and Physical Health Predictors of Sickness Absenteeism in Nursing: A Meta-Analysis. Saf Health Work 2021;12(4):536–43.

11. Salmela J, Lahti J, Mauramo E, et al. Associations of changes in diet and leisure-time physical activity with employer's direct cost of short-term sickness absence. Eur J Sport Sci 2020;20(2):240–8.

12. Duijvenbode DC van, Hoozemans MJM, Poppel MNM van, et al. The relationship between overweight and obesity, and sick leave: a systematic review. Int J Obes 2009;33(8):807–16.

For peer review only

13. Kanerva N, Lallukka T, Rahkonen O, et al. The joint contribution of physical activity, insomnia symptoms, and smoking to the cost of short-term sickness absence. Scand J Med Sci Sports 2019;29(3):440–9.

14. Noble N, Paul C, Turon H, et al. Which modifiable health risk behaviours are related? A systematic review of the clustering of Smoking, Nutrition, Alcohol and Physical activity ('SNAP') health risk factors. Prev Med 2015;81:16–41.

15. Meader N, King K, Moe-Byrne T, et al. A systematic review on the clustering and co-occurrence of multiple risk behaviours. BMC Public Health 2016;16:657.

16. McAloney K, Graham H, Law C, et al. A scoping review of statistical approaches to the analysis of multiple health-related behaviours. Prev Med 2013;56(6):365–71.

17. Lallukka T, Pietiläinen O, Jäppinen S, et al. Factors associated with health survey response among young employees: a register-based study using online, mailed and telephone interview data collection methods. BMC Public Health 2020;20(1):184.

18. UKK Institute. Aikuisten liikkumisen suositus [online, in Finnish]. https://ukkinstituutti.fi/liikkuminen/liikkumisen-suositukset/aikuisten-liikkumisen-suositus/ (accessed 21 Nov 2022).

19. Office of Disease Prevention and Health Promotion. Physical Activity Guidelines for Americans [online]. https://health.gov/our-work/nutrition-physical-activity/physical-activity-guidelines (accessed 21 Nov 2022).

20. Finnish Food Authority. Nutrition and food recommendations [online]. https://www.ruokavirasto.fi/en/themes/healthy-diet/nutrition-and-food-recommendations/ (accessed 21 Nov 2022).

21. Laaksonen M, Mastekaasa A, Martikainen P, et al. Gender differences in sickness absence--the contribution of occupation and workplace. Scand J Work Environ Health 2010;36(5):394–403.

22. Koponen P, Borodulin K, Lundqvist A, et al (eds.). Health, functional capacity and welfare in Finland – FinHealth 2017 study. National Institute for Health and Welfare (THL). Helsinki 2018, p. 236.

23. Roelen CAM, Koopmans PC, Schreuder JAH, et al. The history of registered sickness absence predicts future sickness absence. Occup Med Oxf Engl 2011;61(2):96–101.

24. Hagenaars JA, McCutcheon AL. Applied Latent Class Analysis. Cambridge University Press 2002. p. 478.

25. Weller BE, Bowen NK, Faubert SJ. Latent Class Analysis: A Guide to Best Practice. J Black Psychol 2020;46(4):287–311.

26. Hobbs M, Duncan MJ, Collins P, et al. Clusters of health behaviours in Queensland adults are associated with different socio-demographic characteristics. J Public Health 2019;41(2):268–77.

27. Hutchesson MJ, Duncan MJ, Oftedal S, et al. Latent Class Analysis of Multiple Health Risk Behaviors among Australian University Students and Associations with Psychological Distress. Nutrients 2021;13(2):425.

28. Pettigrew S, Dana LM, McAleese A, et al. Brief Report: A Latent Class Analysis of Guideline Compliance Across Nine Health Behaviors. Int J Behav Med 2022;29(1):110–5.

For peer review only

29. Robroek SJW, van den Berg TIJ, Plat JF, et al. The role of obesity and lifestyle behaviours in a productive workforce. Occup Environ Med 2011;68(2):134–9.

30. Quist HG, Thomsen BL, Christensen U, et al. Influence of lifestyle factors on long-term sickness absence among female healthcare workers: a prospective cohort study. BMC Public Health 2014;14:1084.

31. Lallukka T, Kaikkonen R, Härkänen T, et al. Sleep and sickness absence: a nationally representative register-based follow-up study. Sleep 2014;37(9):1413–25.

32. Nielsen NBS, Gunnarsen L, Iburg KM. The impact of stress and lifestyle factors on short-term sickness absence in a large Danish industrial company. Scand J Public Health 2021;14034948211060816.

33. Kaila-Kangas L, Koskinen A, Leino-Arjas P, et al. Alcohol use and sickness absence due to all causes and mental- or musculoskeletal disorders: a nationally representative study. BMC Public Health. 2018;18(1):152.

34. Alavinia SM, van den Berg TI, van Duivenbooden C, et al. Impact of work-related factors, lifestyle, and work ability on sickness absence among Dutch construction workers. Scand J Work Environ Health 2009;35(5):325–33.

35. Sumanen H, Pietiläinen O, Lahti J, et al. Sickness absence among young employees: trends from 2002 to 2013. J Occup Health 2015;57(5):474–81.

36. Skalamera J, Hummer RA. Educational attainment and the clustering of health-related behavior among U.S. young adults. Prev Med 2016;84:83–9.

37. Sumanen H, Pietiläinen O, Lahti J, et al. Interrelationships between education, occupational class and income as determinants of sickness absence among young employees in 2002–2007 and 2008–2013. BMC Public Health 2015;15:332.

38. Mänty M, Kouvonen A, Nordquist H, et al. Physical working conditions and subsequent sickness absence: a record linkage follow-up study among 19–39-year-old municipal employees. Int Arch Occup Environ Health 2022;95(2):489–97.

39. Heinonen N, Lallukka T, Lahti J, et al. Working Conditions and Long-Term Sickness Absence Due to Mental Disorders. J Occup Environ Med 2022;64(2):105–14.

40. Burdorf A, Robroek S. Does lifestyle matter for sickness absence? Lancet Public Health. 2018;3(11):e513–4.

FIGURE LEGENDS

Figure 1. Latent classes of unhealthy behaviours among women (a) and men (b). F&V = fruit and vegetable, LTPA = leisure-time physical activity.

For peer review only

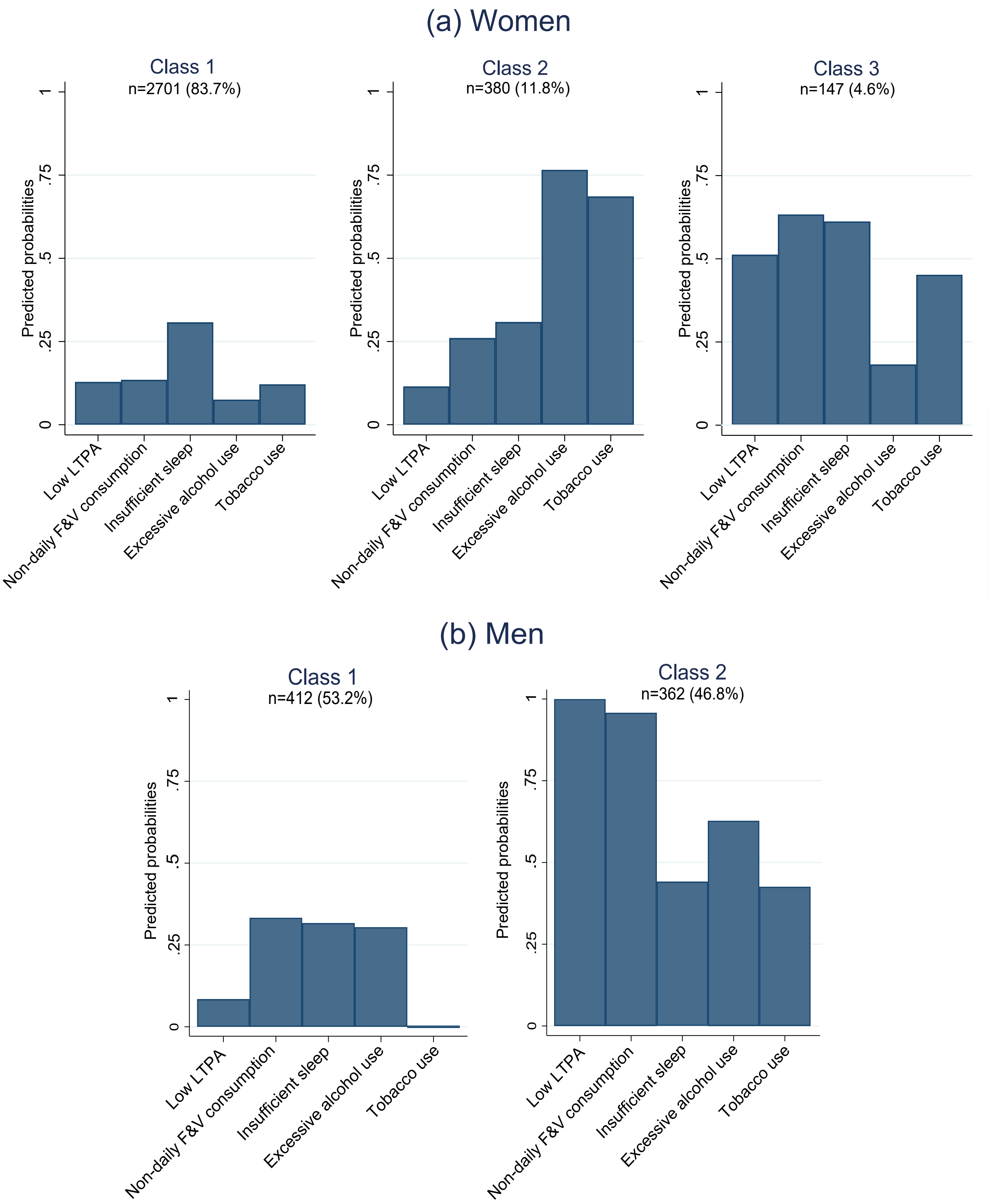
(a) Women
Class 1
n=2701 (83.7%)
Class 2
n=380 (11.8%)
Class 3
n=147 (4.6%)
Predicted probabilities
0
.25
.5
.75
1
Low LTPA
Non-daily F&V consumption
Insufficient sleep
Excessive alcohol use
Tobacco use
(b) Men
Class 1
n=412 (53.2%)
Class 2
n=362 (46.8%)

1 **SUPPLEMENTAL MATERIAL**

2

3 **Table S1.** Model fit statistics of latent classes of unhealthy behaviours among women and men.

Number of latent classes	Class membership based on posterior probabilities, n (%)	Marginal probabilities of class membership	Average posterior probabilities	Average posterior probabilities of class membership in each class	AIC value	BIC value	Entropy
**Women**							
1	3228 (100)	1.0	1.00	1.00	16715.2	16745.6	1.00
2	2281 (70.7)	0.65	0.87	0.87	16402.5	16469.4	0.86
	947 (29.3)	0.35		0.88			
3	2701 (83.7)	0.75	0.85	0.87	16366.4	16469.8	0.86
	147 (4.6)	0.08		0.63			
	380 (11.8)	0.17		0.82			
4	1881 (58.3)	0.57	0.81	0.87	16374.7	16508.5	0.83
	781 (24.2)	0.21		0.71			
	128 (4.0)	0.07		0.66			
	438 (13.6)	0.15		0.75			
5	1628 (50.4)	0.44	0.83	0.85	16371.3	16535.4	0.90
	846 (26.2)	0.24		0.82			
	199 (6.2)	0.10		0.60			
	497 (15.4)	0.21		0.89			
	58 (1.8)	0.02		0.67			
**Men**							
1	774 (100)	1.00	1.00	1.00	4723.9	4747.2	1.00
2	412 (53.2)	0.45	0.86	0.78	4650.5	4701.7	0.88
	362 (46.8)	0.55		0.94			
3	445 (57.5)	0.58	0.96	0.99	4643.0	4722.1	0.98
	39 (5.0)	0.05		0.66			
	290 (37.5)	0.37		0.96			
4	91 (11.8)	0.22	0.76	0.84	4648.8	4751.1	0.83
	421 (54.4)	0.38		0.69			
	159 (20.5)	0.30		0.94			
	103 (13.3)	0.10		0.71			
5	20 (2.6)	0.13	0.71	0.73	4644.5	4746.8	0.90
	171 (22.1)	1.18		0.74			
	325 (42.0)	0.31		0.68			

For peer review only

	167 (21.6)	0.16	0.67
	91 (11.8)	0.22	0.85

AIC = Akaike Information Criterion, BIC = Bayesian Information Criterion

Table S2. Latent class marginal means with 95% confidence intervals (CI) for unhealthy behaviours by latent classes among women and men.

Unhealthy behaviours within latent classes	Women	Men
	Marginal mean (95% CI)	Marginal mean (95% CI)
**Class 1**		
Low LTPA	0.13 (0.11-0.15)	0.10 (0.06-0.16)
Non-daily F&V consumption	0.13 (0.11-0.16)	0.33 (0.26-0.40)
Insufficient sleep	0.31 (0.28-0.33)	0.32 (0.26-0.38)
Excessive alcohol use	0.08 (0.03-0.16)	0.21 (0.10-0.39)
Tobacco use	0.12 (0.06-0.22)	0.00 (0.00-1.00)
**Class 2**		
Low LTPA	0.11 (0.07-0.18)	0.02 (0.00-0.92)
Non-daily F&V consumption	0.26 (0.19-0.34)	0.12 (0.01-0.70)
Insufficient sleep	0.31 (0.24-0.38)	0.29 (0.21-0.38)
Excessive alcohol use	0.77 (0.17-0.98)	0.99 (0.00-1.00)
Tobacco use	0.69 (0.42-0.87)	0.51 (0.40-0.61)
**Class 3**		
Low physical activity	0.51 (0.27-0.74)	
Non-daily F&V consumption	0.63 (0.38-0.83)	
Insufficient sleep	0.61 (0.44-0.76)	
Excessive alcohol use	0.18 (0.07-0.41)	
Tobacco use	0.45 (0.28-0.63)	

F&V = fruit and vegetable, LTPA = leisure-time physical activity

For peer review only

Table S3. Sociodemographic characteristics (n, %) of latent classes of unhealthy behaviours among women and men.

Sociodemographic factors	Women			Men	
	Class 1 (n=2701)	Class 2 (n=380)	Class 3 (n=147)	Class 1 (n=412)	Class 2 (n=362)
**Age**					
19–29 years	827 (30.6)	176 (46.3)	46 (31.3)	93 (22.6)	104 (28.7)
30–34 years	943 (34.9)	119 (31.3)	46 (31.3)	134 (32.5)	118 (32.6)
35–39 years	931 (34.5)	85 (22.4)	55 (37.4)	185 (44.9)	140 (38.7)
**Marital status**					
Married or cohabiting	1857 (68.8)	174 (45.8)	91 (61.9)	328 (79.6)	242 (66.9)
Other	844 (31.3)	206 (54.2)	56 (38.1)	84 (20.4)	120 (33.2)
**Children living in the household**					
No	1471 (54.5)	305 (80.3)	75 (51.0)	223 (54.1)	244 (67.4)
Yes	1230 (45.5)	75 (19.7)	72 (49.0)	189 (45.9)	118 (32.6)
**Occupational class**					
Managers and professionals	802 (29.7)	71 (18.7)	22 (15.0)	154 (37.4)	87 (24.0)
Semi-professionals	1167 (43.2)	173 (45.5)	62 (42.2)	129 (31.3)	113 (31.2)
Routine non-manual workers	665 (24.6)	123 (32.4)	55 (37.4)	88 (21.4)	103 (28.5)
Manual workers	67 (2.5)	13 (3.4)	8 (5.4)	41 (10.0)	59 (16.3)

Table S4. Characteristics of all participants who responded to the Helsinki Health Study survey (2017), of those who gave their written consent to register linkages, of those who were excluded from this study [a], and of those who were finally included in this study.

	All participants who responded to the survey (n=5898)	Participants who gave their consent to register linkages (n=4864)	Excluded telephone interviewees [a] (n=651)	Other excluded participants [a] (n=211)	Participants in this study (n=4002)
**Total number of sickness absence days during the follow-up [b], median (interquartile range)**	Not available.	11 (25)	8 (24)	9 (29)	12 (26)
Health behaviours					

For peer review only

**Fresh vegetable consumption**					
Daily	4119 (70.1)	3443 (71.1)	493 (75.9)	141 (67.1)	2809 (70.5)
Non-daily	1755 (29.9)	1401 (28.9)	157 (24.2)	69 (32.9)	1175 (29.5)
**Sleep (hours)**					
7–9 hours	4573 (78.0)	3799 (78.6)	493 (75.7)	159 (76.4)	3147 (79.2)
<7 or >9 hours	1287 (22.0)	1035 (21.4)	158 (24.3)	49 (23.6)	828 (20.8)
**Alcohol use** [c]					
Once a week or less	5058 (88.9)	4153 (88.4)	610 (93.7)	179 (88.2)	3364 (87.5)
More than once a week	630 (11.1)	545 (11.6)	41 (6.3)	24 (11.8)	480 (12.5)
**Smoking** [d]					
No	4480 (76.5)	3707 (76.7)	518 (79.7)	153 (73.6)	3036 (76.4)
Currently or occasionally	1378 (23.5)	1126 (23.3)	132 (20.3)	55 (26.4)	939 (23.6)
Sociodemographic factors					
**Gender**					
Woman	4630 (78.5)	3848 (79.1)	461 (70.8)	159 (75.4)	3228 (80.7)
Man	1267 (21.5)	1016 (20.9)	190 (29.2)	52 (24.6)	774 (19.3)
**Age**					
19–29 years	1864 (31.7)	1532 (31.5)	204 (31.3)	82 (38.9)	1246 (31.1)
30–34 years	2000 (34.0)	1658 (34.1)	225 (34.6)	73 (34.6)	1360 (34.0)
35–39 years	2023 (34.4)	1674 (34.4)	222 (34.1)	56 (26.5)	1396 (34.9)
**Marital status**					
Married or cohabiting	3910 (66.3)	3248 (66.8)	427 (65.6)	129 (61.1)	2692 (67.3)
Other	1988 (33.7)	1616 (33.2)	224 (34.4)	82 (38.9)	1310 (32.7)
**Occupational class**					
Managers and professionals	1552 (27.1)	1346 (27.7)	165 (25.3)	45 (21.4)	1136 (28.4)
Semi-professionals	2233 (38.9)	1937 (39.8)	202 (31.0)	91 (43.1)	1644 (41.1)
Routine non-manual workers	1612 (28.1)	1309 (26.9)	219 (33.6)	56 (26.5)	1034 (25.8)
Manual workers	338 (5.9)	272 (5.6)	65 (10.0)	19 (9.0)	188 (4.7)

[a] Of all participants who gave their written consent to register linkages, this study excluded 1) telephone interviewees (n=651), 2) participants who had missing data on working time (n=33) after the data collection or on all health behaviours of interest (n=1), or 3) who had extreme values in health behaviours—that is, spending >24 hours together for leisure-time physical activity, sitting, and sleeping (n=166), or reporting >300 metabolic equivalent task -hours for leisure-time physical activity (n=11).

[b] The follow-up began from one day after receiving the completed survey questionnaire from a participant and continued until 31st March 2020 or until the end of one's employment contract, whichever came first.

For peer review only

[c] Participants were asked to estimate the frequency they currently use beer, wine, and spirits. The question included 10 alternatives from “never” to “daily or almost daily”.

[d] Smoking included only cigarettes (not e-cigarettes or snus).

For peer review only

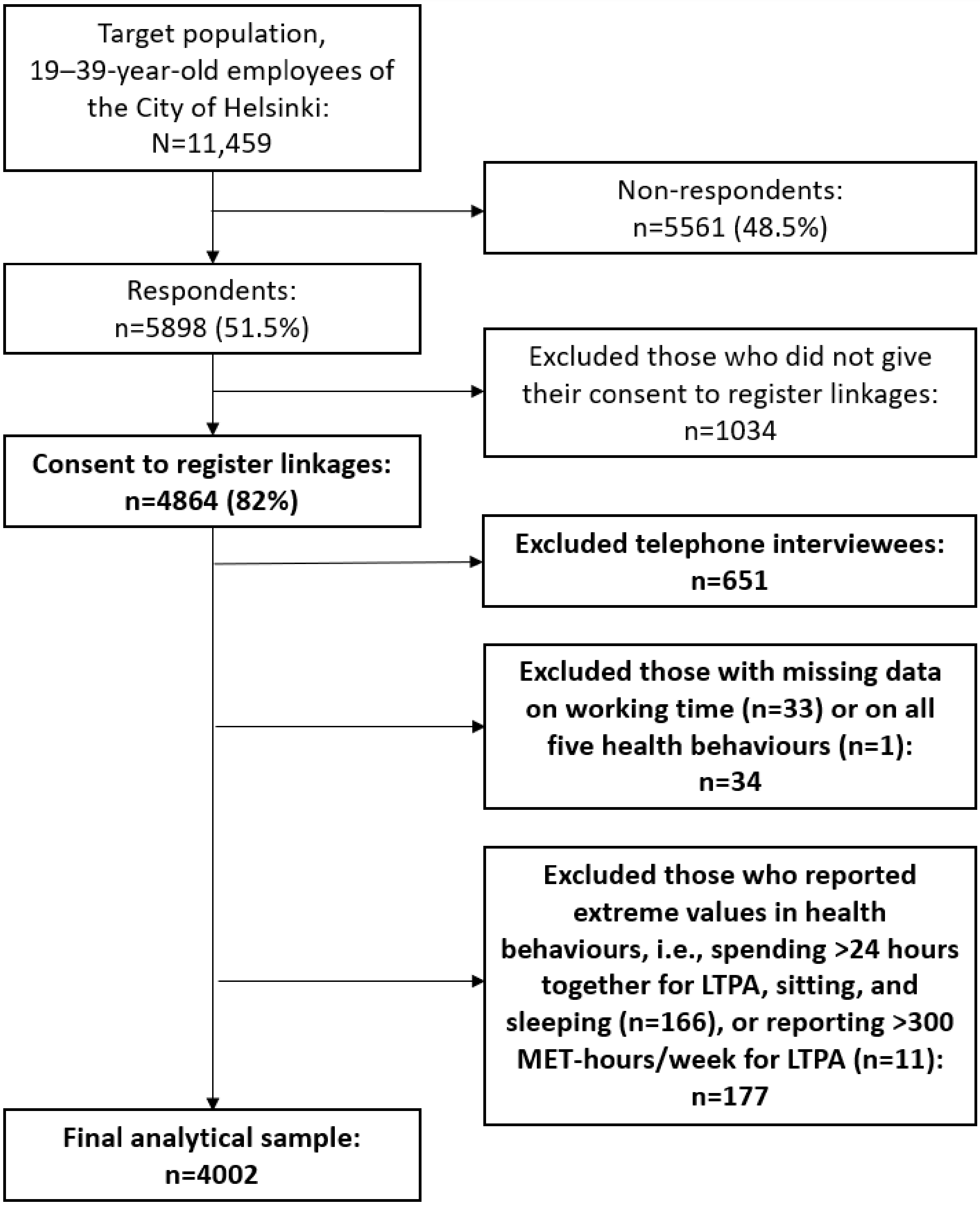

Figure S1. Flow chart: selection of the final analytical study sample.

LTPA = leisure-time physical activity, MET = metabolic equivalent task

STROBE Statement—checklist of items that should be included in reports of observational studies

	Item No	Recommendation	Page No
**Title and abstract**	1	(*a*) Indicate the study's design with a commonly used term in the title or the abstract	1
		(*b*) Provide in the abstract an informative and balanced summary of what was done and what was found	2
**Introduction**			
Background/rationale	2	Explain the scientific background and rationale for the investigation being reported	3
Objectives	3	State specific objectives, including any prespecified hypotheses	3
**Methods**			
Study design	4	Present key elements of study design early in the paper	3, 4
Setting	5	Describe the setting, locations, and relevant dates, including periods of recruitment, exposure, follow-up, and data collection	3-5
Participants	6	(*a*) *Cohort study*—Give the eligibility criteria, and the sources and methods of selection of participants. Describe methods of follow-up<br>*Case-control study*—Give the eligibility criteria, and the sources and methods of case ascertainment and control selection. Give the rationale for the choice of cases and controls<br>*Cross-sectional study*—Give the eligibility criteria, and the sources and methods of selection of participants	3-5
		(*b*) *Cohort study*—For matched studies, give matching criteria and number of exposed and unexposed<br>*Case-control study*—For matched studies, give matching criteria and the number of controls per case	-
Variables	7	Clearly define all outcomes, exposures, predictors, potential confounders, and effect modifiers. Give diagnostic criteria, if applicable	4, 5
Data sources/ measurement	8*	For each variable of interest, give sources of data and details of methods of assessment (measurement). Describe comparability of assessment methods if there is more than one group	4, 5
Bias	9	Describe any efforts to address potential sources of bias	12, 13
Study size	10	Explain how the study size was arrived at	3, 4, Figure S1
Quantitative variables	11	Explain how quantitative variables were handled in the analyses. If applicable, describe which groupings were chosen and why	4, 5
Statistical methods	12	(*a*) Describe all statistical methods, including those used to control for confounding	5, 6
		(*b*) Describe any methods used to examine subgroups and interactions	-
		(*c*) Explain how missing data were addressed	4, 13
		(*d*) *Cohort study*—If applicable, explain how loss to follow-up was addressed<br>*Case-control study*—If applicable, explain how matching of cases and controls was addressed	13

For peer review only - http://bmjopen.bmj.com/site/about/guidelines.xhtml

Cross-sectional study—If applicable, describe analytical methods taking account of sampling strategy	
(*e*) Describe any sensitivity analyses	13

Continued on next page

For peer review only

For peer review only - http://bmjopen.bmj.com/site/about/guidelines.xhtml

**Results**			
Participants	13*	(a) Report numbers of individuals at each stage of study—eg numbers potentially eligible, examined for eligibility, confirmed eligible, included in the study, completing follow-up, and analysed	4, Figure S1
		(b) Give reasons for non-participation at each stage	3, 4, Figure S1
		(c) Consider use of a flow diagram	Figure S1
Descriptive data	14*	(a) Give characteristics of study participants (eg demographic, clinical, social) and information on exposures and potential confounders	6, 7
		(b) Indicate number of participants with missing data for each variable of interest	4, Figure S1
		(c) *Cohort study*—Summarise follow-up time (eg, average and total amount)	4, 5
Outcome data	15*	*Cohort study*—Report numbers of outcome events or summary measures over time<br>*Case-control study*—Report numbers in each exposure category, or summary measures of exposure<br>*Cross-sectional study*—Report numbers of outcome events or summary measures	7, 8
Main results	16	(*a*) Give unadjusted estimates and, if applicable, confounder-adjusted estimates and their precision (eg, 95% confidence interval). Make clear which confounders were adjusted for and why they were included	8-10
		(*b*) Report category boundaries when continuous variables were categorized	4, 5, Tables 1 and 2
		(*c*) If relevant, consider translating estimates of relative risk into absolute risk for a meaningful time period	Tables 3 and 4
Other analyses	17	Report other analyses done—eg analyses of subgroups and interactions, and sensitivity analyses	9, 12, 13 Tables S2-4
**Discussion**			
Key results	18	Summarise key results with reference to study objectives	10
Limitations	19	Discuss limitations of the study, taking into account sources of potential bias or imprecision. Discuss both direction and magnitude of any potential bias	12, 13
Interpretation	20	Give a cautious overall interpretation of results considering objectives, limitations, multiplicity of analyses, results from similar studies, and other relevant evidence	11-13
Generalisability	21	Discuss the generalisability (external validity) of the study results	13
**Other information**			
Funding	22	Give the source of funding and the role of the funders for the present study and, if applicable, for the original study on which the present article is based	17

*Give information separately for cases and controls in case-control studies and, if applicable, for exposed and unexposed groups in cohort and cross-sectional studies.

For peer review only - http://bmjopen.bmj.com/site/about/guidelines.xhtml

For peer review only

Note: An Explanation and Elaboration article discusses each checklist item and gives methodological background and published examples of transparent reporting. The STROBE checklist is best used in conjunction with this article (freely available on the Web sites of PLoS Medicine at http://www.plosmedicine.org/, Annals of Internal Medicine at http://www.annals.org/, and Epidemiology at http://www.epidem.com/). Information on the STROBE Initiative is available at www.strobe-statement.org.

For peer review only

For peer review only - http://bmjopen.bmj.com/site/about/guidelines.xhtml

BMJ Open

Latent classes of unhealthy behaviours and their associations with subsequent sickness absence: a prospective register-linkage study among Finnish young and early midlife employees

Journal:	*BMJ Open*
Manuscript ID	bmjopen-2022-070883.R1
Article Type:	Original research
Date Submitted by the Author:	21-Mar-2023
Complete List of Authors:	Salmela, Jatta; University of Helsinki Faculty of Medicine, Department of Public Health<br>Lahti, Jouni; University of Helsinki Faculty of Medicine, Department of Public Health<br>Kanerva, Noora; University of Helsinki Faculty of Agriculture and Forestry, Department of Food and Nutrition<br>Rahkonen, Ossi; University of Helsinki Faculty of Medicine, Department of Public Health<br>Kouvonen, Anne; Helsingin Yliopisto, Faculty of Social Sciences; Queen's University Belfast,<br>Lallukka, Tea; University of Helsinki Faculty of Medicine, Department of Public Health
**Primary Subject Heading**:	Occupational and environmental medicine
Secondary Subject Heading:	Public health, Epidemiology
Keywords:	PREVENTIVE MEDICINE, EPIDEMIOLOGIC STUDIES, OCCUPATIONAL & INDUSTRIAL MEDICINE, PUBLIC HEALTH

SCHOLARONE™
Manuscripts

I, the Submitting Author has the right to grant and does grant on behalf of all authors of the Work (as defined in the below author licence), an exclusive licence and/or a non-exclusive licence for contributions from authors who are: i) UK Crown employees; ii) where BMJ has agreed a CC-BY licence shall apply, and/or iii) in accordance with the terms applicable for US Federal Government officers or employees acting as part of their official duties; on a worldwide, perpetual, irrevocable, royalty-free basis to BMJ Publishing Group Ltd ("BMJ") its licensees and where the relevant Journal is co-owned by BMJ to the co-owners of the Journal, to publish the Work in this journal and any other BMJ products and to exploit all rights, as set out in our licence.

The Submitting Author accepts and understands that any supply made under these terms is made by BMJ to the Submitting Author unless you are acting as an employee on behalf of your employer or a postgraduate student of an affiliated institution which is paying any applicable article publishing charge ("APC") for Open Access articles. Where the Submitting Author wishes to make the Work available on an Open Access basis (and intends to pay the relevant APC), the terms of reuse of such Open Access shall be governed by a Creative Commons licence – details of these licences and which Creative Commons licence will apply to this Work are set out in our licence referred to above.

Other than as permitted in any relevant BMJ Author's Self Archiving Policies, I confirm this Work has not been accepted for publication elsewhere, is not being considered for publication elsewhere and does not duplicate material already published. I confirm all authors consent to publication of this Work and authorise the granting of this licence.

For peer review only

TITLE PAGE

Title of the article:

Latent classes of unhealthy behaviours and their associations with subsequent sickness absence: a prospective register-linkage study among Finnish young and early midlife employees

Authors:

Jatta Salmela [1]*, Jouni Lahti [1], Noora Kanerva [2], Ossi Rahkonen [1], Anne Kouvonen [3,4], Tea Lallukka [1]

Affiliations:

[1] Department of Public Health, University of Helsinki, Helsinki, Finland

[2] Department of Food and Nutrition, University of Helsinki, Helsinki, Finland

[3] Faculty of Social Sciences, University of Helsinki, Helsinki, Finland

[4] Centre for Public Health, Queen's University Belfast, Belfast, Northern Ireland

***Corresponding author:**

Jatta Salmela, PhD

Postal address:

Department of Public Health

PO BOX 20 (Tukholmankatu 8 B)

00014 University of Helsinki

Finland

email: jatta.salmela@helsinki.fi

Word count, excluding title page, abstract, references, figures, and tables:

3719

Keywords:

Employment, Health Behavior, Latent Class Analysis, Sick Leave

For peer review only

ABSTRACT

Objectives: Unhealthy behaviours are associated with increased sickness absence (SA), but few studies have considered person-oriented approach in these associations. Using latent class analysis, we examined clustering of unhealthy behaviours among Finnish municipal employees and their associations with subsequent SA.

Design: A prospective register-linkage study.

Setting: Unhealthy behaviours (low leisure-time physical activity, non-daily fruit and vegetable consumption, insufficient sleep, excessive alcohol use, and tobacco use) were derived from the Helsinki Health Study questionnaire survey, collected in 2017 among 19–39-year-old employees of the City of Helsinki, Finland.

Participants: A total of 4002 employees (81% women) of the City of Helsinki, Finland.

Primary outcome measures: The questionnaire data were prospectively linked to employer's SA register through March 2020. Associations between latent classes of unhealthy behaviours and subsequent SA (1–7 days / 8+ days / all lengths) were examined using negative binomial regression.

Results: Among women, a 3-class latent class model was selected: 1) few unhealthy behaviours (84%), 2) excessive alcohol and tobacco use (12%), and 3) several unhealthy behaviours (5%). Women belonging to Classes 2 and 3 had increased SA rates compared to those in Class 1, regardless of the length of SA spells. Among men, a 2-latent class model was selected: 1) few unhealthy behaviours (53%) and 2) several unhealthy behaviours (47%). Men belonging to Class 2 had increased rates of 1–7 days' SA compared to men in Class 1.

Conclusions: This study suggests that preventive actions aiming to reduce employees' SA should consider simultaneously several unhealthy behaviours. Targeted interventions may benefit of identifying the clustering of these behaviours among occupational groups.

STRENGTHS AND LIMITATIONS OF THIS STUDY

- Applying person-oriented approach enabled us to identify unobserved population groups that share similar patterns of unhealthy behaviours.
- We could link questionnaire data on employees' health behaviours to employers' register data on different lengths of sickness absence (1–7 days, 8+ days, and all lengths) with a mean follow-up time of 2.13 years.
- Self-reported measures of health behaviours may biased, which may influence the identified latent classes of unhealthy behaviours.
- Although the large proportion of women well represents the gender distribution in the target population and in the municipal sector in Finland in general, the small number of men limits the interpretation of the findings among men and the gender comparisons.

For peer review only

INTRODUCTION

Health behaviours have a major contribution to employees' sickness absence (SA). It is estimated that 15–31% of SA could be attributed to unhealthy behaviours(1,2). In addition to their independent contributions, health behaviours can mediate some of the effects of working conditions and socioeconomic circumstances on SA: for instance, unhealthy behaviours (e.g., smoking and binge drinking) may be used to cope with stressful working conditions(3,4). In Finland, as in most high-income countries, the leading causes for medically certified SA are mental and musculoskeletal disorders(5,6). Unhealthy behaviours, such as low physical activity, poor sleep, binge drinking, and smoking, have been associated with both medically certified SA and self-certified SA(1,7–9). However, the results are not fully consistent(2,10). Diet appears to have a minor contribution to SA(2,3,7,11), but since obesity is consistently associated with SA(12), dietary aspects—which play a key role in weight management—should not be neglected. The possible mechanisms and pathways between unhealthy behaviours and SA have been suggested to proceed, for instance, through increased risk for chronic diseases, risk-taking lifestyle, and decreased immune system (leading to, e.g., common cold)(1–3). Additionally, working conditions and socioeconomic circumstances may explain some of the associations(3).

Accumulation of several unhealthy behaviours have been shown to increase SA more than individual unhealthy behaviours(2,7,13). Our previous study on midlife and ageing Finnish employees found that the joint contribution of physical inactivity and smoking was especially detrimental for employer's cost of 1–14 days' SA(13). Health behaviours tend to be clustered within population groups(14,15), and these clusters may have synergistic effects on health(14). Considering clustering of unhealthy behaviours can help policymakers and researchers to design targeted interventions to improve employees' health behaviours and reduce SA. However, to best of our knowledge, no studies have examined how clustering of unhealthy behaviours is associated with SA. Clustering techniques, such as latent class analyses, can provide more holistic approach on how health behaviours contribute to SA compared to summary indices(2,7) that consider each risk factor equally and disregard their interconnections(16).

This study aimed to identify latent classes of five unhealthy behaviours among 19–39-year-old employees of the City of Helsinki, Finland. Furthermore, using linkage to employer's SA register, we aimed to examine the associations between the latent classes with subsequent SA.

METHODS

Data and study population

This study is a part of the Helsinki Health Study of young and early midlife employees of the City of Helsinki(17). The City of Helsinki is the largest employer in Finland with around 38,000 employees and hundreds of occupational titles. The target population included 11,459 employees who were born in 1978 or later, who had a job contract of at least 50% of regular work hours per week, and whose employment contract had lasted at least 4 months before the data collection began in autumn 2017. Data were collected via online

For peer review only

and mailed questionnaires, which included a large variety of questions related to participants' social and economic characteristics and health behaviours. Additionally, shorter telephone interviews were conducted to target those who did not respond online or via mail. The overall response rate was 51.5% (n=5898)(17). The survey data were linked to employer's personnel register data for those who gave their written informed consent (82% of respondents, n=4864). We excluded telephone interviewees (n=651) since the interviews did not include all the variables of interest in this study, as well as participants who had missing data on working time or on all health behaviours of interest (n=34), or who had extreme values in health behaviours (n=177) (online supplemental file 1, figure S1). The final analytical sample included 4002 participants (81% women).

Health behaviour measures

We included five unhealthy behaviours from the survey: 1) low leisure-time physical activity (LTPA), 2) non-daily fruit and vegetable (F&V) consumption, 3) insufficient sleep, 4) excessive alcohol use, and 5) tobacco use (see online supplemental file 2). Since it is not computationally possible to include too many multi-categorical variables or variables with very small group sizes in the LCA models, we dichotomised all health behaviour measures taking into consideration current guidelines and group sizes in the variables. Participants were inquired about their weekly volume and intensity of exercise in their leisure time or while commuting during the past 12 months. Four levels of intensity were provided, and they were multiplied by the time used per week in LTPA, yielding weekly metabolic equivalent task (MET) -hours(7). Then, we dichotomised participants to those with high/moderate LTPA and those with low LTPA by using a cut-point of 20 MET-hours. Twenty MET-hours equals, for instance, 2.5 hours brisk walking and 1.5 hours walking, which was considered closely to correspond current guidelines(18,19).

F&V consumption during the past 4 weeks was inquired using a 14-item food frequency questionnaire. We dichotomised participants into daily (once a day or more F or V) and non-daily F&V consumers. Subjective experience of sleep was used as a sleep measure. We dichotomised participants into those who estimated that they sleep always/often sufficiently and those who estimated that they sleep seldom/never sufficiently. Alcohol use combined the measures of total weekly alcohol use and binge drinking behaviour. Weekly alcohol use was calculated based on participants' estimation on how often they consume different alcohol types (beer/cider, wine, and spirits). Seven frequency alternatives were provided for each question, with one unit of alcohol equalling 12g ethanol. Based on the Finnish Current Care Guidelines on alcohol consumption(20), 7 weekly units for women and 14 weekly units for men (i.e., moderate risk levels) were considered as cut-points. Additionally, participants were asked how often they drink six units or more at once (six response alternatives). We dichotomised those drinking less than 7/14 (women/men) units per week and binge drinking less than once a month into moderate alcohol users, and others to excessive alcohol users. Participants were provided four alternatives to estimate their use of tobacco products (cigarettes, e-cigarettes, and snus): 'yes, daily', 'sporadically', 'not nowadays', and 'never'. We dichotomised participants into never-/ex-users, and those using daily/occasionally tobacco products.

For peer review only

Sickness absence measures

The data on SA were derived from the personnel register of the City of Helsinki. The follow-up of SA began from one day after receiving the completed survey questionnaire and continued until 31st March 2020 or until the end of one's employment contract, whichever came first. The time limit was selected so that we could exclude the potential influence of the COVID-19 pandemic to the results. The mean follow-up time was 2.13 years. We combined overlapping and consecutive SA spells and divided them into SA spells of 1–7 days and 8+ days. During the follow-up, the City of Helsinki had a policy that 1–7 days' SA could be given to an employee by their supervisor, nurse, occupational physiotherapist, or physician, whereas 8+ days' SA required a medical certification approved by a physician. The policy was the same for all employees. Additionally, we analysed all lengths' SA.

Covariates

We stratified all analyses by gender (woman/man), given that notable gender differences have been observed in SA and health behaviours(21,22), and clustering of health behaviours may vary by gender(15). Age included categories of 19–29, 30–34, and 35–39 years. Marital status was derived from the questionnaire and was dichotomised into married/cohabiting and other. In the questionnaire, participants were inquired whether they had any 0–18-year-old children living in their household ('yes/no'). Occupational class was derived from the employer's personnel register for those who gave their informed consent for register linkage (82%), and for others, the information was derived from the questionnaire. Occupational class included four groups: managers and professionals (e.g., teachers and physicians), semi-professionals (e.g., nurses and foremen), routine non-manual workers (e.g., childcare and elderly care workers), and manual workers (e.g., care assistants). It is noteworthy that in recent years the City of Helsinki has outsourced most of their manual work (e.g., cleaning and transport work), and therefore the proportion of manual workers employed by the city is now very low. Prior SA, especially past year's SA, is known to predict future SA(10,23). Thus, we included prior SA of any length during one year before participant's response to the questionnaire.

Statistical methods

We first tabulated descriptive statistics by key exposure variables. Then, incidence of SA days per 10 person-years were calculated by individual health behaviours using negative binomial regression. We identified latent classes of unhealthy behaviours using latent class analysis (LCA). LCA is a person-oriented statistical procedure to detect latent (unobserved) subgroups, which share certain outward characteristics, within a heterogeneous population(24,25). This subtype of structural equation modelling uses categorical indicator variables to form latent classes based on the indicator variables. Participants are assigned to the latent classes based on their probability of class membership. We used the following statistical criteria for selecting the most optimal number of latent classes: Bayesian information criterion (BIC), Akaike information criterion (AIC), average posterior probabilities of class membership (>0.8), class sizes (>50 cases or >5% of the sample), and

For peer review only

entropy (>0.8)(25). One- to five-class models were run, and the model fit evaluation process is shown in online supplemental file 1, table S1. Additionally, we considered the interpretability of the models to select the final models(25).

We used negative binomial regression to examine associations between latent classes of unhealthy behaviours and subsequent SA due to overdispersion in the data. Rate ratios (RRs) and predictive margins with 95% confidence intervals (CIs) were calculated. Model 1 was adjusted for age, and model 2 further for marital status, children living in the household, occupational class, and prior SA. Natural logarithm of the follow-up time was included as an offset variable in all models to consider differences in the follow-up times between participants. All analyses were performed using STATA version 17.0 (StataCorp LLC, College Station, TX, USA).

Patient and public involvement

Patients or the public were not involved in this study.

RESULTS

Characteristics of study population

Most participants had at least one unhealthy behaviour (67% of women and 83% of men), whereas under 1% of women and men had all five unhealthy behaviours. Low LTPA and insufficient sleep were equally common among women and men (table 1). However, non-daily F&V consumption, excessive alcohol use, and tobacco use were more common among men than among women. Most women and men were married/cohabiting and around 40% had children living in their household. Only 3% of women were manual workers while the corresponding proportion for men was 13%.

Table 1. Characteristics of the participants by sociodemographic factors and health behaviours among women and men.

**Total**	**Women (n, %)**	**Men (n, %)**
	3228 (80.7)	774 (19.3)
Health behaviours		
**Leisure-time physical activity [a]**		
High or moderate activity	2689 (84.4)	651 (85.3)
Low activity	499 (15.7)	112 (14.7)
**Fruit and vegetable consumption**		
Daily	2595 (80.5)	463 (60.0)
Non-daily	629 (19.5)	309 (40.0)
**Sleep sufficiency**		
Mostly sufficient sleep	2146 (66.9)	521 (67.8)
Insufficient sleep	1064 (33.2)	248 (32.3)
**Alcohol use [b]**		
Moderate	2492 (79.9)	423 (55.8)
Excessive	626 (20.1)	335 (44.2)
**Tobacco use [c]**		

For peer review only

No	2430 (75.8)	471 (61.1)
Currently or occasionally	777 (24.2)	300 (38.9)
Sociodemographic factors		
**Age**		
19–29 years	1049 (32.5)	197 (25.5)
30–34 years	1108 (34.3)	252 (32.6)
35–39 years	1071 (33.2)	325 (42.0)
**Marital status**		
Married or cohabiting	2122 (65.7)	570 (73.6)
Other	1106 (34.3)	204 (26.4)
**Children living in the household**		
No	1851 (57.3)	467 (60.3)
Yes	1377 (42.7)	307 (39.7)
**Occupational class**		
Managers and professionals	895 (27.7)	241 (31.1)
Semi-professionals	1402 (43.4)	242 (31.3)
Routine non-manual workers	843 (26.1)	191 (24.7)
Manual workers	88 (2.7)	100 (12.9)

[a] Leisure-time physical activity (LTPA) included physical activity during leisure time and active commuting. High or moderate LTPA was considered as ≥20 metabolic equivalent task (MET) -hours per week and low LTPA as <20 MET-hours per week.

[b] Moderate alcohol use: ≤7 units of alcohol per month and binge drinking less than once a month among women, and ≤14 units of alcohol per month and binge drinking less than once a month among men. Excessive alcohol use: >7 units of alcohol per month and binge drinking less than once a month among women, and >14 units of alcohol per month and binge drinking less than once a month among men.

[c] Tobacco use included use of cigarettes, e-cigarettes, and snus.

During the follow-up, we recorded altogether 117 SA days/10 person-years for women and 93 SA days/10 person-years for men. Of women, 15% had no 1–7 days' SA, 69% had no 8+ days' SA, and 18% had no SA of any length during the follow-up. For men, the corresponding figures were 18%, 75% and 17%. Participants with healthier behaviours had less SA than those with unhealthier behaviours in general (table 2). However, F&V consumption and alcohol use were exceptions among men in terms of 8+ days' SA: those with healthier behaviour had more or equally 8+ days' SA compared to those with unhealthier behaviour. When scrutinising all lengths' SA, the greatest differences between healthy and unhealthy behaviour groups were seen in tobacco use among women and in sleep among men.

For peer review only

For peer review only - http://bmjopen.bmj.com/site/about/guidelines.xhtml

Table 2. Incidence of sickness absence days per 10 person-years with 95% confidence intervals (in parenthesis), by health behaviours among women and men.

Health behaviours	Women (n=3228)			Men (n=774)		
	1–7 days' SA	8+ days' SA	All lengths' SA	1–7 days' SA	8+ days' SA	All lengths' SA
**Leisure-time physical activity** [a]						
High or moderate activity	62 (59-64)	67 (58-77)	128 (122-135)	50 (46-55)	40 (30-55)	90 (81-101)
Low activity	72 (66-80)	74 (55-99)	146 (130-163)	64 (51-81)	52 (26-104)	116 (89-152)
**Fruit and vegetable consumption**						
Daily	61 (58-63)	67 (58-78)	127 (121-134)	47 (42-52)	45 (31-65)	92 (81-105)
Non-daily	74 (68-81)	74 (57-96)	148 (133-163)	59 (52-68)	36 (24-56)	96 (83-112)
**Sleep sufficiency**						
Mostly sufficient sleep	61 (58-64)	59 (51-70)	120 (113-127)	46 (41-51)	32 (22-46)	77 (69-88)
Insufficient sleep	68 (63-73)	88 (72-108)	155 (143-169)	64 (56-74)	62 (40-95)	127 (108-149)
**Alcohol use** [b]						
Moderate	60 (58-63)	66 (57-76)	125 (119-132)	49 (44-55)	42 (29-61)	91 (80-104)
Excessive	75 (69-82)	81 (62-105)	156 (140-172)	56 (50-64)	42 (27-66)	99 (85-115)
**Tobacco use** [c]						
No	58 (56-61)	62 (53-72)	119 (113-126)	48 (43-53)	37 (26-53)	84 (74-96)
Currently or occasionally	79 (74-85)	91 (72-114)	169 (155-185)	59 (52-67)	49 (32-77)	108 (93-126)

[a] Leisure-time physical activity (LTPA) included physical activity during leisure time and active commuting. High or moderate LTPA was considered as ≥20 metabolic equivalent task (MET) - hours per week and low LTPA as <20 MET-hours per week.

[b] Moderate alcohol use: ≤7 units of alcohol per month and binge drinking less than once a month among women, and ≤14 units of alcohol per month and binge drinking less than once a month among men. Excessive alcohol use: >7 units of alcohol per month and binge drinking less than once a month among women, and >14 units of alcohol per month and binge drinking less than once a month among men.

[c] Tobacco use included use of cigarettes, e-cigarettes, and snus.

For peer review only

Latent classes of unhealthy behaviours

The most optimal number of latent classes of unhealthy behaviours was three for women and two for men (figure 1 and online supplemental file 1, table S1). Although model fit statistics preferred the 2-class solution for women, 3 classes were selected as they were interpretatively reasonable and provided new information about the data. Most statistical criteria preferred the 3-class solution for men, but we selected 2 classes due to one too small class size (n=39) in the 3-class solution. Marginal means for each unhealthy behaviour within latent classes are shown in supplemental file 1, table S2.

[figure 1 here]

Of women, 84% had the highest posterior probability for belonging to Class 1, and for Classes 2 and 3, the corresponding proportions were 12% and 5% (figure 1a). Class 1 was characterised by overall low probabilities of having unhealthy behaviours. Class 2 was characterised especially by excessive alcohol use and tobacco use, whereas probabilities for other unhealthy behaviours were somewhat low. In Class 3, there were increased probabilities for all other unhealthy behaviours except excessive alcohol use. Of men, 53% had the highest posterior probability for belonging to Class 1, and 47% to Class 2 (figure 1b). Class 1 was characterised by somewhat low probabilities of having any unhealthy behaviours. The probabilities of having unhealthy behaviours were overall increased in Class 2, and it was especially characterised by low LTPA, non-daily F&V consumption, and excessive alcohol use.

Associations between latent classes of unhealthy behaviours and sickness absence

Women belonging to Classes 2 and 3 had increased SA rates compared to Class 1 (table 3). However, the associations with 8+ days' SA were not statistically significant. Women belonging to Classes 2 and 3 had increased rates of 1–7 days' SA even after adjustment for age, marital status, children living in the household, occupational class, and prior SA (table 3, M2). Men belonging to Class 2 had increased SA rates compared to Class 1 (table 4). However, statistically significant association was found only for 1–7 days' SA in the age-adjusted model (table 4, M1). This association attenuated after further adjustments (table 4, M2), especially after adjustment for occupational class.

Table 3. Associations between latent classes of unhealthy behaviours and sickness absence (SA) among women. Rate ratios (RR) and predictive margins with 95% confidence intervals (CI) from negative binomial regression models [a] are shown.

	1–7 days' SA		8+ days' SA		All lengths' SA	
**Latent class**	**M1: Adjusted for age**	**M2: M1 + marital status, children living in the household, occupational class, prior SA [b]**	**M1: Adjusted for age**	**M2: M1 + marital status, children living in the household, occupational class, prior SA [b]**	**M1: Adjusted for age**	**M2: M1 + marital status, children living in the household, occupational class, prior SA [b]**

For peer review only

	**RR (95 % CI)**					
**Class 1**	ref.	ref.	ref.	ref.	ref.	ref.
**Class 2**	1.39 (1.24-1.57)	1.21 (1.08-1.36)	1.37 (0.92-2.02)	1.35 (0.92-1.97)	1.39 (1.20-1.61)	1.29 (1.13-1.48)
**Class 3**	1.37 (1.14-1.64)	1.19 (1.00-1.42)	1.31 (0.72-2.38)	1.22 (0.68-2.18)	1.34 (1.07-1.67)	1.18 (0.96-1.46)
	**Predictive margins (95 % CI)**					
**Class 1**	12.6 (12.1-13.2)	12.2 (11.7-12.7)	13.7 (11.8-15.6)	10.8 (9.3-12.2)	26.2 (24.8-27.5)	23.0 (21.9-24.1)
**Class 2**	17.6 (15.6-19.6)	14.8 (13.2-16.4)	18.7 (11.8-25.5)	14.5 (9.3-19.7)	36.5 (31.5-41.5)	29.7 (25.9-33.5)
**Class 3**	17.3 (14.2-20.3)	14.5 (12.1-17.0)	17.9 (7.4-28.3)	13.1 (5.7-20.5)	35.0 (27.4-42.6)	27.2 (21.7-32.7)

[a] Natural logarithm of the follow-up time is included in the models as an offset variable.

[b] Prior sickness absence of all lengths one year before the follow-up, divided by the working time in years during the one year's period.

Table 4. Associations between latent classes of unhealthy behaviours and sickness absence (SA) among men. Rate ratios (RR) and predictive margins with 95% confidence intervals (CI) from negative binomial regression models [a] are shown.

	**1–7 SA days**		**8+ SA days**		**SA days of all lengths**	
**Latent class**	**M1: Adjusted for age**	**M2: M1 + marital status, children living in the household, occupational class, prior SA [b]**	**M1: Adjusted for age**	**M2: M1 + marital status, children living in the household, occupational class, prior SA [b]**	**M1: Adjusted for age**	**M2: M1 + marital status, children living in the household, occupational class, prior SA [b]**
	**RR (95 % CI)**					
**Class 1**	ref.	ref.	ref.	ref.	ref.	ref.
**Class 2**	1.23 (1.04-1.45)	1.11 (0.95-1.31)	1.16 (0.65-2.06)	1.01 (0.58-1.77)	1.20 (0.98-1.46)	1.06 (0.88-1.28)
	**Predictive margins (95 % CI)**					
**Class 1**	10.0 (8.8-11.1)	9.5 (8.5-10.6)	8.2 (5.0-11.4)	6.3 (4.0-8.7)	18.2 (15.7-20.7)	16.2 (14.2-18.2)
**Class 2**	12.2 (10.8-13.7)	10.6 (9.4-11.8)	9.6 (5.6-13.5)	6.4 (3.8-8.9)	21.8 (18.7-25.0)	17.2 (14.9-19.5)

[a] Natural logarithm of the follow-up time is included in the models as an offset variable.

[b] Prior sickness absence of all lengths one year before the follow-up, divided by the working time in years during the one year's period.

DISCUSSION

Summary of the main findings

By using the LCA method, we selected three latent classes of unhealthy behaviours among women, characterised as follows: 1) few unhealthy behaviours, 2) excessive alcohol use and tobacco use, and 3) several unhealthy behaviours. Among men, we selected two latent classes with the following characteristics: 1) few unhealthy behaviours and 2) several unhealthy behaviours. Women in Classes 2 and 3, and men in Class 2 had increased rates of 1–7 days' SA compared to Class 1. The associations between latent classes of unhealthy behaviours and 8+ days' SA were not statistically significant either among women or men.

For peer review only

Comparisons to the previous literature

The majority of women and men were most likely to belong to Class 1, characterised by overall healthier behaviours. Similarly, a systematic review of the clustering of smoking, nutrition, alcohol, and physical activity in adults found that a majority of included studies reported a 'healthy' cluster, characterised by the absence of any unhealthy behaviours(14). This was not affected by in how health behaviours were defined or by the used clustering analysis method(14). Some more recent studies have also identified a class of overall healthier behaviours(26–28). Additionally, previous studies have found especially alcohol consumption and smoking often clustering(14,15), which we also observed in women in Class 2. However, in men, this was not observed with two latent classes. Further analyses revealed that with a 3-class solution in men, clustering of excessive alcohol use and tobacco use existed similarly as in women. Clustering of low LTPA and non-daily F&V consumption, which we observed in Class 2 among men, has been found in many of the previous studies (14,15). However, Noble's et al. (2015) systematic review did not find clustering of physical inactivity, poor diet, and excess alcohol use—the combination that we found to reflect Class 2 in men—in any of the included studies(14). Finally, clustering of several unhealthy behaviours have been observed in many previous studies(14,26), which we also could observe in Class 3 among women and Class 2 among men.

To our knowledge, no previous studies have examined associations between latent classes of unhealthy behaviours and SA, although the relationship between health behaviours and SA are broadly studied in general. Concerning single unhealthy behaviours, previous studies have associated low LTPA(1,9,29,30), poor sleep(31,32), excessive alcohol use(1,33), and smoking(1,2,29,30,32,34) with SA, while the contribution of poor diet to SA has been modest(2,3,7,11,29). Although diet has not been associated with SA as strongly as other health behaviours, we found that inadequate F&V consumption was one major characteristic of Class 3 among women and Class 2 among men—the classes that were associated with increased subsequent SA. Our previous study on midlife and older employees also showed that the joint contribution of F&V consumption and LTPA to SA might be stronger than the individual contribution of LTPA(11). However, since F&V consumption reflects only partially participants' overall diet, further studies that consider dietary patterns more comprehensively are needed.

Our previous study showed that midlife and older employees with three or more unhealthy behaviours had higher cost of 1–14 days' SA than employees without any unhealthy behaviours(7). In particular, low LTPA, poor sleep, and current smoking increased the SA cost(7). Another study by our research group found that the joint contributions of low LTPA, poor sleep, and smoking to 1–14 days' SA cost were stronger than the contributions of these health behaviours individually(13). A Norwegian study on general working population found that an exposure to multiple health-related risk factors (low physical activity, unhealthy diet, obesity, and current smoking) was associated with increased subsequent 1–14 days' and 15+ days' SA(2). Additionally, a Danish study on private sector employees found that exposure to multiple health-related risk factors (dyssomnia, overweight, unhealthy food habits, smoking, excessive alcohol use, and low physical activity)

For peer review only

were related to increased 1–14 days' SA(32). These previous findings are concordant with our finding which indicated that the latent classes of several unhealthy behaviours (Class 3 for women and Class 2 for men) were related to increased SA rates.

We found that latent classes of unhealthy behaviours were associated with 1–7 days' SA among women and men, but not statistically significantly with 8+ days' SA. In contrast, previous studies have found stronger associations for longer SA spells(2,3). There is some evidence that younger employees have more often short-term SA and older employees long-term SA(34,35), which may partly explain our findings. Another explanation is that the follow-up period of 2.13 years may not be long enough to ensure the associations with 8+ days' SA since their rate during the follow-up was relatively low.

Previous research has shown that clustering of unhealthy behaviours is strongly related to socioeconomic position(14,15,26,27,36). Similarly, we found that managers and professionals were more likely to belong to the 'healthiest' latent classes (online supplemental file 1, table S3). However, occupational class together with other sociodemographic factors explained only some of the associations between the latent classes of unhealthy behaviours and SA. Since socioeconomic differences in SA are visible already among young employees(37) and employees in the lower socioeconomic positions are more likely to have adverse working conditions (e.g., higher exposure to physical workload) that are strongly related to increased SA(29,34,38,39), these factors should not be neglected when designing targeted health behaviour interventions at workplaces. Burdorf and Robroek (2018) have suggested that preventive interventions should simultaneously consider improvements in working conditions and health behaviours, and they should be targeted to high-risk and low-educated population groups(40). These could include, for example, reducing physical and psychosocial strenuousness of work while making healthy choices more easily available, for instance, by supporting active commuting, providing exercise facilities, improving availability of staff canteens providing healthy meals, and improving accessibility to occupational health services. Identifying occupational groups among whom these conditions are insufficient and among whom unhealthy behaviours are common is crucial for employers. Additionally, given that younger age predisposes to clustering of unhealthy behaviours(online supplemental file 1, table S3,(14,26,27)—and that health behaviours may be more difficult to modify the older individuals are—preventive actions are especially needed among young employees in the lower socioeconomic positions.

Limitations and strengths

This study has a few limitations that should be considered. First, health behaviours were self-reported, thus biased estimates are possible. Second, the used cut-points in the health behaviour measures may have affected the identified latent classes. We tested various options and made the final decisions of the dichotomisations based on their consistency with the current guidelines and their proportions in the data. Third, the used cut-point in SA (1–7/8 days) complicates the comparisons to other studies since many previous studies have used cut-points of 3/4 days or 14/15 days to distinguish short-term SA from long-term SA. However, 15+ days' SA

For peer review only

were rare in this study population, and the changes made in the SA practices by the City of Helsinki during the follow-up period supported using the chosen cut-point. SA spells of 8+ days were still relatively rare in the study population, and a longer follow-up period could have strengthened the interpretation of the findings. Fourth, the small number of men limits the interpretation of the findings among men and the gender comparisons. The large proportion of women well represents, however, the gender distribution in the target population and in the municipal sector in Finland in general.

Fifth, missing data and non-participation may affect the findings. LCA uses maximum likelihood estimation and assumes missingness at random(25), thus missing data on health behaviours were allowed. However, we have carefully examined the representativeness of the data and found them to satisfactorily represent the target population (N=11,459)(17). The response rate to the survey was moderate (51.5%), and the non-respondents were somewhat more often men, manual workers, had lower income, and had more 15+ days' SA(17). Additionally, the participants included in this current study were more often of higher occupational class (online supplemental file 1, table S4); thus, our results may be slightly conservative(26,36). However, the sensitivity analyses showed that the final analytical sample (n=4002) highly resembled the full sample (n=5898) in terms of health behaviours and socioeconomic characteristics (online supplemental file 1, table S4). In addition to the use of comprehensive survey data, a further strength of this study is that we could link the questionnaire survey to employer's SA registers, which is rarely possible. Furthermore, using the person-oriented LCA method to deepen our understanding on the associations between unhealthy behaviours and SA is a novel approach in this study area.

CONCLUSIONS

This study identified three latent classes of unhealthy behaviours for women and two for men. The 'healthiest' classes among women and men showed the lowest SA rates. The associations of the latent classes of unhealthy behaviours were stronger with 1–7 days' than with 8+ days' SA. Thus, by considering the clustering of unhealthy behaviours among young and early midlife employees and intervening in them may reduce employees' short-term SA at least. Occupational class together with other sociodemographic factors explained some of the found associations, thus special focus on employees with lower occupational positions is needed.

DATA AVAILABILITY STATEMENT

Data are available upon reasonable request. The Helsinki Health Study survey data cannot be made publicly available due to strict data protection laws and regulations. The data can only be used for scientific research and to the research group's cooperation partners with a reasonable request and study plan. More information on the availability of the survey data can be inquired from the Helsinki Health Study research group (kttl-hhs@helsinki.fi). Register data cannot be shared.

For peer review only

ETHICS STATEMENTS

Patient consent for publication

Not applicable. All participants have been informed about their rights and other ethical considerations (e.g., no participant can be identified from published results, voluntary participation, possibility to withdraw from the study, and how and where the data are used) prior to their inclusion in the study.

Ethics approval

The Helsinki Health Study protocol has been approved by the ethics committees of Department of Public Health, University of Helsinki, and the health authorities of the City of Helsinki. The permission to have access to the employer's personnel register data was obtained from the City of Helsinki. Appropriate ethical aspects have been followed in all phases of the study, according to the Declaration of Helsinki.

ACKNOWLEDGEMENTS

The authors thank all participating employees and the personnel administration of the City of Helsinki.

FOOTNOTES

Twitter: @JattaSalmela, @NooraKaroliinaK, @OssiRahkonen, @AKouvonen, @TeaLallukka, @HHS_helsinkiuni

Contributors: JS was the primary author of the paper, performed the statistical analyses, and is responsible for the overall content as guarantor. TL contributed to the study design. JL, NK, OR, AK, and TL contributed to the interpretation of the findings. JS, JL, NK, OR, AK, and TL critically reviewed the manuscript and approved the final version of the manuscript.

Funding: JS and TL were supported by the Social Insurance Institution of Finland (grant 29/26/2020). OR was supported by the Juho Vainio Foundation (grant 202300041). AK was supported by the Economic and Social Research Council (ESRC) (grant ES/S00744X/1).

Competing interests: None declared.

Provenance and peer review: Not commissioned; externally peer reviewed.

For peer review only

Supplemental material: This content has been supplied by the authors. It has not been vetted by BMJ Publishing Group Limited (BMJ) and may not have been peer-reviewed. Any opinions or recommendations discussed are solely those of the authors and are not endorsed by BMJ. BMJ disclaims all liability and responsibility arising from any reliance placed on the content. Where the content includes any translated material, BMJ does not warrant the accuracy and reliability of the translations (including but not limited to local regulations, clinical guidelines, terminology, drug names and drug dosages), and is not responsible for any error and/or omissions arising from translation and adaptation or otherwise.

REFERENCES

1. Virtanen M, Ervasti J, Head J, et al. Lifestyle factors and risk of sickness absence from work: a multicohort study. Lancet Public Health 2018;3(11):e545–54.

2. De Bortoli MM, Oellingrath IM, Fell AKM, et al. Influence of lifestyle risk factors on work ability and sick leave in a general working population in Norway: a 5-year longitudinal study. BMJ Open 2021;11(2):e045678.

3. Laaksonen M, Piha K, Martikainen P, et al. Health-related behaviours and sickness absence from work. Occup Environ Med 2009;66(12):840–7.

4. Robroek SJW, van Lenthe FJ, Burdorf A. The role of lifestyle, health, and work in educational inequalities in sick leave and productivity loss at work. Int Arch Occup Environ Health 2013;86(6):619–27.

5. The Social Insurance Institution of Finland. Statistical Yearbook of the Social Insurance Institution 2020. Helsinki: The Social Insurance Institution of Finland 2020:181–95.

6. OECD. Sickness, Disability and Work: Breaking the Barriers: A Synthesis of Findings across OECD Countries [online]. https://www.oecd.org/publications/sickness-disability-and-work-breaking-the-barriers-9789264088856-en.htm (accessed 21 Nov 2022).

7. Kanerva N, Pietiläinen O, Lallukka T, et al. Unhealthy lifestyle and sleep problems as risk factors for increased direct employers' cost of short-term sickness absence. Scand J Work Environ Health 2018;44(2):192–201.

8. Lallukka T, Haaramo P, Rahkonen O, et al. Joint associations of sleep duration and insomnia symptoms with subsequent sickness absence: the Helsinki Health Study. Scand J Public Health 2013;41(5):516–23.

9. Amlani NM, Munir F. Does Physical Activity Have an Impact on Sickness Absence? A Review. Sports Med 2014;44(7):887–907.

10. Gohar B, Larivière M, Lightfoot N, et al. Demographic, Lifestyle, and Physical Health Predictors of Sickness Absenteeism in Nursing: A Meta-Analysis. Saf Health Work 2021;12(4):536–43.

11. Salmela J, Lahti J, Mauramo E, et al. Associations of changes in diet and leisure-time physical activity with employer's direct cost of short-term sickness absence. Eur J Sport Sci 2020;20(2):240–8.

12. Duijvenbode DC van, Hoozemans MJM, Poppel MNM van, et al. The relationship between overweight and obesity, and sick leave: a systematic review. Int J Obes 2009;33(8):807–16.

For peer review only

13. Kanerva N, Lallukka T, Rahkonen O, et al. The joint contribution of physical activity, insomnia symptoms, and smoking to the cost of short-term sickness absence. Scand J Med Sci Sports 2019;29(3):440–9.

14. Noble N, Paul C, Turon H, et al. Which modifiable health risk behaviours are related? A systematic review of the clustering of Smoking, Nutrition, Alcohol and Physical activity ('SNAP') health risk factors. Prev Med 2015;81:16–41.

15. Meader N, King K, Moe-Byrne T, et al. A systematic review on the clustering and co-occurrence of multiple risk behaviours. BMC Public Health 2016;16:657.

16. McAloney K, Graham H, Law C, et al. A scoping review of statistical approaches to the analysis of multiple health-related behaviours. Prev Med 2013;56(6):365–71.

17. Lallukka T, Pietiläinen O, Jäppinen S, et al. Factors associated with health survey response among young employees: a register-based study using online, mailed and telephone interview data collection methods. BMC Public Health 2020;20(1):184.

18. UKK Institute. Aikuisten liikkumisen suositus [online, in Finnish]. https://ukkinstituutti.fi/liikkuminen/liikkumisen-suositukset/aikuisten-liikkumisen-suositus/ (accessed 21 Nov 2022).

19. Office of Disease Prevention and Health Promotion. Physical Activity Guidelines for Americans [online]. https://health.gov/our-work/nutrition-physical-activity/physical-activity-guidelines (accessed 21 Nov 2022).

20. Current Care Guidelines. Alkoholiongelmat [online, in Finnish]. 2015. Working group set up by the Finnish Medical Society Duodecim and the Finnish Society of Addiction Medicine. Helsinki: The Finnish Medical Society Duodecim. https://www.kaypahoito.fi/hoi50028 (accessed 27 Feb 2023).

21. Laaksonen M, Mastekaasa A, Martikainen P, et al. Gender differences in sickness absence--the contribution of occupation and workplace. Scand J Work Environ Health 2010;36(5):394–403.

22. Koponen P, Borodulin K, Lundqvist A, et al (eds.). Health, functional capacity and welfare in Finland – FinHealth 2017 study. National Institute for Health and Welfare (THL). Helsinki 2018, p. 236.

23. Roelen CAM, Koopmans PC, Schreuder JAH, et al. The history of registered sickness absence predicts future sickness absence. Occup Med Oxf Engl 2011;61(2):96–101.

24. Hagenaars JA, McCutcheon AL. Applied Latent Class Analysis. Cambridge University Press 2002. p. 478.

25. Weller BE, Bowen NK, Faubert SJ. Latent Class Analysis: A Guide to Best Practice. J Black Psychol 2020;46(4):287–311.

26. Hobbs M, Duncan MJ, Collins P, et al. Clusters of health behaviours in Queensland adults are associated with different socio-demographic characteristics. J Public Health 2019;41(2):268–77.

27. Hutchesson MJ, Duncan MJ, Oftedal S, et al. Latent Class Analysis of Multiple Health Risk Behaviors among Australian University Students and Associations with Psychological Distress. Nutrients 2021;13(2):425.

28. Pettigrew S, Dana LM, McAleese A, et al. Brief Report: A Latent Class Analysis of Guideline Compliance Across Nine Health Behaviors. Int J Behav Med 2022;29(1):110–5.

For peer review only

29. Robroek SJW, van den Berg TIJ, Plat JF, et al. The role of obesity and lifestyle behaviours in a productive workforce. Occup Environ Med 2011;68(2):134–9.

30. Quist HG, Thomsen BL, Christensen U, et al. Influence of lifestyle factors on long-term sickness absence among female healthcare workers: a prospective cohort study. BMC Public Health 2014;14:1084.

31. Lallukka T, Kaikkonen R, Härkänen T, et al. Sleep and sickness absence: a nationally representative register-based follow-up study. Sleep 2014;37(9):1413–25.

32. Nielsen NBS, Gunnarsen L, Iburg KM. The impact of stress and lifestyle factors on short-term sickness absence in a large Danish industrial company. Scand J Public Health 2021;14034948211060816.

33. Kaila-Kangas L, Koskinen A, Leino-Arjas P, et al. Alcohol use and sickness absence due to all causes and mental- or musculoskeletal disorders: a nationally representative study. BMC Public Health. 2018;18(1):152.

34. Alavinia SM, van den Berg TI, van Duivenbooden C, et al. Impact of work-related factors, lifestyle, and work ability on sickness absence among Dutch construction workers. Scand J Work Environ Health 2009;35(5):325–33.

35. Sumanen H, Pietiläinen O, Lahti J, et al. Sickness absence among young employees: trends from 2002 to 2013. J Occup Health 2015;57(5):474–81.

36. Skalamera J, Hummer RA. Educational attainment and the clustering of health-related behavior among U.S. young adults. Prev Med 2016;84:83–9.

37. Sumanen H, Pietiläinen O, Lahti J, et al. Interrelationships between education, occupational class and income as determinants of sickness absence among young employees in 2002–2007 and 2008–2013. BMC Public Health 2015;15:332.

38. Mänty M, Kouvonen A, Nordquist H, et al. Physical working conditions and subsequent sickness absence: a record linkage follow-up study among 19–39-year-old municipal employees. Int Arch Occup Environ Health 2022;95(2):489–97.

39. Heinonen N, Lallukka T, Lahti J, et al. Working Conditions and Long-Term Sickness Absence Due to Mental Disorders. J Occup Environ Med 2022;64(2):105–14.

40. Burdorf A, Robroek S. Does lifestyle matter for sickness absence? Lancet Public Health. 2018;3(11):e513–4.

FIGURE LEGENDS

Figure 1. Latent classes of unhealthy behaviours among women (a) and men (b). F&V = fruit and vegetable, LTPA = leisure-time physical activity.

For peer review only

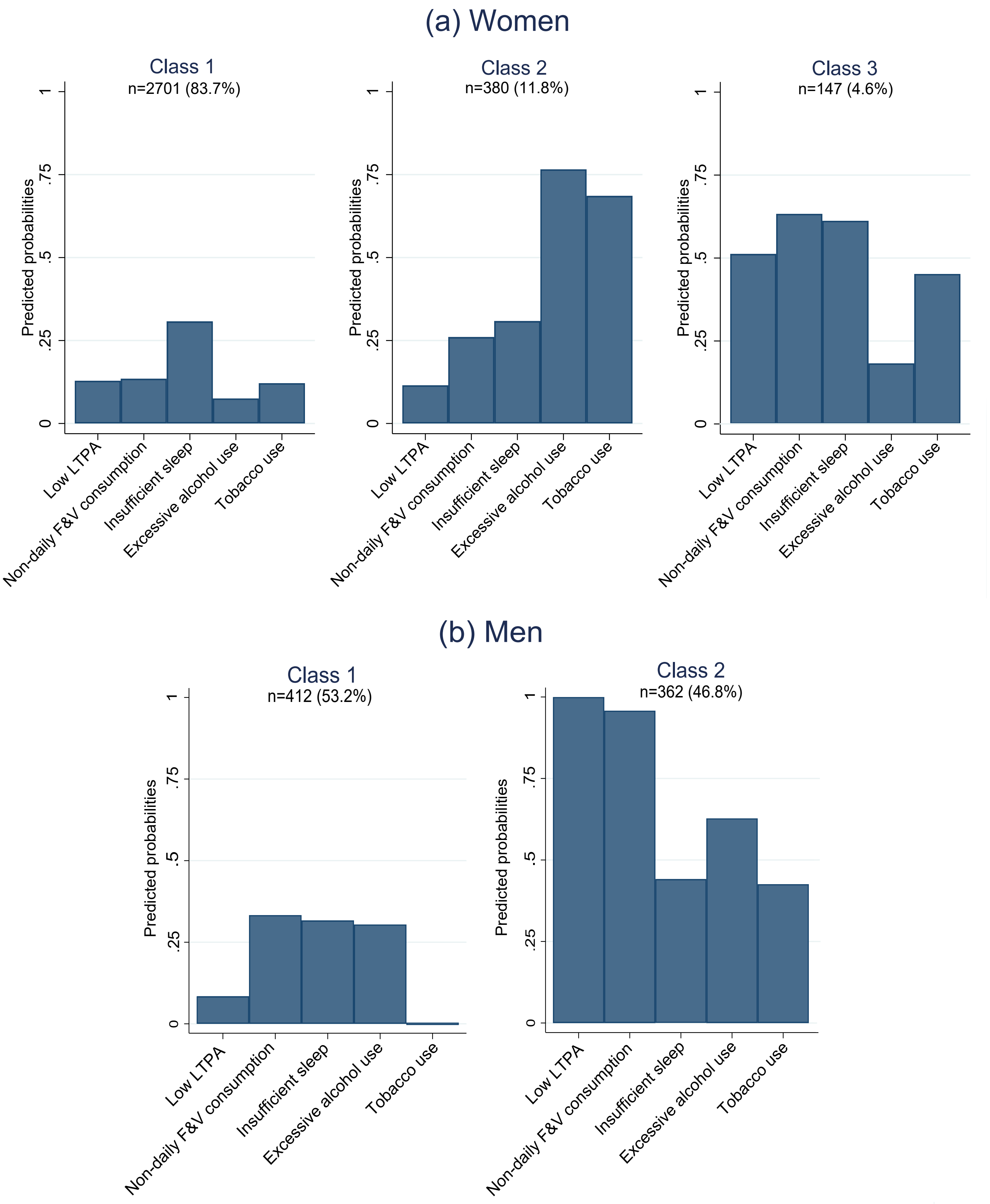
(a) Women
Class 1
n=2701 (83.7%)
Class 2
n=380 (11.8%)
Class 3
n=147 (4.6%)
Predicted probabilities
0
.25
.5
.75
1
Low LTPA
Non-daily F&V consumption
Insufficient sleep
Excessive alcohol use
Tobacco use
(b) Men
Class 1
n=412 (53.2%)
Class 2
n=362 (46.8%)

SUPPLEMENTAL FILE 1

Table S1. Model fit statistics of latent classes of unhealthy behaviours among women and men.

**Number of latent classes**	**Class membership based on posterior probabilities, n (%)**	**Marginal probabilities of class membership**	**Average posterior probabilities**	**Average posterior probabilities of class membership in each class**	**AIC value**	**BIC value**	**Entropy**
**Women**							
1	3228 (100)	1.0	1.00	1.00	16715.2	16745.6	1.00
2	2281 (70.7)	0.65	0.87	0.87	16402.5	16469.4	0.86
	947 (29.3)	0.35		0.88			
3	2701 (83.7)	0.75	0.85	0.87	16366.4	16469.8	0.86
	147 (4.6)	0.08		0.63			
	380 (11.8)	0.17		0.82			
4	1881 (58.3)	0.57	0.81	0.87	16374.7	16508.5	0.83
	781 (24.2)	0.21		0.71			
	128 (4.0)	0.07		0.66			
	438 (13.6)	0.15		0.75			
5	1628 (50.4)	0.44	0.83	0.85	16371.3	16535.4	0.90
	846 (26.2)	0.24		0.82			
	199 (6.2)	0.10		0.60			
	497 (15.4)	0.21		0.89			
	58 (1.8)	0.02		0.67			
**Men**							
1	774 (100)	1.00	1.00	1.00	4723.9	4747.2	1.00
2	412 (53.2)	0.45	0.86	0.78	4650.5	4701.7	0.88
	362 (46.8)	0.55		0.94			
3	445 (57.5)	0.58	0.96	0.99	4643.0	4722.1	0.98
	39 (5.0)	0.05		0.66			
	290 (37.5)	0.37		0.96			
4	91 (11.8)	0.22	0.76	0.84	4648.8	4751.1	0.83
	421 (54.4)	0.38		0.69			
	159 (20.5)	0.30		0.94			
	103 (13.3)	0.10		0.71			
5	20 (2.6)	0.13	0.71	0.73	4644.5	4746.8	0.90
	171 (22.1)	1.18		0.74			
	325 (42.0)	0.31		0.68			

For peer review only

	167 (21.6)	0.16	0.67
	91 (11.8)	0.22	0.85

AIC = Akaike Information Criterion, BIC = Bayesian Information Criterion

Table S2. Latent class marginal means with 95% confidence intervals (CI) for unhealthy behaviours by latent classes among women and men.

Unhealthy behaviours within latent classes	Women	Men
	Marginal mean (95% CI)	Marginal mean (95% CI)
**Class 1**		
Low LTPA	0.13 (0.11-0.15)	0.10 (0.06-0.16)
Non-daily F&V consumption	0.13 (0.11-0.16)	0.33 (0.26-0.40)
Insufficient sleep	0.31 (0.28-0.33)	0.32 (0.26-0.38)
Excessive alcohol use	0.08 (0.03-0.16)	0.21 (0.10-0.39)
Tobacco use	0.12 (0.06-0.22)	0.00 (0.00-1.00)
**Class 2**		
Low LTPA	0.11 (0.07-0.18)	0.02 (0.00-0.92)
Non-daily F&V consumption	0.26 (0.19-0.34)	0.12 (0.01-0.70)
Insufficient sleep	0.31 (0.24-0.38)	0.29 (0.21-0.38)
Excessive alcohol use	0.77 (0.17-0.98)	0.99 (0.00-1.00)
Tobacco use	0.69 (0.42-0.87)	0.51 (0.40-0.61)
**Class 3**		
Low physical activity	0.51 (0.27-0.74)	
Non-daily F&V consumption	0.63 (0.38-0.83)	
Insufficient sleep	0.61 (0.44-0.76)	
Excessive alcohol use	0.18 (0.07-0.41)	
Tobacco use	0.45 (0.28-0.63)	

F&V = fruit and vegetable, LTPA = leisure-time physical activity

For peer review only

Table S3. Sociodemographic characteristics (n, %) of latent classes of unhealthy behaviours among women and men.

Sociodemographic factors	Women				Men		
	Class 1 (n=2701)	Class 2 (n=380)	Class 3 (n=147)	P-value [a]	Class 1 (n=412)	Class 2 (n=362)	P-value [a]
**Age**				<0.001			0.098
19–29 years	827 (30.6)	176 (46.3)	46 (31.3)		93 (22.6)	104 (28.7)	
30–34 years	943 (34.9)	119 (31.3)	46 (31.3)		134 (32.5)	118 (32.6)	
35–39 years	931 (34.5)	85 (22.4)	55 (37.4)		185 (44.9)	140 (38.7)	
**Marital status**				<0.001			<0.001
Married or cohabiting	1857 (68.8)	174 (45.8)	91 (61.9)		328 (79.6)	242 (66.9)	
Other	844 (31.3)	206 (54.2)	56 (38.1)		84 (20.4)	120 (33.2)	
**Children living in the household**				<0.001			<0.001
No	1471 (54.5)	305 (80.3)	75 (51.0)		223 (54.1)	244 (67.4)	
Yes	1230 (45.5)	75 (19.7)	72 (49.0)		189 (45.9)	118 (32.6)	
**Occupational class**				<0.001			<0.001
Managers and professionals	802 (29.7)	71 (18.7)	22 (15.0)		154 (37.4)	87 (24.0)	
Semi-professionals	1167 (43.2)	173 (45.5)	62 (42.2)		129 (31.3)	113 (31.2)	
Routine non-manual workers	665 (24.6)	123 (32.4)	55 (37.4)		88 (21.4)	103 (28.5)	
Manual workers	67 (2.5)	13 (3.4)	8 (5.4)		41 (10.0)	59 (16.3)	

[a] P-values from Chi-Square Tests.

Table S4. Characteristics of all participants who responded to the Helsinki Health Study survey (2017), of those who gave their written consent to register linkages, of those who were excluded from this study [a], and of those who were finally included in this study.

	All participants who responded to the survey (n=5898)	Participants who gave their consent to register linkages (n=4864)	Excluded telephone interviewees [a] (n=651)	Other excluded participants [a] (n=211)	Participants in this study (n=4002)
**Total number of sickness absence days during the follow-up [b], median (interquartile range)**	Not available.	11 (25)	8 (24)	9 (29)	12 (26)
Health behaviours					

For peer review only

**Fresh vegetable consumption**					
Daily	4119 (70.1)	3443 (71.1)	493 (75.9)	141 (67.1)	2809 (70.5)
Non-daily	1755 (29.9)	1401 (28.9)	157 (24.2)	69 (32.9)	1175 (29.5)
**Sleep (hours)**					
7–9 hours	4573 (78.0)	3799 (78.6)	493 (75.7)	159 (76.4)	3147 (79.2)
<7 or >9 hours	1287 (22.0)	1035 (21.4)	158 (24.3)	49 (23.6)	828 (20.8)
**Alcohol use** [c]					
Once a week or less	5058 (88.9)	4153 (88.4)	610 (93.7)	179 (88.2)	3364 (87.5)
More than once a week	630 (11.1)	545 (11.6)	41 (6.3)	24 (11.8)	480 (12.5)
**Smoking** [d]					
No	4480 (76.5)	3707 (76.7)	518 (79.7)	153 (73.6)	3036 (76.4)
Currently or occasionally	1378 (23.5)	1126 (23.3)	132 (20.3)	55 (26.4)	939 (23.6)
Sociodemographic factors					
**Gender**					
Woman	4630 (78.5)	3848 (79.1)	461 (70.8)	159 (75.4)	3228 (80.7)
Man	1267 (21.5)	1016 (20.9)	190 (29.2)	52 (24.6)	774 (19.3)
**Age**					
19–29 years	1864 (31.7)	1532 (31.5)	204 (31.3)	82 (38.9)	1246 (31.1)
30–34 years	2000 (34.0)	1658 (34.1)	225 (34.6)	73 (34.6)	1360 (34.0)
35–39 years	2023 (34.4)	1674 (34.4)	222 (34.1)	56 (26.5)	1396 (34.9)
**Marital status**					
Married or cohabiting	3910 (66.3)	3248 (66.8)	427 (65.6)	129 (61.1)	2692 (67.3)
Other	1988 (33.7)	1616 (33.2)	224 (34.4)	82 (38.9)	1310 (32.7)
**Occupational class**					
Managers and professionals	1552 (27.1)	1346 (27.7)	165 (25.3)	45 (21.4)	1136 (28.4)
Semi-professionals	2233 (38.9)	1937 (39.8)	202 (31.0)	91 (43.1)	1644 (41.1)
Routine non-manual workers	1612 (28.1)	1309 (26.9)	219 (33.6)	56 (26.5)	1034 (25.8)
Manual workers	338 (5.9)	272 (5.6)	65 (10.0)	19 (9.0)	188 (4.7)

[a] Of all participants who gave their written consent to register linkages, this study excluded 1) telephone interviewees (n=651), 2) participants who had missing data on working time (n=33) after the data collection or on all health behaviours of interest (n=1), or 3) who had extreme values in health behaviours—that is, spending >24 hours together for leisure-time physical activity, sitting, and sleeping (n=166), or reporting >300 metabolic equivalent task -hours for leisure-time physical activity (n=11).

For peer review only

[b] The follow-up began from one day after receiving the completed survey questionnaire from a participant and continued until 31st March 2020 or until the end of one's employment contract, whichever came first.

[c] Participants were asked to estimate the frequency they currently use beer, wine, and spirits. The question included 10 alternatives from "never" to "daily or almost daily".

[d] Smoking included only cigarettes (not e-cigarettes or snus).

For peer review only

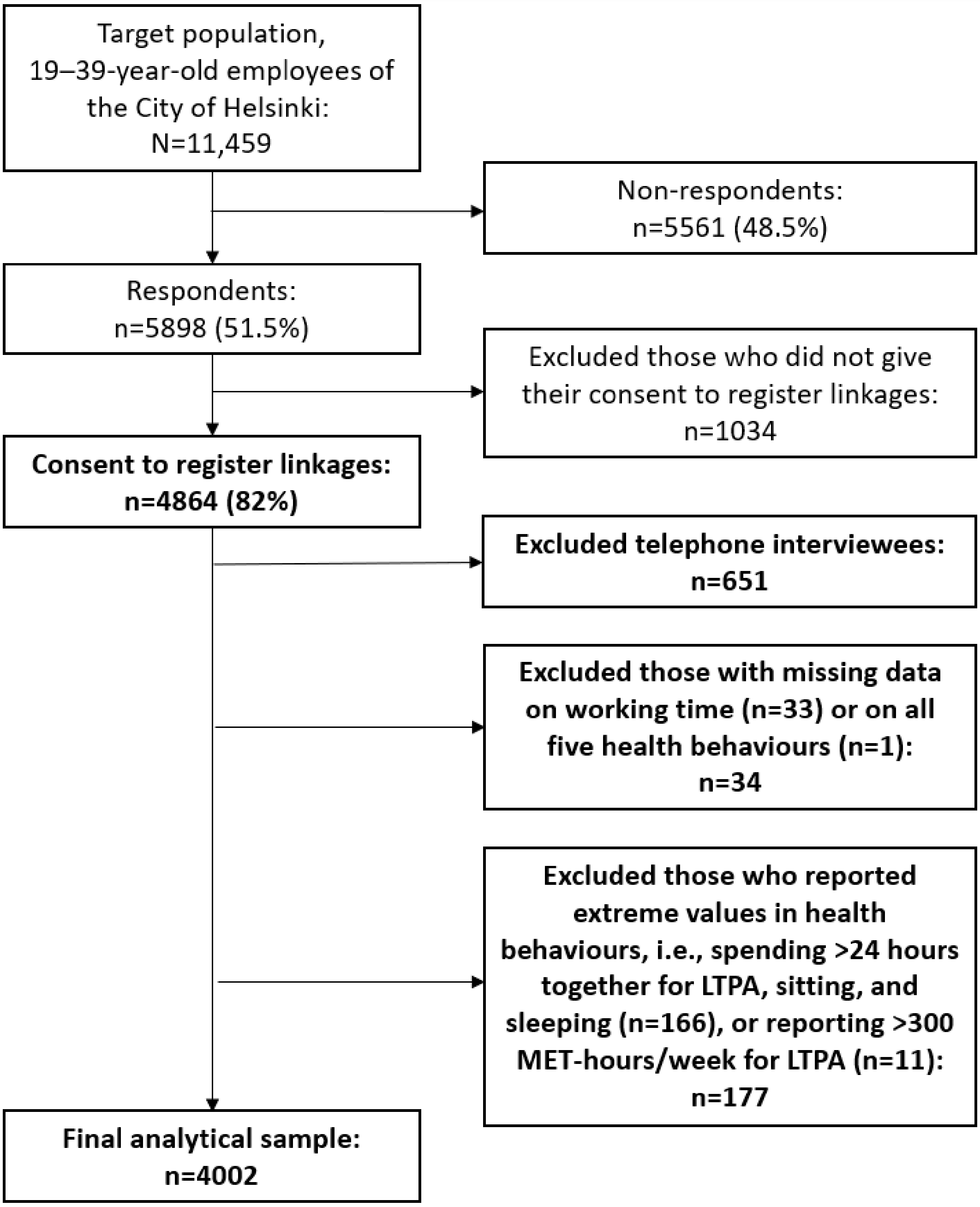

Figure S1. Flow chart: selection of the final analytical study sample.

LTPA = leisure-time physical activity, MET = metabolic equivalent task

SUPPLEMENTAL FILE 2

Helsinki Health Study survey: questions on health behaviours

Leisure-time physical activity

41. Next, we will be asking about physical activity during your leisure and commuting time over the past 12 months. We have divided physical activities in four levels of exertion. First, estimate the exertion level of the physical activities you are engaged in. Then, estimate how often you engage in a physical activity equivalent to each level of exertion during one week rounded to closest 15 minutes (e.g. 02 hours and 45 minutes).

a. During your leisure time

	Hours	Minutes
Strenuousness of exercise:		
Equivalent to walking	______	______
Equivalent to brisk walking	______	______
Equivalent to light running (jogging)	______	______
Equivalent to brisk running	______	______

b. During your commute

	Hours	Minutes
Strenuousness of exercise:		
Equivalent to walking	______	______
Equivalent to brisk walking	______	______
Equivalent to light running (jogging)	______	______
Equivalent to brisk running	______	______

Diet

39. How often do you consume the following food items? Think about the past four weeks. Please choose one alternative in each line.

	Not in the past 4 weeks	1–3 times a month	Once a week	2–4 times a week	5–6 times a week	Once a day	2 times or more a day
Dark bread (rye bread, rye crispbread, whole grain bread)	○	○	○	○	○	○	○
White bread (leavened bread, French bread etc.)	○	○	○	○	○	○	○
Sweets, chocolate	○	○	○	○	○	○	○
Sweet pastries (cookies, doughnuts, other pastries)	○	○	○	○	○	○	○
Fresh vegetables or green salad	○	○	○	○	○	○	○
Boiled vegetables	○	○	○	○	○	○	○
Vegetable dishes	○	○	○	○	○	○	○
Fruit	○	○	○	○	○	○	○
Berries	○	○	○	○	○	○	○
100 % juice	○	○	○	○	○	○	○
Fish	○	○	○	○	○	○	○
Poultry	○	○	○	○	○	○	○
Meat or processed meats (e.g. sausages)	○	○	○	○	○	○	○
Skimmed milk or fat-free dairy products	○	○	○	○	○	○	○

Sleep sufficiency

52. Do you feel that you get enough sleep?

- ○ yes, almost always
- ○ yes, often
- ○ rarely or hardly ever

Alcohol use

31. How much, on average, do you consume the following alcoholic beverage?

a. Beer or cider

- ◯ none
- ◯ less than one bottle a week
- ◯ 1–4 bottles a week
- ◯ 5–12 bottles a week
- ◯ 13–24 bottles a week
- ◯ 25–47 bottles a week
- ◯ 48 bottles or more a week

b. Wine or equivalent alcoholic beverage

- ◯ none
- ◯ less than a glass a week
- ◯ 1–4 glasses a week
- ◯ 1–2.5 bottles a week
- ◯ 3–4.5 bottles a week
- ◯ 5–9 bottles a week
- ◯ 10 bottles or more a week

c. Spirits

- ◯ not at all
- ◯ less than half a bottle a month
- ◯ 0.5–1.5 bottles a month
- ◯ 2–3.5 bottles a month
- ◯ 4–9 bottles a month
- ◯ 10–19 bottles a month
- ◯ 20 bottles or more a month

32. The next question concerns situations in which you drink six or more servings of alcoholic beverages at one sitting. Six or more servings is equivalent to at least:

- 4 pints (0.5 l each) medium-strength beer/mild cider or
- 3 pints (0.5 l each) strong beer/strong cider or
- one bottle (0.75 l) of mild wine (12%) or
- 6 restaurant servings (4 cl each) of spirits

How often do you drink six or more servings of alcoholic beverages at one sitting?

- never
- less than once a month
- once a month
- once a week
- a few times a week
- every day or almost every day

For peer review only

Smoking

28 a. Do you smoke cigarettes?

- ◯ yes, every day, how many cigarettes a day? ____________
- ◯ occasionally
- ◯ not anymore - I quit smoking in (year) ____________
- ◯ I have never smoked

28 b. Do you use snuff?

- ◯ yes, every day
- ◯ occasionally
- ◯ not anymore
- ◯ I have never used snuff

28 c. Do you use electronic cigarettes (vape)?

- ◯ yes, every day
- ◯ occasionally
- ◯ not anymore
- ◯ I have never used an electronic cigarette

STROBE Statement—checklist of items that should be included in reports of observational studies

	Item No	Recommendation	Page No
**Title and abstract**	1	(*a*) Indicate the study's design with a commonly used term in the title or the abstract	1
		(*b*) Provide in the abstract an informative and balanced summary of what was done and what was found	2
**Introduction**			
Background/rationale	2	Explain the scientific background and rationale for the investigation being reported	3
Objectives	3	State specific objectives, including any prespecified hypotheses	3
**Methods**			
Study design	4	Present key elements of study design early in the paper	3, 4
Setting	5	Describe the setting, locations, and relevant dates, including periods of recruitment, exposure, follow-up, and data collection	3-5
Participants	6	(*a*) *Cohort study*—Give the eligibility criteria, and the sources and methods of selection of participants. Describe methods of follow-up<br>*Case-control study*—Give the eligibility criteria, and the sources and methods of case ascertainment and control selection. Give the rationale for the choice of cases and controls<br>*Cross-sectional study*—Give the eligibility criteria, and the sources and methods of selection of participants	3-5
		(*b*) *Cohort study*—For matched studies, give matching criteria and number of exposed and unexposed<br>*Case-control study*—For matched studies, give matching criteria and the number of controls per case	N/A
Variables	7	Clearly define all outcomes, exposures, predictors, potential confounders, and effect modifiers. Give diagnostic criteria, if applicable	4, 5
Data sources/ measurement	8*	For each variable of interest, give sources of data and details of methods of assessment (measurement). Describe comparability of assessment methods if there is more than one group	4, 5
Bias	9	Describe any efforts to address potential sources of bias	12, 13
Study size	10	Explain how the study size was arrived at	3, 4, Figure S1
Quantitative variables	11	Explain how quantitative variables were handled in the analyses. If applicable, describe which groupings were chosen and why	4, 5
Statistical methods	12	(*a*) Describe all statistical methods, including those used to control for confounding	5, 6
		(*b*) Describe any methods used to examine subgroups and interactions	5
		(*c*) Explain how missing data were addressed	4, 13
		(*d*) *Cohort study*—If applicable, explain how loss to follow-up was addressed<br>*Case-control study*—If applicable, explain how matching of cases and controls was addressed	13

For peer review only - http://bmjopen.bmj.com/site/about/guidelines.xhtml

Cross-sectional study—If applicable, describe analytical methods taking account of sampling strategy	
(*e*) Describe any sensitivity analyses	13

Continued on next page

For peer review only

**Results**			
Participants	13*	(a) Report numbers of individuals at each stage of study—eg numbers potentially eligible, examined for eligibility, confirmed eligible, included in the study, completing follow-up, and analysed	3, 4, Figure S1
		(b) Give reasons for non-participation at each stage	3, 4, Figure S1
		(c) Consider use of a flow diagram	Figure S1
Descriptive data	14*	(a) Give characteristics of study participants (eg demographic, clinical, social) and information on exposures and potential confounders	6, 7
		(b) Indicate number of participants with missing data for each variable of interest	4, Figure S1
		(c) *Cohort study*—Summarise follow-up time (eg, average and total amount)	5
Outcome data	15*	*Cohort study*—Report numbers of outcome events or summary measures over time<br>*Case-control study*—Report numbers in each exposure category, or summary measures of exposure<br>*Cross-sectional study*—Report numbers of outcome events or summary measures	7, 8
Main results	16	(*a*) Give unadjusted estimates and, if applicable, confounder-adjusted estimates and their precision (eg, 95% confidence interval). Make clear which confounders were adjusted for and why they were included	8-10
		(*b*) Report category boundaries when continuous variables were categorized	4, 5, Tables 1 and 2
		(*c*) If relevant, consider translating estimates of relative risk into absolute risk for a meaningful time period	Tables 3 and 4
Other analyses	17	Report other analyses done—eg analyses of subgroups and interactions, and sensitivity analyses	9, 12, 13 Tables S2-4
**Discussion**			
Key results	18	Summarise key results with reference to study objectives	10
Limitations	19	Discuss limitations of the study, taking into account sources of potential bias or imprecision. Discuss both direction and magnitude of any potential bias	12, 13
Interpretation	20	Give a cautious overall interpretation of results considering objectives, limitations, multiplicity of analyses, results from similar studies, and other relevant evidence	11-13
Generalisability	21	Discuss the generalisability (external validity) of the study results	13
**Other information**			
Funding	22	Give the source of funding and the role of the funders for the present study and, if applicable, for the original study on which the present article is based	14

*Give information separately for cases and controls in case-control studies and, if applicable, for exposed and unexposed groups in cohort and cross-sectional studies.

For peer review only

Note: An Explanation and Elaboration article discusses each checklist item and gives methodological background and published examples of transparent reporting. The STROBE checklist is best used in conjunction with this article (freely available on the Web sites of PLoS Medicine at http://www.plosmedicine.org/, Annals of Internal Medicine at http://www.annals.org/, and Epidemiology at http://www.epidem.com/). Information on the STROBE Initiative is available at www.strobe-statement.org.

For peer review only

For peer review only - http://bmjopen.bmj.com/site/about/guidelines.xhtml

Latent classes of unhealthy behaviours and their associations with subsequent sickness absence: a prospective register-linkage study among Finnish young and early midlife employees

Journal:	*BMJ Open*
Manuscript ID	bmjopen-2022-070883.R2
Article Type:	Original research
Date Submitted by the Author:	21-Apr-2023

Complete List of Authors:	Salmela, Jatta; University of Helsinki Faculty of Medicine, Department of Public Health<br>Lahti, Jouni; University of Helsinki Faculty of Medicine, Department of Public Health<br>Kanerva, Noora; University of Helsinki Faculty of Agriculture and Forestry, Department of Food and Nutrition<br>Rahkonen, Ossi; University of Helsinki Faculty of Medicine, Department of Public Health<br>Kouvonen, Anne; Helsingin Yliopisto, Faculty of Social Sciences; Queen's University Belfast,<br>Lallukka, Tea; University of Helsinki Faculty of Medicine, Department of Public Health

**Primary Subject Heading**:	Occupational and environmental medicine
Secondary Subject Heading:	Public health, Epidemiology
Keywords:	PREVENTIVE MEDICINE, EPIDEMIOLOGIC STUDIES, OCCUPATIONAL & INDUSTRIAL MEDICINE, PUBLIC HEALTH

SCHOLARONE™
Manuscripts

I, the Submitting Author has the right to grant and does grant on behalf of all authors of the Work (as defined in the below author licence), an exclusive licence and/or a non-exclusive licence for contributions from authors who are: i) UK Crown employees; ii) where BMJ has agreed a CC-BY licence shall apply, and/or iii) in accordance with the terms applicable for US Federal Government officers or employees acting as part of their official duties; on a worldwide, perpetual, irrevocable, royalty-free basis to BMJ Publishing Group Ltd ("BMJ") its licensees and where the relevant Journal is co-owned by BMJ to the co-owners of the Journal, to publish the Work in this journal and any other BMJ products and to exploit all rights, as set out in our licence.

The Submitting Author accepts and understands that any supply made under these terms is made by BMJ to the Submitting Author unless you are acting as an employee on behalf of your employer or a postgraduate student of an affiliated institution which is paying any applicable article publishing charge ("APC") for Open Access articles. Where the Submitting Author wishes to make the Work available on an Open Access basis (and intends to pay the relevant APC), the terms of reuse of such Open Access shall be governed by a Creative Commons licence – details of these licences and which Creative Commons licence will apply to this Work are set out in our licence referred to above.

Other than as permitted in any relevant BMJ Author's Self Archiving Policies, I confirm this Work has not been accepted for publication elsewhere, is not being considered for publication elsewhere and does not duplicate material already published. I confirm all authors consent to publication of this Work and authorise the granting of this licence.

For peer review only

TITLE PAGE

Title of the article:

Latent classes of unhealthy behaviours and their associations with subsequent sickness absence: a prospective register-linkage study among Finnish young and early midlife employees

Authors:

Jatta Salmela [1]*, Jouni Lahti [1], Noora Kanerva [2], Ossi Rahkonen [1], Anne Kouvonen [3,4], Tea Lallukka [1]

Affiliations:

[1] Department of Public Health, University of Helsinki, Helsinki, Finland

[2] Department of Food and Nutrition, University of Helsinki, Helsinki, Finland

[3] Faculty of Social Sciences, University of Helsinki, Helsinki, Finland

[4] Centre for Public Health, Queen's University Belfast, Belfast, Northern Ireland

***Corresponding author:**

Jatta Salmela, PhD

Postal address:

Department of Public Health

PO BOX 20 (Tukholmankatu 8 B)

00014 University of Helsinki, Finland

email: jatta.salmela@helsinki.fi

Word count, excluding title page, abstract, references, figures, and tables:

3753

Keywords:

Employment, Health Behavior, Latent Class Analysis, Sick Leave

For peer review only

ABSTRACT

Objectives: Unhealthy behaviours are associated with increased sickness absence (SA), but few studies have considered person-oriented approach in these associations. Using latent class analysis, we examined clustering of unhealthy behaviours among Finnish municipal employees and their associations with subsequent SA.

Design: A prospective register-linkage study.

Setting: Unhealthy behaviours (low leisure-time physical activity, non-daily fruit and vegetable consumption, insufficient sleep, excessive alcohol use, and tobacco use) were derived from the Helsinki Health Study questionnaire survey, collected in 2017 among 19–39-year-old employees of the City of Helsinki, Finland.

Participants: A total of 4002 employees (81% women) of the City of Helsinki, Finland.

Primary outcome measures: The questionnaire data were prospectively linked to employer's SA register through March 2020. Associations between latent classes of unhealthy behaviours and subsequent SA (1–7 days / 8+ days / all lengths) were examined using negative binomial regression.

Results: Among women, a 3-class latent class model was selected: 1) few unhealthy behaviours (84%), 2) excessive alcohol and tobacco use (12%), and 3) several unhealthy behaviours (5%). Women belonging to Classes 2 and 3 had increased SA rates compared to those in Class 1, regardless of the length of SA spells. Among men, a 2-latent class model was selected: 1) few unhealthy behaviours (53%) and 2) several unhealthy behaviours (47%). Men belonging to Class 2 had increased rates of 1–7 days' SA compared to men in Class 1.

Conclusions: This study suggests that preventive actions aiming to reduce employees' SA should consider simultaneously several unhealthy behaviours. Targeted interventions may benefit of identifying the clustering of these behaviours among occupational groups.

STRENGTHS AND LIMITATIONS OF THIS STUDY

- Applying person-oriented approach enabled us to identify unobserved population groups that share similar patterns of unhealthy behaviours.
- We could link questionnaire data on employees' health behaviours to employers' register data on different lengths of sickness absence (1–7 days, 8+ days, and all lengths) with a mean follow-up time of 2.13 years.
- Self-reported measures of health behaviours may be biased, which may influence the identified latent classes of unhealthy behaviours.
- Although the large proportion of women well represents the gender distribution in the target population and in the municipal sector in Finland in general, the small number of men limits the interpretation of the findings among men and the gender comparisons.

For peer review only

INTRODUCTION

Health behaviours have a major contribution to employees' sickness absence (SA). It is estimated that 15–31% of SA could be attributed to unhealthy behaviours(1,2). In addition to their independent contributions, health behaviours can mediate some of the effects of working conditions and socioeconomic circumstances on SA: for instance, unhealthy behaviours (e.g., smoking and binge drinking) may be used to cope with stressful working conditions(3,4). In Finland, as in most high-income countries, the leading causes for medically certified SA are mental and musculoskeletal disorders(5,6). Unhealthy behaviours, such as low physical activity, poor sleep, binge drinking, and smoking, have been associated with both medically certified SA and self-certified SA(1,7–9). However, the results are not fully consistent(2,10). Diet appears to have a minor contribution to SA(2,3,7,11), but since obesity is consistently associated with SA(12), dietary aspects—which play a key role in weight management—should not be neglected. The possible mechanisms and pathways between unhealthy behaviours and SA have been suggested to proceed, for instance, through increased risk for chronic diseases, risk-taking lifestyle, and decreased immune system (leading to, e.g., common cold)(1–3). Additionally, working conditions and socioeconomic circumstances may explain some of the associations(3).

Accumulation of several unhealthy behaviours have been shown to increase SA more than individual unhealthy behaviours(2,7,13). Our previous study on midlife and ageing Finnish employees found that the joint contribution of physical inactivity and smoking was especially detrimental for employer's cost of 1–14 days' SA(13). Health behaviours tend to be clustered within population groups(14,15), and these clusters may have synergistic effects on health(14). Considering clustering of unhealthy behaviours can help policymakers and researchers to design targeted interventions to improve employees' health behaviours and reduce SA. However, to best of our knowledge, no studies have examined how clustering of unhealthy behaviours is associated with SA. Clustering techniques, such as latent class analyses, can provide more holistic approach on how health behaviours contribute to SA compared to summary indices(2,7) that consider each risk factor equally and disregard their interconnections(16).

This study aimed to identify latent classes of five unhealthy behaviours among 19–39-year-old employees of the City of Helsinki, Finland. Furthermore, using linkage to employer's SA register, we aimed to examine the associations between the latent classes with subsequent SA.

METHODS

Data and study population

This study is a part of the Helsinki Health Study of young and early midlife employees of the City of Helsinki(17). The City of Helsinki is the largest employer in Finland with around 38,000 employees and hundreds of occupational titles. The target population included 11,459 employees who were born in 1978 or later, who had a job contract of at least 50% of regular work hours per week, and whose employment contract had lasted at least 4 months before the data collection began in autumn 2017. Data were collected via online

For peer review only

and mailed questionnaires, which included a large variety of questions related to participants' social and economic characteristics and health behaviours. Additionally, shorter telephone interviews were conducted to target those who did not respond online or via mail. The overall response rate was 51.5% (n=5898)(17). The survey data were linked to employer's personnel register data for those who gave their written informed consent (82% of respondents, n=4864). We excluded telephone interviewees (n=651) since the interviews did not include all the variables of interest in this study, as well as participants who had missing data on working time or on all health behaviours of interest (n=34), or who had extreme values in health behaviours (n=177) (online supplemental file 1, figure S1). The final analytical sample included 4002 participants (81% women).

Health behaviour measures

We included five unhealthy behaviours from the survey: 1) low leisure-time physical activity (LTPA), 2) non-daily fruit and vegetable (F&V) consumption, 3) insufficient sleep, 4) excessive alcohol use, and 5) tobacco use (see online supplemental file 2). Since it is not computationally possible to include too many multi-categorical variables or variables with very small group sizes in the LCA models, we dichotomised all health behaviour measures taking into consideration current guidelines and group sizes in the variables. Participants were inquired about their weekly volume and intensity of exercise in their leisure time or while commuting during the past 12 months. Four levels of intensity were provided, and they were multiplied by the time used per week in LTPA, yielding weekly metabolic equivalent task (MET) -hours(7). Then, we dichotomised participants to those with high/moderate LTPA and those with low LTPA by using a cut-point of 20 MET-hours. Twenty MET-hours equals, for instance, 2.5 hours brisk walking and 1.5 hours walking, which was considered closely to correspond current guidelines(18,19).

For peer review only

F&V consumption during the past 4 weeks was inquired using a 14-item food frequency questionnaire. We dichotomised participants into daily (once a day or more F or V) and non-daily F&V consumers. Subjective experience of sleep was used as a sleep measure. We dichotomised participants into those who estimated that they sleep always/often sufficiently and those who estimated that they sleep seldom/never sufficiently. Alcohol use combined the measures of total weekly alcohol use and binge drinking behaviour. Weekly alcohol use was calculated based on participants' estimation on how often they consume different alcohol types (beer/cider, wine, and spirits). Seven frequency alternatives were provided for each question, with one unit of alcohol equalling 12g ethanol. Based on the Finnish Current Care Guidelines on alcohol consumption(20), 7 weekly units for women and 14 weekly units for men (i.e., moderate risk levels) were considered as cut-points. Additionally, participants were asked how often they drink six units or more at once (six response alternatives). We dichotomised those drinking less than 7/14 (women/men) units per week and binge drinking less than once a month into moderate alcohol users, and others to excessive alcohol users. We merged those not drinking alcohol at all (4% of women and 2% of men) with moderate alcohol users since their associations with SA did not differ from those drinking moderately alcohol. Participants were provided four alternatives to estimate their use of tobacco products (cigarettes, e-cigarettes, and snus): 'yes, daily', 'sporadically', 'not nowadays',

and 'never'. We dichotomised participants into never-/ex-users, and those using daily/occasionally tobacco products.

Sickness absence measures

The data on SA were derived from the personnel register of the City of Helsinki. The follow-up of SA began from one day after receiving the completed survey questionnaire and continued until 31st March 2020 or until the end of one's employment contract, whichever came first. The time limit was selected so that we could exclude the potential influence of the COVID-19 pandemic to the results. The mean follow-up time was 2.13 years. We combined overlapping and consecutive SA spells and divided them into SA spells of 1–7 days and 8+ days. During the follow-up, the City of Helsinki had a policy that 1–7 days' SA could be given to an employee by their supervisor, nurse, occupational physiotherapist, or physician, whereas 8+ days' SA required a medical certification approved by a physician. The policy was the same for all employees. Additionally, we analysed all lengths' SA.

Covariates

We stratified all analyses by gender (woman/man), given that notable gender differences have been observed in SA and health behaviours(21,22), and clustering of health behaviours may vary by gender(15). Age included categories of 19–29, 30–34, and 35–39 years. Marital status was derived from the questionnaire and was dichotomised into married/cohabiting and other. In the questionnaire, participants were inquired whether they had any 0–18-year-old children living in their household ('yes/no'). Occupational class was derived from the employer's personnel register for those who gave their informed consent for register linkage (82%), and for others, the information was derived from the questionnaire. Occupational class included four groups: managers and professionals (e.g., teachers and physicians), semi-professionals (e.g., nurses and foremen), routine non-manual workers (e.g., childcare and elderly care workers), and manual workers (e.g., care assistants). It is noteworthy that in recent years the City of Helsinki has outsourced most of their manual work (e.g., cleaning and transport work), and therefore the proportion of manual workers employed by the city is now very low. Prior SA, especially past year's SA, is known to predict future SA(10,23). Thus, we included prior SA of any length during one year before participant's response to the questionnaire.

Statistical methods

We first tabulated descriptive statistics by key exposure variables. Then, incidence of SA days per 10 person-years were calculated by individual health behaviours using negative binomial regression. We identified latent classes of unhealthy behaviours using latent class analysis (LCA). LCA is a person-oriented statistical procedure to detect latent (unobserved) subgroups, which share certain outward characteristics, within a heterogeneous population(24,25). This subtype of structural equation modelling uses categorical indicator variables to form latent classes based on the indicator variables. Participants are assigned to the latent classes based on their probability of class membership. We used the following statistical criteria for selecting the most

For peer review only

optimal number of latent classes: Bayesian information criterion (BIC), Akaike information criterion (AIC), average posterior probabilities of class membership (>0.8), class sizes (>50 cases or >5% of the sample), and entropy (>0.8)(25). One- to five-class models were run, and the model fit evaluation process is shown in online supplemental file 1, table S1. Additionally, we considered the interpretability of the models to select the final models(25).

We used negative binomial regression to examine associations between latent classes of unhealthy behaviours and subsequent SA due to overdispersion in the data. Rate ratios (RRs) and predictive margins with 95% confidence intervals (CIs) were calculated. Model 1 was adjusted for age, and model 2 further for marital status, children living in the household, occupational class, and prior SA. Natural logarithm of the follow-up time was included as an offset variable in all models to consider differences in the follow-up times between participants. All analyses were performed using STATA version 17.0 (StataCorp LLC, College Station, TX, USA).

Patient and public involvement

Patients or the public were not involved in this study.

RESULTS

Characteristics of study population

Most participants had at least one unhealthy behaviour (67% of women and 83% of men), whereas under 1% of women and men had all five unhealthy behaviours. Low LTPA and insufficient sleep were equally common among women and men (table 1). However, non-daily F&V consumption, excessive alcohol use, and tobacco use were more common among men than among women. Most women and men were married/cohabiting and around 40% had children living in their household. Only 3% of women were manual workers while the corresponding proportion for men was 13%.

Table 1. Characteristics of the participants by sociodemographic factors and health behaviours among women and men.

**Total**	**Women (n, %)**	**Men (n, %)**
	3228 (80.7)	774 (19.3)
Health behaviours		
**Leisure-time physical activity [a]**		
High or moderate activity	2689 (84.4)	651 (85.3)
Low activity	499 (15.7)	112 (14.7)
**Fruit and vegetable consumption**		
Daily	2595 (80.5)	463 (60.0)
Non-daily	629 (19.5)	309 (40.0)
**Sleep sufficiency**		
Mostly sufficient sleep	2146 (66.9)	521 (67.8)
Insufficient sleep	1064 (33.2)	248 (32.3)
**Alcohol use [b]**		

For peer review only

Moderate	2492 (79.9)	423 (55.8)
Excessive	626 (20.1)	335 (44.2)
**Tobacco use** [c]		
No	2430 (75.8)	471 (61.1)
Currently or occasionally	777 (24.2)	300 (38.9)
Sociodemographic factors		
**Age**		
19–29 years	1049 (32.5)	197 (25.5)
30–34 years	1108 (34.3)	252 (32.6)
35–39 years	1071 (33.2)	325 (42.0)
**Marital status**		
Married or cohabiting	2122 (65.7)	570 (73.6)
Other	1106 (34.3)	204 (26.4)
**Children living in the household**		
No	1851 (57.3)	467 (60.3)
Yes	1377 (42.7)	307 (39.7)
**Occupational class**		
Managers and professionals	895 (27.7)	241 (31.1)
Semi-professionals	1402 (43.4)	242 (31.3)
Routine non-manual workers	843 (26.1)	191 (24.7)
Manual workers	88 (2.7)	100 (12.9)

[a] Leisure-time physical activity (LTPA) included physical activity during leisure time and active commuting. High or moderate LTPA was considered as ≥20 metabolic equivalent task (MET) -hours per week and low LTPA as <20 MET-hours per week.

[b] Moderate alcohol use: ≤7 units of alcohol per month and binge drinking less than once a month among women, and ≤14 units of alcohol per month and binge drinking less than once a month among men. Excessive alcohol use: >7 units of alcohol per month and binge drinking less than once a month among women, and >14 units of alcohol per month and binge drinking less than once a month among men.

[c] Tobacco use included use of cigarettes, e-cigarettes, and snus.

During the follow-up, we recorded altogether 117 SA days/10 person-years for women and 93 SA days/10 person-years for men. Of women, 15% had no 1–7 days' SA, 69% had no 8+ days' SA, and 18% had no SA of any length during the follow-up. For men, the corresponding figures were 18%, 75% and 17%. Participants with healthier behaviours had less SA than those with unhealthier behaviours in general (table 2). However, F&V consumption and alcohol use were exceptions among men in terms of 8+ days' SA: those with healthier behaviour had more or equally 8+ days' SA compared to those with unhealthier behaviour. When scrutinising all lengths' SA, the greatest differences between healthy and unhealthy behaviour groups were seen in tobacco use among women and in sleep among men.

For peer review only

Table 2. Incidence of sickness absence days per 10 person-years with 95% confidence intervals (in parenthesis), by health behaviours among women and men.

**Health behaviours**	**Women (n=3228)**			**Men (n=774)**		
	**1–7 days' SA**	**8+ days' SA**	**All lengths' SA**	**1–7 days' SA**	**8+ days' SA**	**All lengths' SA**
**Leisure-time physical activity** [a]						
High or moderate activity	62 (59-64)	67 (58-77)	128 (122-135)	50 (46-55)	40 (30-55)	90 (81-101)
Low activity	72 (66-80)	74 (55-99)	146 (130-163)	64 (51-81)	52 (26-104)	116 (89-152)
**Fruit and vegetable consumption**						
Daily	61 (58-63)	67 (58-78)	127 (121-134)	47 (42-52)	45 (31-65)	92 (81-105)
Non-daily	74 (68-81)	74 (57-96)	148 (133-163)	59 (52-68)	36 (24-56)	96 (83-112)
**Sleep sufficiency**						
Mostly sufficient sleep	61 (58-64)	59 (51-70)	120 (113-127)	46 (41-51)	32 (22-46)	77 (69-88)
Insufficient sleep	68 (63-73)	88 (72-108)	155 (143-169)	64 (56-74)	62 (40-95)	127 (108-149)
**Alcohol use** [b]						
Moderate	60 (58-63)	66 (57-76)	125 (119-132)	49 (44-55)	42 (29-61)	91 (80-104)
Excessive	75 (69-82)	81 (62-105)	156 (140-172)	56 (50-64)	42 (27-66)	99 (85-115)
**Tobacco use** [c]						
No	58 (56-61)	62 (53-72)	119 (113-126)	48 (43-53)	37 (26-53)	84 (74-96)
Currently or occasionally	79 (74-85)	91 (72-114)	169 (155-185)	59 (52-67)	49 (32-77)	108 (93-126)

[a] Leisure-time physical activity (LTPA) included physical activity during leisure time and active commuting. High or moderate LTPA was considered as ≥20 metabolic equivalent task (MET) - hours per week and low LTPA as <20 MET-hours per week.

[b] Moderate alcohol use: ≤7 units of alcohol per month and binge drinking less than once a month among women, and ≤14 units of alcohol per month and binge drinking less than once a month among men. Excessive alcohol use: >7 units of alcohol per month and binge drinking less than once a month among women, and >14 units of alcohol per month and binge drinking less than once a month among men.

[c] Tobacco use included use of cigarettes, e-cigarettes, and snus.

For peer review only

Latent classes of unhealthy behaviours

The most optimal number of latent classes of unhealthy behaviours was three for women and two for men (figure 1 and online supplemental file 1, table S1). Although model fit statistics preferred the 2-class solution for women, 3 classes were selected as they were interpretatively reasonable and provided new information about the data. Most statistical criteria preferred the 3-class solution for men, but we selected 2 classes due to one too small class size (n=39) in the 3-class solution. Marginal means for each unhealthy behaviour within latent classes are shown in supplemental file 1, table S2.

[figure 1 here]

For peer review only

Of women, 84% had the highest posterior probability for belonging to Class 1, and for Classes 2 and 3, the corresponding proportions were 12% and 5% (figure 1a). Class 1 was characterised by overall low probabilities of having unhealthy behaviours. Class 2 was characterised especially by excessive alcohol use and tobacco use, whereas probabilities for other unhealthy behaviours were somewhat low. In Class 3, there were increased probabilities for all other unhealthy behaviours except excessive alcohol use. Of men, 53% had the highest posterior probability for belonging to Class 1, and 47% to Class 2 (figure 1b). Class 1 was characterised by somewhat low probabilities of having any unhealthy behaviours. The probabilities of having unhealthy behaviours were overall increased in Class 2, and it was especially characterised by low LTPA, non-daily F&V consumption, and excessive alcohol use.

Associations between latent classes of unhealthy behaviours and sickness absence

Women belonging to Classes 2 and 3 had increased SA rates compared to Class 1 (table 3). However, the associations with 8+ days' SA were not statistically significant. Women belonging to Classes 2 and 3 had increased rates of 1–7 days' SA even after adjustment for age, marital status, children living in the household, occupational class, and prior SA (table 3, M2). Men belonging to Class 2 had increased SA rates compared to Class 1 (table 4). However, statistically significant association was found only for 1–7 days' SA in the age-adjusted model (table 4, M1). This association attenuated after further adjustments (table 4, M2), especially after adjustment for occupational class.

Table 3. Associations between latent classes of unhealthy behaviours and sickness absence (SA) among women. Rate ratios (RR) and predictive margins with 95% confidence intervals (CI) from negative binomial regression models [a] are shown.

	1–7 days' SA		8+ days' SA		All lengths' SA	
**Latent class**	**M1: Adjusted for age**	**M2: M1 + marital status, children living in the household, occupational class, prior SA [b]**	**M1: Adjusted for age**	**M2: M1 + marital status, children living in the household, occupational class, prior SA [b]**	**M1: Adjusted for age**	**M2: M1 + marital status, children living in the household, occupational class, prior SA [b]**

	**RR (95 % CI)**					
**Class 1**	ref.	ref.	ref.	ref.	ref.	ref.
**Class 2**	1.39 (1.24-1.57)	1.21 (1.08-1.36)	1.37 (0.92-2.02)	1.35 (0.92-1.97)	1.39 (1.20-1.61)	1.29 (1.13-1.48)
**Class 3**	1.37 (1.14-1.64)	1.19 (1.00-1.42)	1.31 (0.72-2.38)	1.22 (0.68-2.18)	1.34 (1.07-1.67)	1.18 (0.96-1.46)
	**Predictive margins (95 % CI)**					
**Class 1**	12.6 (12.1-13.2)	12.2 (11.7-12.7)	13.7 (11.8-15.6)	10.8 (9.3-12.2)	26.2 (24.8-27.5)	23.0 (21.9-24.1)
**Class 2**	17.6 (15.6-19.6)	14.8 (13.2-16.4)	18.7 (11.8-25.5)	14.5 (9.3-19.7)	36.5 (31.5-41.5)	29.7 (25.9-33.5)
**Class 3**	17.3 (14.2-20.3)	14.5 (12.1-17.0)	17.9 (7.4-28.3)	13.1 (5.7-20.5)	35.0 (27.4-42.6)	27.2 (21.7-32.7)

[a] Natural logarithm of the follow-up time is included in the models as an offset variable.

[b] Prior sickness absence of all lengths one year before the follow-up, divided by the working time in years during the one year's period.

Table 4. Associations between latent classes of unhealthy behaviours and sickness absence (SA) among men. Rate ratios (RR) and predictive margins with 95% confidence intervals (CI) from negative binomial regression models [a] are shown.

	**1–7 SA days**		**8+ SA days**		**SA days of all lengths**	
**Latent class**	**M1: Adjusted for age**	**M2: M1 + marital status, children living in the household, occupational class, prior SA [b]**	**M1: Adjusted for age**	**M2: M1 + marital status, children living in the household, occupational class, prior SA [b]**	**M1: Adjusted for age**	**M2: M1 + marital status, children living in the household, occupational class, prior SA [b]**
	**RR (95 % CI)**					
**Class 1**	ref.	ref.	ref.	ref.	ref.	ref.
**Class 2**	1.23 (1.04-1.45)	1.11 (0.95-1.31)	1.16 (0.65-2.06)	1.01 (0.58-1.77)	1.20 (0.98-1.46)	1.06 (0.88-1.28)
	**Predictive margins (95 % CI)**					
**Class 1**	10.0 (8.8-11.1)	9.5 (8.5-10.6)	8.2 (5.0-11.4)	6.3 (4.0-8.7)	18.2 (15.7-20.7)	16.2 (14.2-18.2)
**Class 2**	12.2 (10.8-13.7)	10.6 (9.4-11.8)	9.6 (5.6-13.5)	6.4 (3.8-8.9)	21.8 (18.7-25.0)	17.2 (14.9-19.5)

[a] Natural logarithm of the follow-up time is included in the models as an offset variable.

[b] Prior sickness absence of all lengths one year before the follow-up, divided by the working time in years during the one year's period.

DISCUSSION

Summary of the main findings

By using the LCA method, we selected three latent classes of unhealthy behaviours among women, characterised as follows: 1) few unhealthy behaviours, 2) excessive alcohol use and tobacco use, and 3) several unhealthy behaviours. Among men, we selected two latent classes with the following characteristics: 1) few unhealthy behaviours and 2) several unhealthy behaviours. Women in Classes 2 and 3, and men in Class 2 had increased rates of 1–7 days' SA compared to Class 1. The associations between latent classes of unhealthy behaviours and 8+ days' SA were not statistically significant either among women or men.

For peer review only

Comparisons to the previous literature

The majority of women and men were most likely to belong to Class 1, characterised by overall healthier behaviours. Similarly, a systematic review of the clustering of smoking, nutrition, alcohol, and physical activity in adults found that a majority of included studies reported a 'healthy' cluster, characterised by the absence of any unhealthy behaviours(14). This was not affected by in how health behaviours were defined or by the used clustering analysis method(14). Some more recent studies have also identified a class of overall healthier behaviours(26–28). Additionally, previous studies have found especially alcohol consumption and smoking often clustering(14,15), which we also observed in women in Class 2. However, in men, this was not observed with two latent classes. Further analyses revealed that with a 3-class solution in men, clustering of excessive alcohol use and tobacco use existed similarly as in women. Clustering of low LTPA and non-daily F&V consumption, which we observed in Class 2 among men, has been found in many of the previous studies (14,15). However, Noble's et al. (2015) systematic review did not find clustering of physical inactivity, poor diet, and excess alcohol use—the combination that we found to reflect Class 2 in men—in any of the included studies(14). Finally, clustering of several unhealthy behaviours have been observed in many previous studies(14,26), which we also could observe in Class 3 among women and Class 2 among men.

To our knowledge, no previous studies have examined associations between latent classes of unhealthy behaviours and SA, although the relationship between health behaviours and SA are broadly studied in general. Concerning single unhealthy behaviours, previous studies have associated low LTPA(1,9,29,30), poor sleep(31,32), excessive alcohol use(1,33), and smoking(1,2,29,30,32,34) with SA, while the contribution of poor diet to SA has been modest(2,3,7,11,29). Although diet has not been associated with SA as strongly as other health behaviours, we found that inadequate F&V consumption was one major characteristic of Class 3 among women and Class 2 among men—the classes that were associated with increased subsequent SA. Our previous study on midlife and older employees also showed that the joint contribution of F&V consumption and LTPA to SA might be stronger than the individual contribution of LTPA(11). However, since F&V consumption reflects only partially participants' overall diet, further studies that consider dietary patterns more comprehensively are needed.

Our previous study showed that midlife and older employees with three or more unhealthy behaviours had higher cost of 1–14 days' SA than employees without any unhealthy behaviours(7). In particular, low LTPA, poor sleep, and current smoking increased the SA cost(7). Another study by our research group found that the joint contributions of low LTPA, poor sleep, and smoking to 1–14 days' SA cost were stronger than the contributions of these health behaviours individually(13). A Norwegian study on general working population found that an exposure to multiple health-related risk factors (low physical activity, unhealthy diet, obesity, and current smoking) was associated with increased subsequent 1–14 days' and 15+ days' SA(2). Additionally, a Danish study on private sector employees found that exposure to multiple health-related risk factors (dyssomnia, overweight, unhealthy food habits, smoking, excessive alcohol use, and low physical activity)

For peer review only

were related to increased 1–14 days' SA(32). These previous findings are concordant with our finding which indicated that the latent classes of several unhealthy behaviours (Class 3 for women and Class 2 for men) were related to increased SA rates.

We found that latent classes of unhealthy behaviours were associated with 1–7 days' SA among women and men, but not statistically significantly with 8+ days' SA. In contrast, previous studies have found stronger associations for longer SA spells(2,3). There is some evidence that younger employees have more often short-term SA and older employees long-term SA(34,35), which may partly explain our findings. Another explanation is that the follow-up period of 2.13 years may not be long enough to ensure the associations with 8+ days' SA since their rate during the follow-up was relatively low.

Previous research has shown that clustering of unhealthy behaviours is strongly related to socioeconomic position(14,15,26,27,36). Similarly, we found that managers and professionals were more likely to belong to the 'healthiest' latent classes (online supplemental file 1, table S3). However, occupational class together with other sociodemographic factors explained only some of the associations between the latent classes of unhealthy behaviours and SA. Since socioeconomic differences in SA are visible already among young employees(37) and employees in the lower socioeconomic positions are more likely to have adverse working conditions (e.g., higher exposure to physical workload) that are strongly related to increased SA(29,34,38,39), these factors should not be neglected when designing targeted health behaviour interventions at workplaces. Burdorf and Robroek (2018) have suggested that preventive interventions should simultaneously consider improvements in working conditions and health behaviours, and they should be targeted to high-risk and low-educated population groups(40). These could include, for example, reducing physical and psychosocial strenuousness of work while making healthy choices more easily available, for instance, by supporting active commuting, providing exercise facilities, improving availability of staff canteens providing healthy meals, and improving accessibility to occupational health services. Identifying occupational groups among whom these conditions are insufficient and among whom unhealthy behaviours are common is crucial for employers. Additionally, given that younger age predisposes to clustering of unhealthy behaviours(online supplemental file 1, table S3,(14,26,27)—and that health behaviours may be more difficult to modify the older individuals are—preventive actions are especially needed among young employees in the lower socioeconomic positions.

Limitations and strengths

This study has a few limitations that should be considered. First, health behaviours were self-reported, thus biased estimates are possible. Second, the used cut-points in the health behaviour measures may have affected the identified latent classes. We tested various options and made the final decisions of the dichotomisations based on their consistency with the current guidelines and their proportions in the data. Third, the used cut-point in SA (1–7/8 days) complicates the comparisons to other studies since many previous studies have used cut-points of 3/4 days or 14/15 days to distinguish short-term SA from long-term SA. However, 15+ days' SA

For peer review only

were rare in this study population, and the changes made in the SA practices by the City of Helsinki during the follow-up period supported using the chosen cut-point. SA spells of 8+ days were still relatively rare in the study population, and a longer follow-up period could have strengthened the interpretation of the findings. Fourth, the small number of men limits the interpretation of the findings among men and the gender comparisons. The large proportion of women well represents, however, the gender distribution in the target population and in the municipal sector in Finland in general.

Fifth, missing data and non-participation may affect the findings. LCA uses maximum likelihood estimation and assumes missingness at random(25), thus missing data on health behaviours were allowed. However, we have carefully examined the representativeness of the data and found them to satisfactorily represent the target population (N=11,459)(17). The response rate to the survey was moderate (51.5%), and the non-respondents were somewhat more often men, manual workers, had lower income, and had more 15+ days' SA(17). Additionally, the participants included in this current study were more often of higher occupational class (online supplemental file 1, table S4); thus, our results may be slightly conservative(26,36). However, the sensitivity analyses showed that the final analytical sample (n=4002) highly resembled the full sample (n=5898) in terms of health behaviours and socioeconomic characteristics (online supplemental file 1, table S4). In addition to the use of comprehensive survey data, a further strength of this study is that we could link the questionnaire survey to employer's SA registers, which is rarely possible. Furthermore, using the person-oriented LCA method to deepen our understanding on the associations between unhealthy behaviours and SA is a novel approach in this study area.

CONCLUSIONS

This study identified three latent classes of unhealthy behaviours for women and two for men. The 'healthiest' classes among women and men showed the lowest SA rates. The associations of the latent classes of unhealthy behaviours were stronger with 1–7 days' than with 8+ days' SA. Thus, by considering the clustering of unhealthy behaviours among young and early midlife employees and intervening in them may reduce employees' short-term SA at least. Occupational class together with other sociodemographic factors explained some of the found associations, thus special focus on employees with lower occupational positions is needed.

DATA AVAILABILITY STATEMENT

Data are available upon reasonable request. The Helsinki Health Study survey data cannot be made publicly available due to strict data protection laws and regulations. The data can only be used for scientific research and to the research group's cooperation partners with a reasonable request and study plan. More information on the availability of the survey data can be inquired from the Helsinki Health Study research group (kttl-hhs@helsinki.fi). Register data cannot be shared.

For peer review only

ETHICS STATEMENTS

Patient consent for publication

Not applicable. All participants have been informed about their rights and other ethical considerations (e.g., no participant can be identified from published results, voluntary participation, possibility to withdraw from the study, and how and where the data are used) prior to their inclusion in the study.

Ethics approval

The Helsinki Health Study protocol has been approved by the ethics committees of Department of Public Health at the University of Helsinki (30.11.1998) and the health authorities of the City of Helsinki (5.10.1999). The permission to have access to the employer's personnel register data was obtained from the City of Helsinki. Department of Public Health gave an approval (positive statement) for the study, and because the study is observational, ethical approval was not required. The City of Helsinki admitted an ethical approval without a code. Appropriate ethical aspects have been followed in all phases of the study, according to the Declaration of Helsinki.

ACKNOWLEDGEMENTS

The authors thank all participating employees and the personnel administration of the City of Helsinki.

FOOTNOTES

Twitter: @JattaSalmela, @NooraKaroliinaK, @OssiRahkonen, @AKouvonen, @TeaLallukka, @HHS_helsinkiuni

Contributors: JS was the primary author of the paper, performed the statistical analyses, and is responsible for the overall content as guarantor. TL contributed to the study design. JL, NK, OR, AK, and TL contributed to the interpretation of the findings. JS, JL, NK, OR, AK, and TL critically reviewed the manuscript and approved the final version of the manuscript.

Funding: JS and TL were supported by the Social Insurance Institution of Finland (Kela) (grant 29/26/2020). OR was supported by the Juho Vainio Foundation (grant 202300041). AK was supported by the Economic and Social Research Council (ESRC) (grant ES/S00744X/1).

Competing interests: None declared.

Provenance and peer review: Not commissioned; externally peer reviewed.

For peer review only

Supplemental material: This content has been supplied by the authors. It has not been vetted by BMJ Publishing Group Limited (BMJ) and may not have been peer-reviewed. Any opinions or recommendations discussed are solely those of the authors and are not endorsed by BMJ. BMJ disclaims all liability and responsibility arising from any reliance placed on the content. Where the content includes any translated material, BMJ does not warrant the accuracy and reliability of the translations (including but not limited to local regulations, clinical guidelines, terminology, drug names and drug dosages), and is not responsible for any error and/or omissions arising from translation and adaptation or otherwise.

REFERENCES

1. Virtanen M, Ervasti J, Head J, et al. Lifestyle factors and risk of sickness absence from work: a multicohort study. Lancet Public Health 2018;3(11):e545–54.

2. De Bortoli MM, Oellingrath IM, Fell AKM, et al. Influence of lifestyle risk factors on work ability and sick leave in a general working population in Norway: a 5-year longitudinal study. BMJ Open 2021;11(2):e045678.

3. Laaksonen M, Piha K, Martikainen P, et al. Health-related behaviours and sickness absence from work. Occup Environ Med 2009;66(12):840–7.

4. Robroek SJW, van Lenthe FJ, Burdorf A. The role of lifestyle, health, and work in educational inequalities in sick leave and productivity loss at work. Int Arch Occup Environ Health 2013;86(6):619–27.

5. The Social Insurance Institution of Finland. Statistical Yearbook of the Social Insurance Institution 2020. Helsinki: The Social Insurance Institution of Finland 2020:181–95.

6. OECD. Sickness, Disability and Work: Breaking the Barriers: A Synthesis of Findings across OECD Countries [online]. https://www.oecd.org/publications/sickness-disability-and-work-breaking-the-barriers-9789264088856-en.htm (accessed 21 Nov 2022).

7. Kanerva N, Pietiläinen O, Lallukka T, et al. Unhealthy lifestyle and sleep problems as risk factors for increased direct employers' cost of short-term sickness absence. Scand J Work Environ Health 2018;44(2):192–201.

8. Lallukka T, Haaramo P, Rahkonen O, et al. Joint associations of sleep duration and insomnia symptoms with subsequent sickness absence: the Helsinki Health Study. Scand J Public Health 2013;41(5):516–23.

9. Amlani NM, Munir F. Does Physical Activity Have an Impact on Sickness Absence? A Review. Sports Med 2014;44(7):887–907.

10. Gohar B, Larivière M, Lightfoot N, et al. Demographic, Lifestyle, and Physical Health Predictors of Sickness Absenteeism in Nursing: A Meta-Analysis. Saf Health Work 2021;12(4):536–43.

11. Salmela J, Lahti J, Mauramo E, et al. Associations of changes in diet and leisure-time physical activity with employer's direct cost of short-term sickness absence. Eur J Sport Sci 2020;20(2):240–8.

12. Duijvenbode DC van, Hoozemans MJM, Poppel MNM van, et al. The relationship between overweight and obesity, and sick leave: a systematic review. Int J Obes 2009;33(8):807–16.

For peer review only

13. Kanerva N, Lallukka T, Rahkonen O, et al. The joint contribution of physical activity, insomnia symptoms, and smoking to the cost of short-term sickness absence. Scand J Med Sci Sports 2019;29(3):440–9.

14. Noble N, Paul C, Turon H, et al. Which modifiable health risk behaviours are related? A systematic review of the clustering of Smoking, Nutrition, Alcohol and Physical activity ('SNAP') health risk factors. Prev Med 2015;81:16–41.

15. Meader N, King K, Moe-Byrne T, et al. A systematic review on the clustering and co-occurrence of multiple risk behaviours. BMC Public Health 2016;16:657.

16. McAloney K, Graham H, Law C, et al. A scoping review of statistical approaches to the analysis of multiple health-related behaviours. Prev Med 2013;56(6):365–71.

17. Lallukka T, Pietiläinen O, Jäppinen S, et al. Factors associated with health survey response among young employees: a register-based study using online, mailed and telephone interview data collection methods. BMC Public Health 2020;20(1):184.

18. UKK Institute. Aikuisten liikkumisen suositus [online, in Finnish]. https://ukkinstituutti.fi/liikkuminen/liikkumisen-suositukset/aikuisten-liikkumisen-suositus/ (accessed 21 Nov 2022).

19. Office of Disease Prevention and Health Promotion. Physical Activity Guidelines for Americans [online]. https://health.gov/our-work/nutrition-physical-activity/physical-activity-guidelines (accessed 21 Nov 2022).

20. Current Care Guidelines. Alkoholiongelmat [online, in Finnish]. 2015. Working group set up by the Finnish Medical Society Duodecim and the Finnish Society of Addiction Medicine. Helsinki: The Finnish Medical Society Duodecim. https://www.kaypahoito.fi/hoi50028 (accessed 27 Feb 2023).

21. Laaksonen M, Mastekaasa A, Martikainen P, et al. Gender differences in sickness absence--the contribution of occupation and workplace. Scand J Work Environ Health 2010;36(5):394–403.

22. Koponen P, Borodulin K, Lundqvist A, et al (eds.). Health, functional capacity and welfare in Finland – FinHealth 2017 study. National Institute for Health and Welfare (THL). Helsinki 2018, p. 236.

23. Roelen CAM, Koopmans PC, Schreuder JAH, et al. The history of registered sickness absence predicts future sickness absence. Occup Med Oxf Engl 2011;61(2):96–101.

24. Hagenaars JA, McCutcheon AL. Applied Latent Class Analysis. Cambridge University Press 2002. p. 478.

25. Weller BE, Bowen NK, Faubert SJ. Latent Class Analysis: A Guide to Best Practice. J Black Psychol 2020;46(4):287–311.

26. Hobbs M, Duncan MJ, Collins P, et al. Clusters of health behaviours in Queensland adults are associated with different socio-demographic characteristics. J Public Health 2019;41(2):268–77.

27. Hutchesson MJ, Duncan MJ, Oftedal S, et al. Latent Class Analysis of Multiple Health Risk Behaviors among Australian University Students and Associations with Psychological Distress. Nutrients 2021;13(2):425.

28. Pettigrew S, Dana LM, McAleese A, et al. Brief Report: A Latent Class Analysis of Guideline Compliance Across Nine Health Behaviors. Int J Behav Med 2022;29(1):110–5.

For peer review only

29. Robroek SJW, van den Berg TIJ, Plat JF, et al. The role of obesity and lifestyle behaviours in a productive workforce. Occup Environ Med 2011;68(2):134–9.

30. Quist HG, Thomsen BL, Christensen U, et al. Influence of lifestyle factors on long-term sickness absence among female healthcare workers: a prospective cohort study. BMC Public Health 2014;14:1084.

31. Lallukka T, Kaikkonen R, Härkänen T, et al. Sleep and sickness absence: a nationally representative register-based follow-up study. Sleep 2014;37(9):1413–25.

32. Nielsen NBS, Gunnarsen L, Iburg KM. The impact of stress and lifestyle factors on short-term sickness absence in a large Danish industrial company. Scand J Public Health 2021;14034948211060816.

33. Kaila-Kangas L, Koskinen A, Leino-Arjas P, et al. Alcohol use and sickness absence due to all causes and mental- or musculoskeletal disorders: a nationally representative study. BMC Public Health. 2018;18(1):152.

34. Alavinia SM, van den Berg TI, van Duivenbooden C, et al. Impact of work-related factors, lifestyle, and work ability on sickness absence among Dutch construction workers. Scand J Work Environ Health 2009;35(5):325–33.

35. Sumanen H, Pietiläinen O, Lahti J, et al. Sickness absence among young employees: trends from 2002 to 2013. J Occup Health 2015;57(5):474–81.

36. Skalamera J, Hummer RA. Educational attainment and the clustering of health-related behavior among U.S. young adults. Prev Med 2016;84:83–9.

37. Sumanen H, Pietiläinen O, Lahti J, et al. Interrelationships between education, occupational class and income as determinants of sickness absence among young employees in 2002–2007 and 2008–2013. BMC Public Health 2015;15:332.

38. Mänty M, Kouvonen A, Nordquist H, et al. Physical working conditions and subsequent sickness absence: a record linkage follow-up study among 19–39-year-old municipal employees. Int Arch Occup Environ Health 2022;95(2):489–97.

39. Heinonen N, Lallukka T, Lahti J, et al. Working Conditions and Long-Term Sickness Absence Due to Mental Disorders. J Occup Environ Med 2022;64(2):105–14.

40. Burdorf A, Robroek S. Does lifestyle matter for sickness absence? Lancet Public Health. 2018;3(11):e513–4.

For peer review only

FIGURE LEGENDS

Figure 1. Latent classes of unhealthy behaviours among women (a) and men (b). F&V = fruit and vegetable, LTPA = leisure-time physical activity.

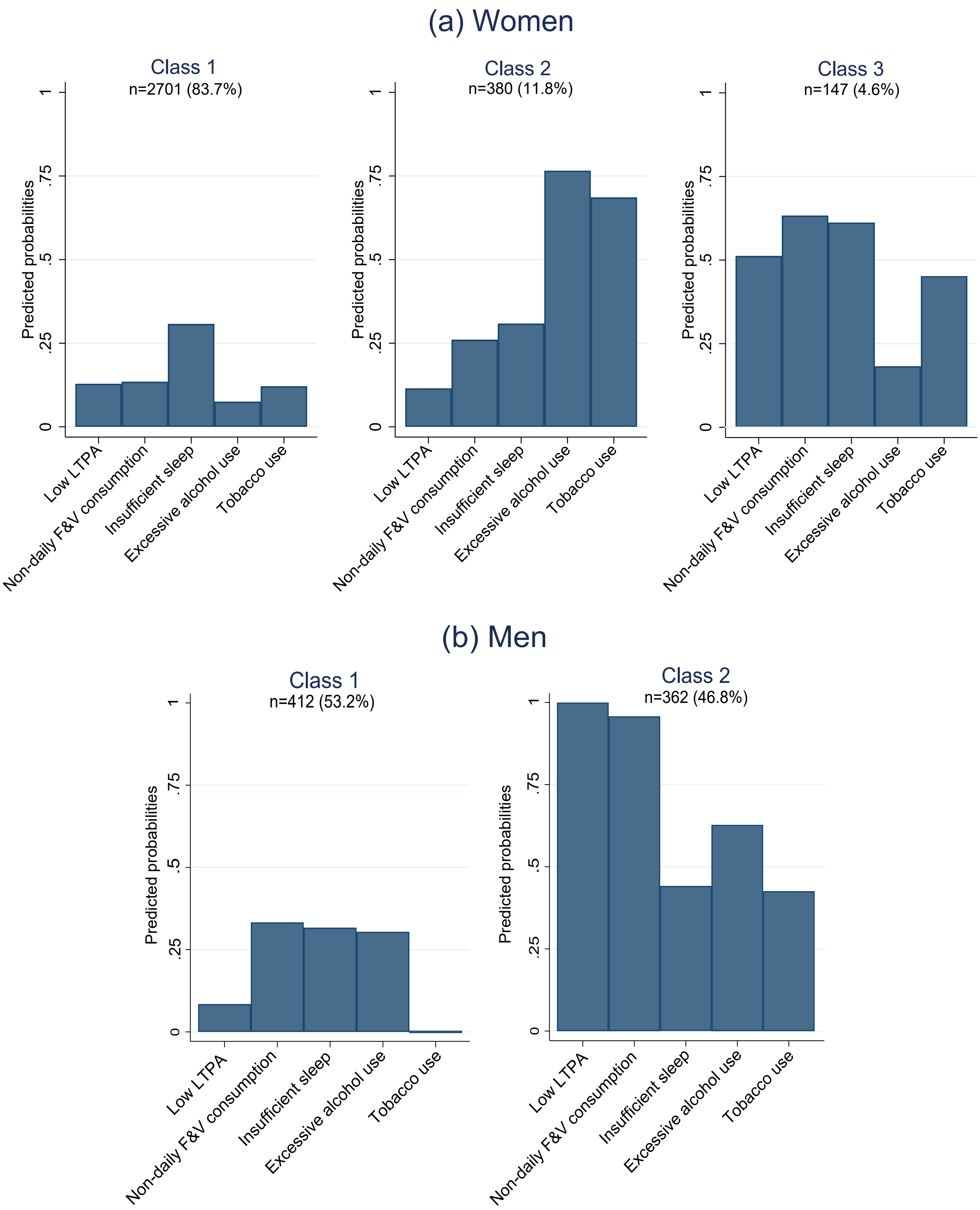
(a) Women
Class 1
n=2701 (83.7%)
Class 2
n=380 (11.8%)
Class 3
n=147 (4.6%)
Predicted probabilities
0
.25
.5
.75
1
Low LTPA
Non-daily F&V consumption
Insufficient sleep
Excessive alcohol use
Tobacco use
(b) Men
Class 1
n=412 (53.2%)
Class 2
n=362 (46.8%)

SUPPLEMENTAL FILE 1

Table S1. Model fit statistics of latent classes of unhealthy behaviours among women and men.

Number of latent classes	Class membership based on posterior probabilities, n (%)	Marginal probabilities of class membership	Average posterior probabilities	Average posterior probabilities of class membership in each class	AIC value	BIC value	Entropy
**Women**							
1	3228 (100)	1.0	1.00	1.00	16715.2	16745.6	1.00
2	2281 (70.7)	0.65	0.87	0.87	16402.5	16469.4	0.86
	947 (29.3)	0.35		0.88			
3	2701 (83.7)	0.75	0.85	0.87	16366.4	16469.8	0.86
	147 (4.6)	0.08		0.63			
	380 (11.8)	0.17		0.82			
4	1881 (58.3)	0.57	0.81	0.87	16374.7	16508.5	0.83
	781 (24.2)	0.21		0.71			
	128 (4.0)	0.07		0.66			
	438 (13.6)	0.15		0.75			
5	1628 (50.4)	0.44	0.83	0.85	16371.3	16535.4	0.90
	846 (26.2)	0.24		0.82			
	199 (6.2)	0.10		0.60			
	497 (15.4)	0.21		0.89			
	58 (1.8)	0.02		0.67			
**Men**							
1	774 (100)	1.00	1.00	1.00	4723.9	4747.2	1.00
2	412 (53.2)	0.45	0.86	0.78	4650.5	4701.7	0.88
	362 (46.8)	0.55		0.94			
3	445 (57.5)	0.58	0.96	0.99	4643.0	4722.1	0.98
	39 (5.0)	0.05		0.66			
	290 (37.5)	0.37		0.96			
4	91 (11.8)	0.22	0.76	0.84	4648.8	4751.1	0.83
	421 (54.4)	0.38		0.69			
	159 (20.5)	0.30		0.94			
	103 (13.3)	0.10		0.71			
5	20 (2.6)	0.13	0.71	0.73	4644.5	4746.8	0.90
	171 (22.1)	1.18		0.74			
	325 (42.0)	0.31		0.68			

For peer review only

	167 (21.6)	0.16	0.67
	91 (11.8)	0.22	0.85

AIC = Akaike Information Criterion, BIC = Bayesian Information Criterion

Table S2. Latent class marginal means with 95% confidence intervals (CI) for unhealthy behaviours by latent classes among women and men.

Unhealthy behaviours within latent classes	Women	Men
	Marginal mean (95% CI)	Marginal mean (95% CI)
**Class 1**		
Low LTPA	0.13 (0.11-0.15)	0.10 (0.06-0.16)
Non-daily F&V consumption	0.13 (0.11-0.16)	0.33 (0.26-0.40)
Insufficient sleep	0.31 (0.28-0.33)	0.32 (0.26-0.38)
Excessive alcohol use	0.08 (0.03-0.16)	0.21 (0.10-0.39)
Tobacco use	0.12 (0.06-0.22)	0.00 (0.00-1.00)
**Class 2**		
Low LTPA	0.11 (0.07-0.18)	0.02 (0.00-0.92)
Non-daily F&V consumption	0.26 (0.19-0.34)	0.12 (0.01-0.70)
Insufficient sleep	0.31 (0.24-0.38)	0.29 (0.21-0.38)
Excessive alcohol use	0.77 (0.17-0.98)	0.99 (0.00-1.00)
Tobacco use	0.69 (0.42-0.87)	0.51 (0.40-0.61)
**Class 3**		
Low physical activity	0.51 (0.27-0.74)	
Non-daily F&V consumption	0.63 (0.38-0.83)	
Insufficient sleep	0.61 (0.44-0.76)	
Excessive alcohol use	0.18 (0.07-0.41)	
Tobacco use	0.45 (0.28-0.63)	

F&V = fruit and vegetable, LTPA = leisure-time physical activity

For peer review only

11 **Table S3.** Sociodemographic characteristics (n, %) of latent classes of unhealthy behaviours among women and men.

Sociodemographic factors	Women				Men		
	Class 1 (n=2701)	Class 2 (n=380)	Class 3 (n=147)	P-value [a]	Class 1 (n=412)	Class 2 (n=362)	P-value [a]
**Age**				<0.001			0.098
19–29 years	827 (30.6)	176 (46.3)	46 (31.3)		93 (22.6)	104 (28.7)	
30–34 years	943 (34.9)	119 (31.3)	46 (31.3)		134 (32.5)	118 (32.6)	
35–39 years	931 (34.5)	85 (22.4)	55 (37.4)		185 (44.9)	140 (38.7)	
**Marital status**				<0.001			<0.001
Married or cohabiting	1857 (68.8)	174 (45.8)	91 (61.9)		328 (79.6)	242 (66.9)	
Other	844 (31.3)	206 (54.2)	56 (38.1)		84 (20.4)	120 (33.2)	
**Children living in the household**				<0.001			<0.001
No	1471 (54.5)	305 (80.3)	75 (51.0)		223 (54.1)	244 (67.4)	
Yes	1230 (45.5)	75 (19.7)	72 (49.0)		189 (45.9)	118 (32.6)	
**Occupational class**				<0.001			<0.001
Managers and professionals	802 (29.7)	71 (18.7)	22 (15.0)		154 (37.4)	87 (24.0)	
Semi-professionals	1167 (43.2)	173 (45.5)	62 (42.2)		129 (31.3)	113 (31.2)	
Routine non-manual workers	665 (24.6)	123 (32.4)	55 (37.4)		88 (21.4)	103 (28.5)	
Manual workers	67 (2.5)	13 (3.4)	8 (5.4)		41 (10.0)	59 (16.3)	

12 [a] P-values from Chi-Square Tests.

13

14

15 **Table S4.** Characteristics of all participants who responded to the Helsinki Health Study survey (2017), of those who gave their written consent to register
16 linkages, of those who were excluded from this study [a], and of those who were finally included in this study.

	All participants who responded to the survey (n=5898)	Participants who gave their consent to register linkages (n=4864)	Excluded telephone interviewees [a] (n=651)	Other excluded participants [a] (n=211)	Participants in this study (n=4002)
**Total number of sickness absence days during the follow-up [b], median (interquartile range)**	Not available.	11 (25)	8 (24)	9 (29)	12 (26)
Health behaviours					

For peer review only

**Fresh vegetable consumption**					
Daily	4119 (70.1)	3443 (71.1)	493 (75.9)	141 (67.1)	2809 (70.5)
Non-daily	1755 (29.9)	1401 (28.9)	157 (24.2)	69 (32.9)	1175 (29.5)
**Sleep (hours)**					
7–9 hours	4573 (78.0)	3799 (78.6)	493 (75.7)	159 (76.4)	3147 (79.2)
<7 or >9 hours	1287 (22.0)	1035 (21.4)	158 (24.3)	49 (23.6)	828 (20.8)
**Alcohol use** [c]					
Once a week or less	5058 (88.9)	4153 (88.4)	610 (93.7)	179 (88.2)	3364 (87.5)
More than once a week	630 (11.1)	545 (11.6)	41 (6.3)	24 (11.8)	480 (12.5)
**Smoking** [d]					
No	4480 (76.5)	3707 (76.7)	518 (79.7)	153 (73.6)	3036 (76.4)
Currently or occasionally	1378 (23.5)	1126 (23.3)	132 (20.3)	55 (26.4)	939 (23.6)
Sociodemographic factors					
**Gender**					
Woman	4630 (78.5)	3848 (79.1)	461 (70.8)	159 (75.4)	3228 (80.7)
Man	1267 (21.5)	1016 (20.9)	190 (29.2)	52 (24.6)	774 (19.3)
**Age**					
19–29 years	1864 (31.7)	1532 (31.5)	204 (31.3)	82 (38.9)	1246 (31.1)
30–34 years	2000 (34.0)	1658 (34.1)	225 (34.6)	73 (34.6)	1360 (34.0)
35–39 years	2023 (34.4)	1674 (34.4)	222 (34.1)	56 (26.5)	1396 (34.9)
**Marital status**					
Married or cohabiting	3910 (66.3)	3248 (66.8)	427 (65.6)	129 (61.1)	2692 (67.3)
Other	1988 (33.7)	1616 (33.2)	224 (34.4)	82 (38.9)	1310 (32.7)
**Occupational class**					
Managers and professionals	1552 (27.1)	1346 (27.7)	165 (25.3)	45 (21.4)	1136 (28.4)
Semi-professionals	2233 (38.9)	1937 (39.8)	202 (31.0)	91 (43.1)	1644 (41.1)
Routine non-manual workers	1612 (28.1)	1309 (26.9)	219 (33.6)	56 (26.5)	1034 (25.8)
Manual workers	338 (5.9)	272 (5.6)	65 (10.0)	19 (9.0)	188 (4.7)

[a] Of all participants who gave their written consent to register linkages, this study excluded 1) telephone interviewees (n=651), 2) participants who had missing data on working time (n=33) after the data collection or on all health behaviours of interest (n=1), or 3) who had extreme values in health behaviours—that is, spending >24 hours together for leisure-time physical activity, sitting, and sleeping (n=166), or reporting >300 metabolic equivalent task -hours for leisure-time physical activity (n=11).

For peer review only

[b] The follow-up began from one day after receiving the completed survey questionnaire from a participant and continued until 31st March 2020 or until the end of one's employment contract, whichever came first.

[c] Participants were asked to estimate the frequency they currently use beer, wine, and spirits. The question included 10 alternatives from "never" to "daily or almost daily".

[d] Smoking included only cigarettes (not e-cigarettes or snus).

For peer review only

For peer review only - http://bmjopen.bmj.com/site/about/guidelines.xhtml

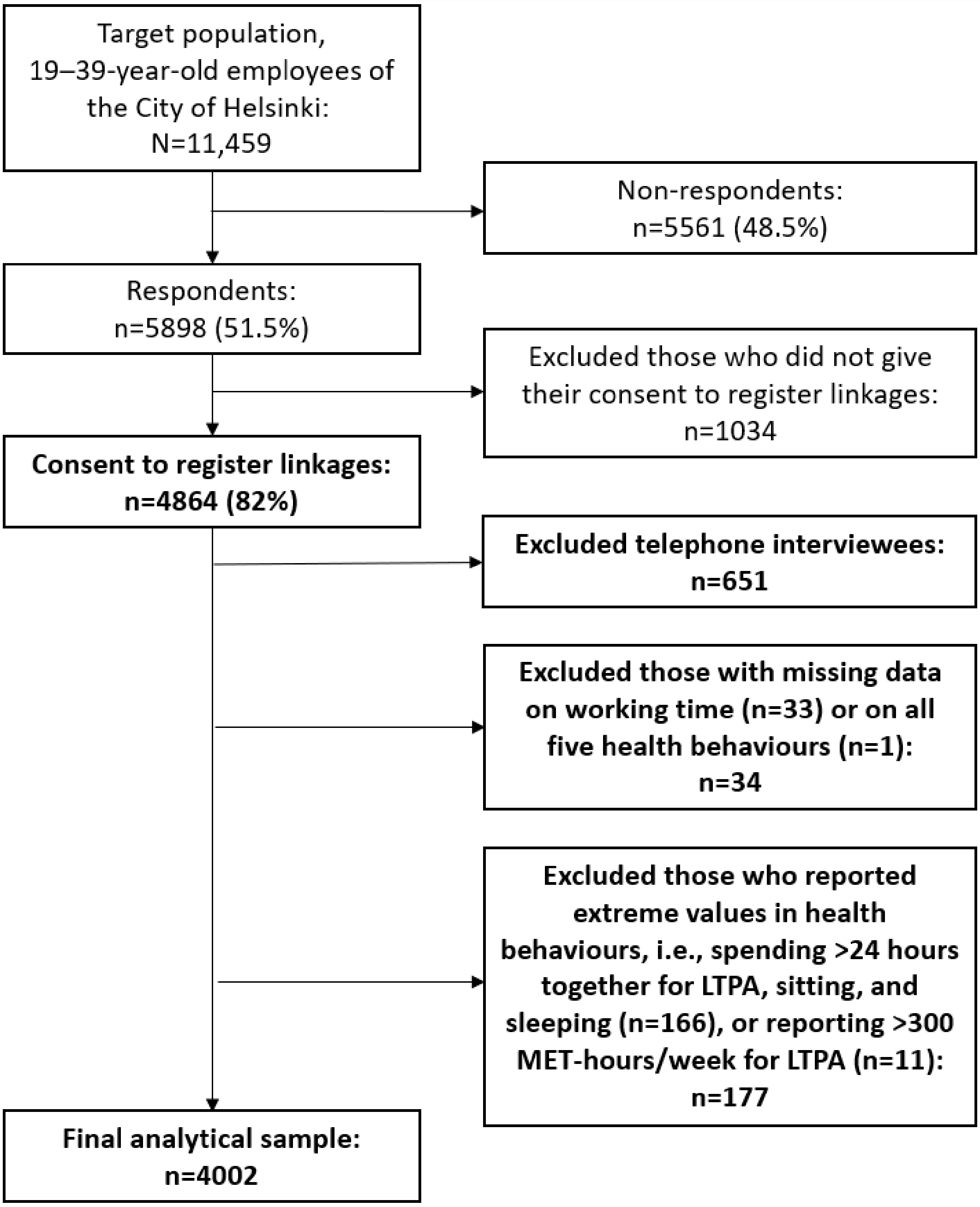

Figure S1. Flow chart: selection of the final analytical study sample.

LTPA = leisure-time physical activity, MET = metabolic equivalent task

SUPPLEMENTAL FILE 2

Helsinki Health Study survey: questions on health behaviours

Leisure-time physical activity

41. Next, we will be asking about physical activity during your leisure and commuting time <u>over the past 12 months</u>. We have divided physical activities in four levels of exertion. First, estimate the exertion level of the physical activities you are engaged in. Then, estimate how often you engage in a physical activity equivalent to each level of exertion <u>during one week</u> rounded to closest 15 minutes (e.g. 02 hours and 45 minutes).

a. During your leisure time

	Hours	Minutes
Strenuousness of exercise:		
Equivalent to walking		
Equivalent to brisk walking		
Equivalent to light running (jogging)		
Equivalent to brisk running		

b. During your commute

	Hours	Minutes
Strenuousness of exercise:		
Equivalent to walking		
Equivalent to brisk walking		
Equivalent to light running (jogging)		
Equivalent to brisk running		

Diet

39. How often do you consume the following food items? Think about the past four weeks. Please choose one alternative in each line.

	Not in the past 4 weeks	1–3 times a month	Once a week	2–4 times a week	5–6 times a week	Once a day	2 times or more a day
Dark bread (rye bread, rye crispbread, whole grain bread)	○	○	○	○	○	○	○
White bread (leavened bread, French bread etc.)	○	○	○	○	○	○	○
Sweets, chocolate	○	○	○	○	○	○	○
Sweet pastries (cookies, doughnuts, other pastries)	○	○	○	○	○	○	○
Fresh vegetables or green salad	○	○	○	○	○	○	○
Boiled vegetables	○	○	○	○	○	○	○
Vegetable dishes	○	○	○	○	○	○	○
Fruit	○	○	○	○	○	○	○
Berries	○	○	○	○	○	○	○
100 % juice	○	○	○	○	○	○	○
Fish	○	○	○	○	○	○	○
Poultry	○	○	○	○	○	○	○
Meat or processed meats (e.g. sausages)	○	○	○	○	○	○	○
Skimmed milk or fat-free dairy products	○	○	○	○	○	○	○

Sleep sufficiency

52. Do you feel that you get enough sleep?

- ○ yes, almost always
- ○ yes, often
- ○ rarely or hardly ever

Alcohol use

31. How much, on average, do you consume the following alcoholic beverage?

a. Beer or cider

- ◯ none
- ◯ less than one bottle a week
- ◯ 1–4 bottles a week
- ◯ 5–12 bottles a week
- ◯ 13–24 bottles a week
- ◯ 25–47 bottles a week
- ◯ 48 bottles or more a week

b. Wine or equivalent alcoholic beverage

- ◯ none
- ◯ less than a glass a week
- ◯ 1–4 glasses a week
- ◯ 1–2.5 bottles a week
- ◯ 3–4.5 bottles a week
- ◯ 5–9 bottles a week
- ◯ 10 bottles or more a week

c. Spirits

- ◯ not at all
- ◯ less than half a bottle a month
- ◯ 0.5–1.5 bottles a month
- ◯ 2–3.5 bottles a month
- ◯ 4–9 bottles a month
- ◯ 10–19 bottles a month
- ◯ 20 bottles or more a month

32. The next question concerns situations in which you drink six or more servings of alcoholic beverages at one sitting. Six or more servings is equivalent to at least:

- 4 pints (0.5 l each) medium-strength beer/mild cider or
- 3 pints (0.5 l each) strong beer/strong cider or
- one bottle (0.75 l) of mild wine (12%) or
- 6 restaurant servings (4 cl each) of spirits

How often do you drink six or more servings of alcoholic beverages at one sitting?

- ◯ never
- ◯ less than once a month
- ◯ once a month
- ◯ once a week
- ◯ a few times a week
- ◯ every day or almost every day

For peer review only

Smoking

28 a. Do you smoke cigarettes?

- ◯ yes, every day, how many cigarettes a day? ____________________
- ◯ occasionally
- ◯ not anymore - I quit smoking in (year) ____________________
- ◯ I have never smoked

28 b. Do you use snuff?

- ◯ yes, every day
- ◯ occasionally
- ◯ not anymore
- ◯ I have never used snuff

28 c. Do you use electronic cigarettes (vape)?

- ◯ yes, every day
- ◯ occasionally
- ◯ not anymore
- ◯ I have never used an electronic cigarette

STROBE Statement—checklist of items that should be included in reports of observational studies

	Item No	Recommendation	Page No
**Title and abstract**	1	(*a*) Indicate the study's design with a commonly used term in the title or the abstract	1
		(*b*) Provide in the abstract an informative and balanced summary of what was done and what was found	2
**Introduction**			
Background/rationale	2	Explain the scientific background and rationale for the investigation being reported	3
Objectives	3	State specific objectives, including any prespecified hypotheses	3
**Methods**			
Study design	4	Present key elements of study design early in the paper	3, 4
Setting	5	Describe the setting, locations, and relevant dates, including periods of recruitment, exposure, follow-up, and data collection	3-5
Participants	6	(*a*) *Cohort study*—Give the eligibility criteria, and the sources and methods of selection of participants. Describe methods of follow-up<br>*Case-control study*—Give the eligibility criteria, and the sources and methods of case ascertainment and control selection. Give the rationale for the choice of cases and controls<br>*Cross-sectional study*—Give the eligibility criteria, and the sources and methods of selection of participants	3-5
		(*b*) *Cohort study*—For matched studies, give matching criteria and number of exposed and unexposed<br>*Case-control study*—For matched studies, give matching criteria and the number of controls per case	N/A
Variables	7	Clearly define all outcomes, exposures, predictors, potential confounders, and effect modifiers. Give diagnostic criteria, if applicable	4, 5
Data sources/ measurement	8*	For each variable of interest, give sources of data and details of methods of assessment (measurement). Describe comparability of assessment methods if there is more than one group	4, 5
Bias	9	Describe any efforts to address potential sources of bias	12, 13
Study size	10	Explain how the study size was arrived at	3, 4, Figure S1
Quantitative variables	11	Explain how quantitative variables were handled in the analyses. If applicable, describe which groupings were chosen and why	4, 5
Statistical methods	12	(*a*) Describe all statistical methods, including those used to control for confounding	5, 6
		(*b*) Describe any methods used to examine subgroups and interactions	5
		(*c*) Explain how missing data were addressed	4, 13
		(*d*) *Cohort study*—If applicable, explain how loss to follow-up was addressed<br>*Case-control study*—If applicable, explain how matching of cases and controls was addressed	13

For peer review only

Cross-sectional study—If applicable, describe analytical methods taking account of sampling strategy	
(*e*) Describe any sensitivity analyses	13

Continued on next page

For peer review only

**Results**			
Participants	13*	(a) Report numbers of individuals at each stage of study—eg numbers potentially eligible, examined for eligibility, confirmed eligible, included in the study, completing follow-up, and analysed	3, 4, Figure S1
		(b) Give reasons for non-participation at each stage	3, 4, Figure S1
		(c) Consider use of a flow diagram	Figure S1
Descriptive data	14*	(a) Give characteristics of study participants (eg demographic, clinical, social) and information on exposures and potential confounders	6, 7
		(b) Indicate number of participants with missing data for each variable of interest	4, Figure S1
		(c) *Cohort study*—Summarise follow-up time (eg, average and total amount)	5
Outcome data	15*	*Cohort study*—Report numbers of outcome events or summary measures over time<br>*Case-control study*—Report numbers in each exposure category, or summary measures of exposure<br>*Cross-sectional study*—Report numbers of outcome events or summary measures	7, 8
Main results	16	(*a*) Give unadjusted estimates and, if applicable, confounder-adjusted estimates and their precision (eg, 95% confidence interval). Make clear which confounders were adjusted for and why they were included	8-10
		(*b*) Report category boundaries when continuous variables were categorized	4, 5, Tables 1 and 2
		(*c*) If relevant, consider translating estimates of relative risk into absolute risk for a meaningful time period	Tables 3 and 4
Other analyses	17	Report other analyses done—eg analyses of subgroups and interactions, and sensitivity analyses	9, 12, 13 Tables S2-4
**Discussion**			
Key results	18	Summarise key results with reference to study objectives	10
Limitations	19	Discuss limitations of the study, taking into account sources of potential bias or imprecision. Discuss both direction and magnitude of any potential bias	12, 13
Interpretation	20	Give a cautious overall interpretation of results considering objectives, limitations, multiplicity of analyses, results from similar studies, and other relevant evidence	11-13
Generalisability	21	Discuss the generalisability (external validity) of the study results	13
**Other information**			
Funding	22	Give the source of funding and the role of the funders for the present study and, if applicable, for the original study on which the present article is based	14

*Give information separately for cases and controls in case-control studies and, if applicable, for exposed and unexposed groups in cohort and cross-sectional studies.

For peer review only

Note: An Explanation and Elaboration article discusses each checklist item and gives methodological background and published examples of transparent reporting. The STROBE checklist is best used in conjunction with this article (freely available on the Web sites of PLoS Medicine at http://www.plosmedicine.org/, Annals of Internal Medicine at http://www.annals.org/, and Epidemiology at http://www.epidem.com/). Information on the STROBE Initiative is available at www.strobe-statement.org.

For peer review only
